# Prenatal exposure to common infections and newborn DNA methylation: A prospective, population-based study

**DOI:** 10.1016/j.bbi.2024.07.046

**Published:** 2024-07-29

**Authors:** Anna Suleri, Kristina Salontaji, Mannan Luo, Alexander Neumann, Rosa H. Mulder, Henning Tiemeier, Janine F. Felix, Riccardo E. Marioni, Veerle Bergink, Charlotte A.M. Cecil

**Affiliations:** a Department of Child and Adolescent Psychiatry/Psychology, Erasmus MC University Medical Center, the Netherlands; b The Generation R Study Group, Erasmus MC University Medical Center, Rotterdam, the Netherlands; c Department of Epidemiology, Erasmus MC University Medical Center, Rotterdam, the Netherlands; d Department of Social and Behavioral Sciences, Harvard T.H. Chan School of Public Health, Boston, MA, USA; e Department of Pediatrics, Erasmus MC University Medical Center Rotterdam, Rotterdam, the Netherlands; f Centre for Genomic and Experimental Medicine, Institute of Genetics and Cancer, University of Edinburgh, Edinburgh EH4 2XU, UK; g Department of Psychiatry, Icahn School of Medicine at Mount Sinai, NY, USA; h Department of Psychiatry, Erasmus MC University Medical Center, Rotterdam, the Netherlands; i Department of Biomedical Data Sciences, Molecular Epidemiology, Leiden University Medical Center, Leiden, the Netherlands

**Keywords:** Maternal immune activation, Epigenetics, Maternal infections, DNA methylation, Generation R, ALSPAC, Asthma, Mental health, Child development

## Abstract

**Background:**

Infections during pregnancy have been robustly associated with adverse mental and physical health outcomes in offspring, yet the underlying molecular pathways remain largely unknown. Here, we examined whether exposure to common infections *in utero* associates with DNA methylation (DNAm) patterns at birth and whether this in turn relates to offspring health outcomes in the general population.

**Methods:**

Using data from 2,367 children from the Dutch population-based Generation R Study, we first performed an epigenome-wide association study to identify differentially methylated sites and regions at birth associated with prenatal infection exposure. We also examined the influence of infection timing by using self-reported cumulative infection scores for each trimester. Second, we sought to develop an aggregate methylation profile score (MPS) based on cord blood DNAm as an epigenetic proxy of prenatal infection exposure and tested whether this MPS prospectively associates with offspring health outcomes, including psychiatric symptoms, BMI, and asthma at ages 13–16 years. Third, we investigated whether prenatal infection exposure associates with offspring epigenetic age acceleration – a marker of biological aging. Across all analysis steps, we tested whether our findings replicate in 864 participants from an independent population-based cohort (ALSPAC, UK).

**Results:**

We observed no differentially methylated sites or regions in cord blood in relation to prenatal infection exposure, after multiple testing correction. 33 DNAm sites showed suggestive associations (p < 5e10 – 5; of which one was also nominally associated in ALSPAC), indicating potential links to genes associated with immune, neurodevelopmental, and cardiovascular pathways. While the MPS of prenatal infections associated with maternal reports of infections in the internal hold out sample in the Generation R Study ($R_{incremental}^{2}\;=\;0.049$), it did not replicate in ALSPAC ($R_{incremental}^{2}\;=\;0.001$), and it did not prospectively associate with offspring health outcomes in either cohort. Moreover, we observed no association between prenatal exposure to infections and epigenetic age acceleration across cohorts and clocks.

**Conclusion:**

In contrast to prior studies, which reported DNAm differences in offspring exposed to severe infections *in utero*, we do not find evidence for associations between self-reported clinically evident common infections during pregnancy and DNAm or epigenetic aging in cord blood within the general pediatric population. Future studies are needed to establish whether associations exist but are too subtle to be statistically meaningful with present sample sizes, whether they replicate in a cohort with a more similar infection score as our discovery cohort, whether they occur in different tissues than cord blood, and whether other biological pathways may be more relevant for mediating the effect of prenatal common infection exposure on downstream offspring health outcomes.

## 1. Introduction

Exposure to prenatal infections is associated with a range of adverse developmental and health outcomes in offspring (Estes and McAllister, 2016 Aug 19; Han et al., 2021 Jan 21). For example, evidence from clinical and population-based studies points towards a link between prenatal infection exposure and birth complications (e.g., premature birth (Kumar et al., 2022 Jun), physical health problems (e.g., metabolic syndrome (Kumar et al., 2022 Jun), and asthma (Collier et al., 2013 Dec), as well as neurodevelopmental disorders (e.g., ADHD and schizophrenia (Khandaker et al., 2013 Feb; Hall et al., 2021; Suleri et al., 2023 Jun; Al-Haddad et al., 2019 Jun 1) in offspring. While some of these effects may be transient, longitudinal studies suggest that prenatal infection exposure can have lasting effects on offspring health (Han et al., 2021 Sep; al-Haddad BJS, Jacobsson B, Chabra S, Modzelewska D, Olson EM, Bernier R, et al. Long-term Risk of Neuropsychiatric Disease After Exposure to Infection In Utero. JAMA Psychiatry., et al., 2019; Shimizu et al., 2023 Feb 25), for example our prior work showed stable associations with elevated emotional and behavioral symptoms from toddlerhood into adolescence (Suleri et al., 2023). Broadly, it is known that infections can influence offspring *in utero* through both direct and indirect routes (Han et al., 2021 Sep). Certain infectious pathogens (e.g., Cytomegalovirus or Herpes Simplex) can directly pass the placenta and blood–brain barrier, disrupting processes such as fetal neuronal migration and synapse formation (Han et al., 2021 Sep). Infections may also indirectly influence fetal development by activating the mother’s immune system, in turn leading to placental insufficiency, due to elevated levels of placental immune markers, and/or fetal inflammation (Han et al., 2021 Sep; Weber-Stadlbauer, 2017 May 2). Yet, through what specific molecular mechanism these acute adverse conditions can exert a lasting impact on the child remains unknown (Han et al., 2021 Sep; Shimizu et al., 2023 Feb 25; Weber-Stadlbauer, 2017 May 2; Fung et al., 2022 Oct).

Epigenetic processes in the offspring, particularly DNA methylation (DNAm) at birth, represent a potential pathway of interest (Han et al., 2021 Sep; Pradhan et al., 2023 Oct; Banik et al., 2017 May 24; Richetto et al., 2017 Feb 1; Woods et al., 2021 Oct). DNAm can regulate gene activity through the addition of methyl groups to DNA base pairs in response to internal (e.g., genetic) and external (e.g., environmental) factors. DNAm has been suggested to play a key role in typical (neuro) development and aging, while alterations in DNAm have been associated with numerous mental health and physical disorders (Rasmi et al., 2023 Jun). Consequently, DNAm at birth may act as a biological marker (and potential mediator) of environmental influences – such as prenatal exposure to infections – on health outcomes. So far, epigenetic research on prenatal infections has primarily been conducted using experimental approaches. Preclinical animal studies have reported, for example, that DNAm alterations in placental tissue of exposed mice affect expression of key genes involved in fetal development and metabolism (e.g., IGF-2) (Pradhan et al., 2023 Oct; Claycombe et al., 2015 May). DNAm changes have also been observed in immune cells (e.g., IFN-γ and IL-4, linked to early childhood diseases such as asthma (Claycombe et al., 2015 May), as well as brain cells in exposed offspring (Pradhan et al., 2023 Oct). Additionally, studies using human-derived cellular and organoid models have shown that exposure to the Zika virus leads to DNAm changes in neural cell progenitors, astrocytes, and differentiated neurons, particularly at genes implicated in neuropsychiatric disorders, such as schizophrenia (Janssens et al., 2018; Kandilya et al., 2019 Aug).

In humans, most epigenetic studies have focused on broader markers of inflammation, rather than (prenatal) exposure to infections (Deghan, 2011; Ligthart et al., 2016 Dec 12; Stevenson et al., 2020 Jul 27; Wielscher et al., 2022 May 3; Yeung et al., 2020 Apr 30). For example, several population-level, multi-cohort epigenome-wide association studies (EWAS) of C-reactive protein (CRP) levels, one of the most commonly examined inflammatory markers, have revealed associations with DNAm at a large number of sites in whole blood (Ligthart et al., 2016 Dec 12; Wielscher et al., 2022 May 3; Yeung et al., 2020 Apr 30). These sites can be aggregated into a single methylation profile score (MPS) and used as an epigenetic proxy of CRP levels, which has been associated with (neuro)developmental and health outcomes in both pediatric and adult samples (Ligthart et al., 2016 Dec 12; Wielscher et al., 2022 May 3; Yeung et al., 2020 Apr 30; Barker et al., 2018 Aug; Conole et al., 2023 Mar). In contrast, studies on prenatal infections have so far focused on severe infections (e.g., Hepatitis B virus, Zika virus, HIV, or SARS-CoV-2 virus) in small, selected samples. These have shown, for example, that prenatal exposure to a hepatitis B infection is associated with global as well as locus-specific differences in DNAm in offspring cord blood (n = 12) (Cheng et al., 2018 Jun). Another study comparing neonates with congenital Zika virus related microcephaly (n = 18) to controls (n = 20) observed differential DNAm patterns in blood between the groups, coinciding with genes involved in fetal (neuro) development and viral host immunity (Anderson et al., 2021 Feb 13). With regards to perinatally acquired HIV, two studies investigated DNAm levels in exposed children (Shiau et al., 2019 Jul 19; Shiau et al., 2021 Apr 1), reporting a large number of differentially methylated sites at mean age 7 (primarily in genes involved in adaptive immunity, including the MHC region) (n = 120) (Shiau et al., 2019 Jul 19), as well as epigenetic age acceleration (i.e., having an older epigenetic-estimated age relative to chronological age – a risk marker for poor health and age-related disease) from 11 to 17 years (n = 32) (Shiau et al., 2021 Apr 1). Lastly, two studies examined the association between prenatal exposure to SARS-CoV-2 infection and DNAm levels in cord blood (n = 19) (Urday et al., 2023) and infant buccal levels (n = 4) (Hill et al., 2023 Feb), with suggestive differentially methylated sites implicating once again adaptive immunity (MHC region), as well as genes associated with numerous diseases.

Despite this growing evidence base linking prenatal infection exposure, inflammation, and epigenetic programming in offspring (Pradhan et al., 2023 Oct), key gaps remain. First, because of the current focus on severe infections (e.g., HIV or Zika virus) in small, selected, high-risk samples, little is known about how exposure to common infections may relate to offspring DNAm levels within the general population. Second, because infection exposure is typically measured only once, it is unclear to what extent associations with offspring DNAm may vary by timing of infection. Research leveraging data from i) multiple cohorts that feature comparable measures and ii) infection exposure at multiple time points would help to assess the robustness and generalizability of observed associations. Third, no study to our knowledge has tested whether differential DNAm related to infection exposure *in utero* also associates with relevant offspring health outcomes, such as psychiatric, respiratory, or cardiometabolic phenotypes.

To address these gaps, we investigated associations between prenatal cumulative exposure to self-reported (common) infections and offspring cord blood DNAm patterns at birth, based on data from 2,338 children from the population-based Generation R Study. We first performed an EWAS analysis to identify differentially methylated sites and regions in relation to prenatal infection exposure. As a sensitivity analysis, we also examined the role of timing of infection by repeating our analyses using trimester-specific scores of infections. Second, we developed a MPS of prenatal infections based on cord blood DNAm, and tested whether this score prospectively associates with offspring health outcomes, namely child psychiatric symptoms, body mass index (BMI), and asthma, measured in early adolescence. Third, we investigated whether prenatal exposure to infections associates with epigenetic age acceleration – as a marker of biological ageing – using two gestational epigenetic clocks. Across all steps, we sought to validate any findings in 864 children from an independent population-based cohort, the Avon Longitudinal Study of Parents and Children (ALSPAC).

## 2. Methods

### 2.1. Study selection and participants

We used data from two independent population-based birth cohorts: the Generation R Study (Generation R) (Kooijman et al., 2016 Dec; Kruithof et al., 2014 Dec) as the discovery sample and the Avon Longitudinal Study of Parents and Children (ALSPAC) (Niarchou et al., 2015 Jul; Boyd et al., 2013 Feb; Fraser et al., 2013 Feb; Major-Smith et al., 2022) as the replication sample. A brief description of both cohorts can be found in Supplemental text A. Both studies were approved by the local Medical Ethics Committee and written informed consent was obtained for all participants.

To be included in our study, epigenome wide DNAm data from cord blood had to be available. Analyses were restricted to participants of European ancestry (determined by self-reported questionnaire data), as epigenetic data was only available in this group within *Generation R*. We excluded twins. For siblings, only one member was included in analyses based on data completeness – or, if equal, based on random selection. This resulted in a total analytical sample of 2,338 children for *Generation R* and 864 children for *ALSPAC*.

### 2.2. Assessment of prenatal infection exposure

*Generation R*: We used a previously constructed cumulative prenatal score of common clinically evident infections (Suleri et al., 2023 Jun). This score was derived from self-reported questionnaire data collected at three different stages of pregnancy, with one survey administered in each trimester. The questionnaire asked women to provide information on various infection-related items, including i) upper respiratory tract infections, ii) lower respiratory tract infections, iii) gastrointestinal infections, iv) cystitis/pyelitis, v) dermatitis, vi) eye infections, vii) herpes zoster, viii) influenza, ix) sexually transmitted diseases, and x) instances of fever (>38 °C/100.4°F). Based on the responses, we created four scores: one for each trimester (trimester-based) and one covering the entire pregnancy. Each reported instance of an infection condition within a trimester (‘yes’) was assigned one point, while the absence of an infection condition (‘no’) received zero points. Consequently, the total score for each trimester (maximum = 10 points), as well as the cumulative score for the entire pregnancy (maximum = 30 points), was then calculated.

The rationale for utilizing the cumulative score is two-fold: i) we hypothesize that the activation of the mother’s immune system, rather than specific effects of individual infections, may be an important pathway through which common clinically evident infections exert their effect on offspring (Han et al., 2021 Sep; Shimizu et al., 2023 Feb 25) and ii) to increase statistical power of our exposure assessment, considering the variability in prevalence across different types of infections. Of note, DNAm assessments from blood were not conducted concurrently with the self-reported infection measurements. Additionally, no biological indicators of infection were available at the time of the self-reports.

#### ALSPAC:

We constructed a corresponding cumulative score of prenatal infection. However, only information on urinary tract infections and influenza could be included (i.e., 2 out of the 8 types of infections available in Generation R). We used self-reported questionnaire data collected at 18 weeks of gestation (reporting on infections up to 4 months of pregnancy), 32 weeks of gestation (reporting on infections in month 4–7 of pregnancy) and 8 weeks postpartum (reporting on infections in month 8–9 of pregnancy) (Harris et al., 2009 Apr). We combined the information on the fourth month of pregnancy from the questionnaire at 18 weeks of gestation with the questionnaire at 32 weeks of gestation to define infections in the second trimester. As the *Generation R* and *ALSPAC* infection scores had a different scale, we standardized both scores to a mean of 0 and a SD of 1 to enable comparison. Similar to Generation R, we created both trimester-specific and total prenatal infection scores based on this data.

### 2.3. Assessment of cord blood DNAm profiles and gestational epigenetic clocks

In both cohorts, umbilical cord blood was drawn at birth. In *Generation R*, DNAm profiles were generated either with the Illumina Infinium HumanMethylation450 BeadChip array (Illumina Inc., San Diego, CA) (GenerationR_450k_: n = 1367) or with the Illumina MethylationEPIC 850 K array (Illumina Inc., San Diego, CA) (GenerationR_EPIC_: n = 971). In *ALSPAC*, all samples were profiled using the 450 k array (n = 864). Supplementary text B described the sample processing, quality control and normalization steps in detail. In both cohorts, we employed normalized, untransformed beta values that ranged from 0 (i.e. fully unmethylated) to 1 (i.e. fully methylated). Before analysis, we removed extreme outliers beyond 3 times the interquartile range from the quartile limit (i.e., 25th percentile minus 3*IQR and 75th percentile plus 3*IQR). We further excluded probes that i) mapped to X and Y chromosomes, ii) overlapped with single-nucleotide-polymorphisms, and iii) were control or cross-reactive probes (targeting repetitive sequences/co-hybridizing to alternate sequences) (Chen and an, Lemire M, Choufani S, Butcher DT, Grafodatskaya D, Zanke BW, 2013 Feb; McCartney et al., 2016 Sep).

We used two different gestational epigenetic clocks to compute estimates of epigenetic age acceleration (EAA) at birth, in order to test the robustness of associations and maximize comparability with existing studies on gestational epigenetic clocks (Monasso et al., 2021 Apr 29). The Bohlin clock uses DNAm levels from 96 specific CpG sites sourced from the 450 K array (Bohlin et al., 2016 Oct 7). The 450 K/EPIC overlap clock (Haftorn et al., 2021 Apr 19) is constructed from the 173 CpG sites that are shared between the 450 K array and the EPIC array. The selection of the sites for both clocks was guided by Lasso regression in the original studies. We used the *methylclock* package in R to compute EAA and to impute missing values, provided that less than 20 % of the CpG’s was missing based on the Bohlin and The 450 K/EPIC overlap clocks (GenerationR_450k_: 0 missing for both clocks, GenerationR_EPIC_: 10 CpGs missing for Bohlin clock [=10.4 %] and 1 CpG missing for 450 K/EPIC overlap clock [=0.6 %], ALSPAC_450k_: 0 missing for both clocks). For both clocks, we calculated *residual* EAA (in weeks), which captures the residuals from a linear regression of DNAm age on chronological gestational age (Monasso et al., 2021 Apr 29; Girchenko et al., 2017). Unlike raw EAA, which represents a simple difference score between chronological and epigenetic gestational age, residual EAA provides a measure of epigenetic gestational age acceleration that is independent of chronological age (Girchenko et al., 2017). Residual EAA values can either be positive (indicating a higher epigenetic gestational age compared to the clinical gestational age) or negative (indicating a lower epigenetic gestational age compared to the clinical gestational age).

### 2.4. Child phenotypes

As part of our MPS validation, we included information on three child health outcomes (psychiatric symptoms, BMI, asthma) that have been linked to both (i) prenatal infections and (ii) DNAm levels in cord blood in previous population-based research.

#### Generation R:

we used the parent-reported Child Behavioral Checklist (CBCL) to measure psychiatric symptoms when the child was 13–16 years old (Achenbach, 1991; Achenbach and Rescorla, 2001; Ivanova et al., 2010 Dec), resulting in dimensional scores for total behavioral problems, internalizing problems, and externalizing problems. When the child was 13–16 years old, we also measured height (m) and weight (kg) at the research center, after which BMI of the child was calculated. Subsequently, BMI-SDS was computed, adjusting for age and sex according to Dutch reference growth curves (Talma et al., 2010). Moreover, parent-reported questionnaire data was collected during this research wave to ask whether the child was previously diagnosed with asthma (yes/no).

#### ALSPAC:

we used the Strengths and Difficulties Questionnaire (SDQ) to obtain information on general behavioral symptom scales, specifically for total difficulties, emotional problems, hyperactivity/inattention, and conduct problems, when the child was mean 15 years old (Kersten et al., 2016 Jan). Height (m) and weight (kg) were measured when the child visited the research center at mean age 17 years, after which the BMI was calculated. A parent-reported questionnaire data was administered when the child was 13 years old to inquire about any prior asthma diagnosis.

### 2.5. Covariates

We adjusted for the following potential confounders: maternal age at delivery, maternal education, maternal smoking during pregnancy, parity, gestational age at birth, child sex, batch effects and methylation-derived estimated white cell type proportions. Details per cohort are provided below.

#### Generation R:

maternal age at enrollment was calculated based on the difference between the mother’s date of birth and the date of enrollment in the study. Parity and maternal education were prospectively assessed with a questionnaire at enrollment. Maternal tobacco use (‘no’, ‘yes, until pregnancy was known,’ and ‘yes, continued during pregnancy’) was assessed using questionnaires in all three trimesters. Based on Statistics Netherlands classifications, three categories were created for maternal education: ‘primary’ (no education or primary school), ‘intermediate’ (secondary school or lower vocational training) and ‘high’ (higher vocational training or university). Gestational age at birth (weeks) was calculated based on the date of the mother’s last menstrual period or from ultrasound. Child sex was obtained from medical records. Sample plate was used to adjust for batch effects (technical covariate). To adjust for white blood cell type proportions (B-cells, CD4-T cells, CD8-T cells, granulocytes, monocytes, natural-killer cells, nucleated red blood cells), we used a cord-blood specific method (Gervin et al., 2019 Aug 27).

#### ALSPAC:

maternal age at enrollment was calculated based on the difference between the mother’s date of birth and the date of enrollment in the study. Information on maternal tobacco use during pregnancy (‘never’, ‘stopped smoking’, ‘continued smoking during pregnancy’) and parity was obtained through self-reported questionnaires. Maternal education was measured in line with the ISCED system (‘no education’ = 0, ‘compulsory schooling [CSE] = 1, ‘O-level’ = 2, ‘University’ = 3). Gestational age at birth was calculated based on the date of the mother’s last menstrual period or from ultrasound. The gestational age at birth (in weeks) was determined either using the mother’s last menstrual period or ultrasound measurements. Child sex was obtained from hospital registries. To adjust for batch effects, we used 20 surrogate variables (sva function from meffil package) (Min et al., 2018 Dec 1). Blood cell type proportions (B-cells, CD4-T cells, CD8-T cells, granulocytes, monocytes, natural-killer cells, nucleated red blood cells) were estimated with a cord-blood specific method (Gervin et al., 2019 Aug 27).

### 2.6. Statistical analysis

#### 2.6.1. EWAS of prenatal infection exposure

**Fig. 1.**
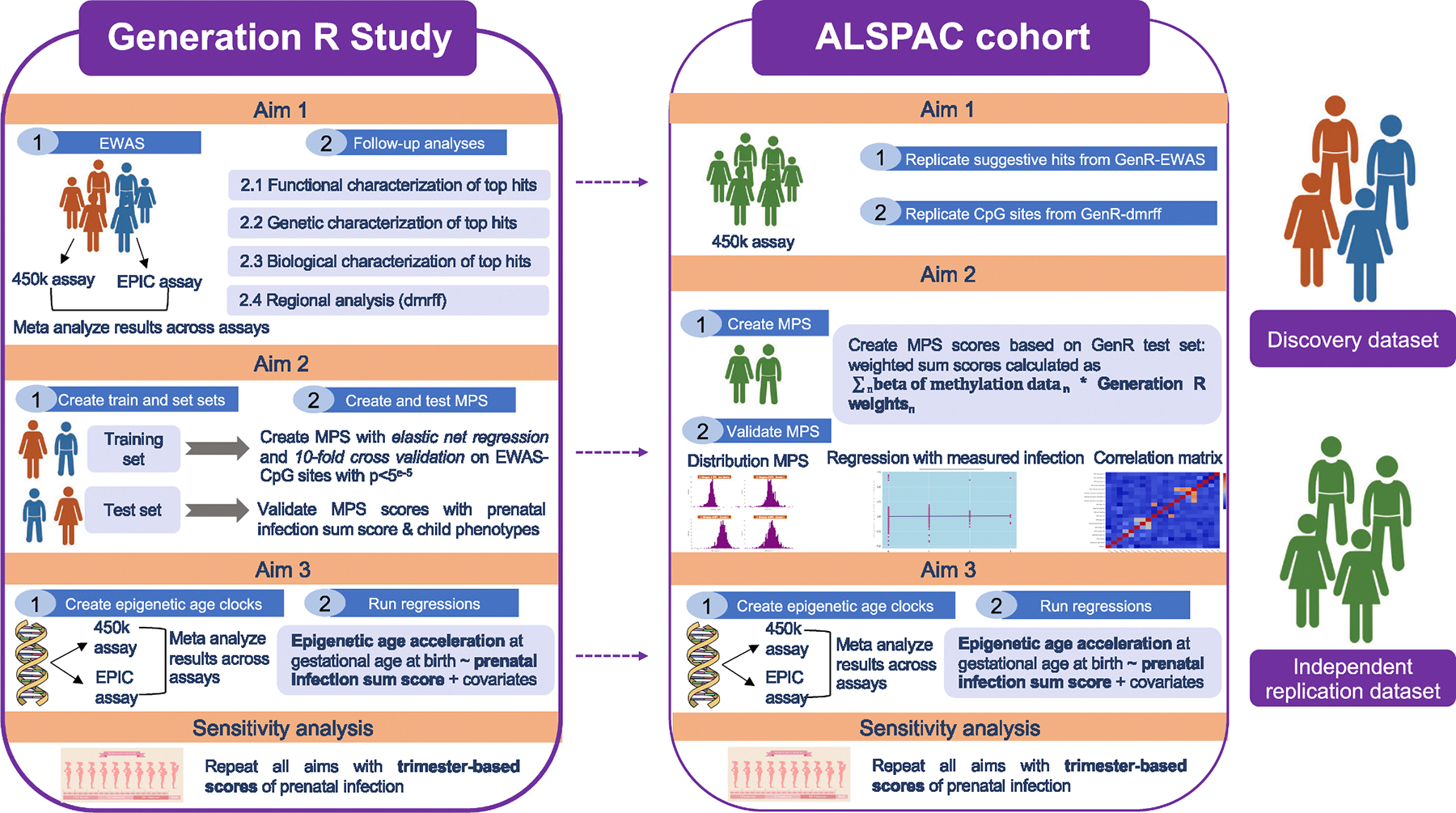
Statistical analysis overview.

For our first aim, we conducted a probe-level EWAS to identify CpG sites at birth (outcome) associated with prenatal infection exposure using linear regression models. In our discovery sample, *Generation R*, analyses were restricted to overlapping CpG sites (n = 393,360) between the 450 K array and the EPIC array and run separately per array (i.e., GenerationR_450k_ and GenerationR_EPIC_ subsamples analyzed separately). Summary statistics for these two subsamples were then pooled via a standard inverse variance weighted fixed-effects *meta*-analysis (*metafor* R package) (Viechtbauer and Cheung, 2010 Apr). To account for multiple testing, we defined genome-wide significance based on a 450 K array p-value threshold of p < 2.4e10 – 7 (Saffari et al., 2018 Feb), suggestive significance at p < 5e10 – 5, and nominal significance at p < 0.05. Top-ranking probes identified in *Generation R* (defined as those meeting a suggestive significance level threshold of p < 5e10 – 5) were then tested in *ALPSAC* as an independent replication sample (Fig. 1).

As a supplementary analysis, we ran a second EWAS within the GenerationR_EPIC_ subsample to examine associations between prenatal infection and EPIC-only CpG sites (n = 414,823). Given the lower sample size and lack of a replication sample (i.e., as both GenerationR_450k_ and ALSPAC are based on the 450 k array), as well as the dearth of previous epigenetic studies using this array in relation to infection-relevant exposures and the ubiquity of this newer array, these analyses were exploratory and performed to generate hypotheses for future research.

##### 2.6.1.1. Characterization of EWAS results.

Probes were annotated using *BiocManager* and *IlluminaHumanMethylation450kanno.ilmn12.hg19* packages in R, based on the hg19 genome build. Top-ranking probes (p < 5e10 – 5) were examined using a range of openly accessible resources, to characterize (i) potential genetic influences, (ii) associations with gene expression, (iii) enriched biological pathways, and (iv) reported links to exposures and outcomes based on published research. First, we examined whether top-ranking sites are known to associate with common genetic variants in blood (i.e., methylation quantitative trait loci analysis [mQTL]) using the GoDMC database (https://mqtldb.godmc.org.uk). We also qualitatively used the GWAS Catalog (https://www.ebi.ac.uk/gwas/) to establish whether genes annotated to top-ranking probes were (i) enriched within existing genome-wide association studies (GWAS) of infection-related traits, and (ii) have been identified as top hits for specific exposures/traits in published GWAS. Second, we used the HELIX Web catalogue (https://helixomics.isglobal.org]) to examine whether top-ranking sites are associated with gene expression levels in blood (i.e., expression quantitative trait methylation [eQTM]). Third, we examined whether genes annotated to top-ranking probes were enriched for molecular pathways and functions via gene ontology analyses (gometh and kegg; *missMethyl* R package) (Phipson et al., 2016 Jan 15). Additionally, we performed an enrichment analysis by exploring the C7 immunological signature gene sets database (https://www.gsea-msigdb.org/gsea/msigdb/). We considered a p_FDR_ value below 0.05 as significance threshold to identify independent pathways in the enrichment analyses. Finally, top-ranking probes were queried using available EWAS databases (EWASCatalog [https://www.ewascatalog.org] and EWAS Atlas [https://ngdc.cncb.ac.cn/ewas/atlas]) to identify reported associations with exposures and outcomes in published EWAS studies.

##### 2.6.1.2. Regional-level epigenome-wide analysis.

In addition to the probe-level EWAS, we performed differentially methylated region (DMR) analyses, to account for the correlated structure of DNAm and to identify broader genomic regions that are differentially methylated in relation to prenatal infections (Peters et al., 2015; Rakyan et al., 2011 Jul 12). Of note, regional analyses attenuate the burden of multiple testing and can also detect weaker signals that may be spread over wider regions. We selected potential DMRs by identifying genomic regions tagged by nominally significant CpG sites that are at most 500 bp apart. Correlations between sites are taken into account to avoid inflating regional statistics. We performed the regional analyses in *Generation R* for the 450 K and EPIC arrays separately with the *dmrff* R package and meta analyzed summary statistics across arrays with the dmrff.meta function in the package (Suderman et al., 2018). Following the standard package settings, DMRs were considered significant using the p_bonferroni_ < 0.05 as significance threshold. If DMRs were identified in *Generation R* (p_bonferroni_ < 0.05), they were then tested for replication in *ALSPAC*.

#### 2.6.2. Methylation profile score development

To address our second aim, we proceeded to develop an MPS of prenatal infections (Luo et al., 2024 Jan) (Fig. 1). The first step involved feature selection (Doherty et al., 2022). We selected prenatal infection-related CpG sites identified in our EWAS discovery analyses (i.e., in *Generation R*) at two different thresholds (nominal: p < 0.05; suggestive: p < 5e-5), to establish which threshold resulted in a better-performing MPS. Second, we split our discovery *Generation R* cohort into a training dataset (80 %, n = 1,871) and testing dataset (20 %, n = 467) with the *caret* R package (Joseph, 2022 Aug), ensuring even distribution of 450 K/EPIC array type in both datasets.

We used elastic net regularization, a machine-learning approach, to specify the prediction model and select optimal alpha and lambda features (Zou and Hastie, 2005 Apr 1) (for more information on the method, please see the extended method section in Supplementary text C).

In the *Generation R training set*, we used 10-fold cross-validation to determine the optimal combination of the hyperparameters: mixing LASSO and ridge regularization (alpha) and shrinkage/strength of regularization (lambda) using the smallest mean square error [MSE]) in the *glmnetUtils* R package (Friedman et al., 2010). Based on the optimal hyperparameters, we extracted CpGs exhibiting non-zero coefficients within the elastic net model using optimal alpha and lambda values. Then, in the *Generation R test set*, we created the MPS for prenatal infection (MPS_Prenatal_Infection_), by multiplying the methylation beta values of each selected CpG by their estimated weight (the coefficients that are the output of the elastic net regularization from the *Generation R train set*) and summing up these weighted methylation values into a single MPS score, which was then standardized.

Third, to validate the MPS_Prenatal_Infection_ in the *Generation R test set* (to prevent overfitting), we performed the following steps: i) we ran stepwise linear regression models to test whether the MPS_Prenatal_Infection_ associates as expected with the prenatal infection score (based on maternal self-reports), and whether it adds explanatory power over the use of covariates alone (incremental R^2^), and ii) we ran a regression model to examine whether the MPS_Prenatal_Infection_ at birth prospectively associates with later offspring health outcomes at age 13–16 years (i.e., child psychiatric symptoms [linear], BMI [linear] and asthma [logistic]).

Finally, we performed external validation of the MPS_Prenatal_Infection_ in *ALSPAC*. We used the weights calculated in the *Generation R training set* and multiplied those with the methylation betas of the corresponding CpG in *ALSPAC*, summing these into a single beta weighted sum score for prenatal infections. Similarly to the internal validation performed on the *Generation R test set*, we then ran regression models to establish whether the MPS_Prenatal_Infection_ associates with (i) measured prenatal infections (also over and above covariates based on incremental R^2^) and (ii) offspring health outcomes at age 15–17 years.

#### 2.6.3. Gestational epigenetic clocks

For our third aim, we applied linear regressions with the prenatal infection sum score as the exposure and residual EAA estimates as the outcome, separately for each gestational epigenetic clock (Fig. 1). In *Generation R*, we performed this analysis in the 450 K array and EPIC array samples separately, and then *meta*-analyzed the results using the *metafor* package in R. The associations between prenatal infection and both gestational epigenetic clocks were also tested in *ALSPAC*.

#### 2.6.4. Sensitivity analysis

Three sensitivity analyses were performed. First, all models (aims 1–3) were repeated using trimester-based scores of prenatal infections in both cohorts (as opposed to the total prenatal infection score), to explore the potential influence of *timing* of infection during pregnancy on offspring DNAm at birth. Second, because the prenatal infection score in *ALSPAC* contains only two domains (compared to ten in the discovery *Generation R* infection sum score) – limiting comparability between cohorts and potentially leading to discrepant findings – we created an abbreviated infection score in *Generation R* (containing only ‘flu’ and ‘urinary tract infections’ as done in ALSPAC). Top-ranking probes from our discovery EWAS analysis (aim 1) were then re-analyzed, in order to gauge the influence of using a more comprehensive vs abbreviated exposure score on infection-DNAm associations. Third, considering that cell type proportions may be influenced by infections, potentially mediating the effects of prenatal infection on DNA methylation, we conducted a sensitivity analysis of the main prenatal infection EWAS without adjusting for cell type proportions.

#### 2.6.5. Missing values and covariates

All analyses (i.e., aims 1–3) were adjusted for relevant covariates using a *core* Model 1 and an *extended* Model 2. Model 1 included the covariates: child sex, maternal age at delivery, maternal education, maternal tobacco use, parity, batch effects (sample plate), and cell type proportions. Model 2 was additionally adjusted for gestational age at birth – a variable that could qualify as potential mediator but is also strongly associated with the outcome. We further applied single imputation to impute missing exposure and covariate variables, using the *mice* R package for 30 datasets with 50 iterations (maximum missingness of exposure, covariates and child outcomes was 24 % and 16 %, respectively in *Generation R* and *ALSPAC*). Hereafter, we selected a single imputed dataset which we then used for all further analyses.

All statistical analyses were performed with R Statistical Software (version 4.3.1 R Development Core Team); the script with the code used for this project is publicly available on: https://github.com/ajsuleri/Prenatal_infections_EWAS. Moreover, the summary statistics of all EWAS results are made available on figshare (https://figshare.com/projects/Prenatal_exposure_to_common_infections_and_newborn_DNA_methylation_A_prospective_population-based_study/211576). A power analysis to calculate the minimum detectable effect size per cohort can be found in the extended method section (Supplementary Text C).

## 3. Results

**Fig. 2.**
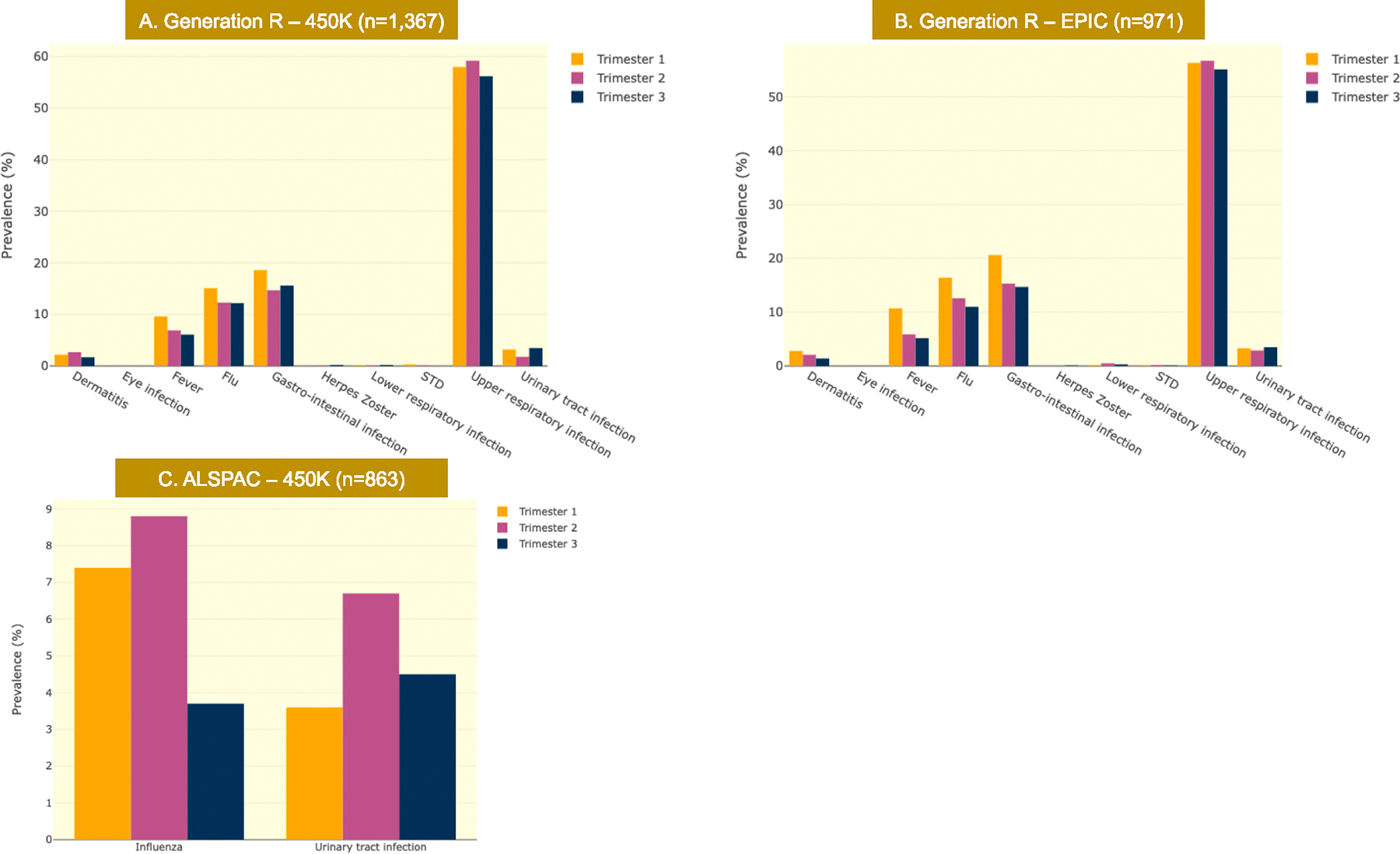
Frequency of infection types per trimester for each cohort plot.

Sample characteristics for *Generation R* (GenerationR_450k,_ n = 1,367; GenerationR_EPIC,_ n = 971) and *ALSPAC* (n = 864) can be found in Table 1. The frequency distribution of the infection sum scores in both cohorts is shown in Fig. 2.

### 3.1. Is prenatal infection exposure associated with site- and region-level DNAm in offspring at birth?

**Fig. 3.**
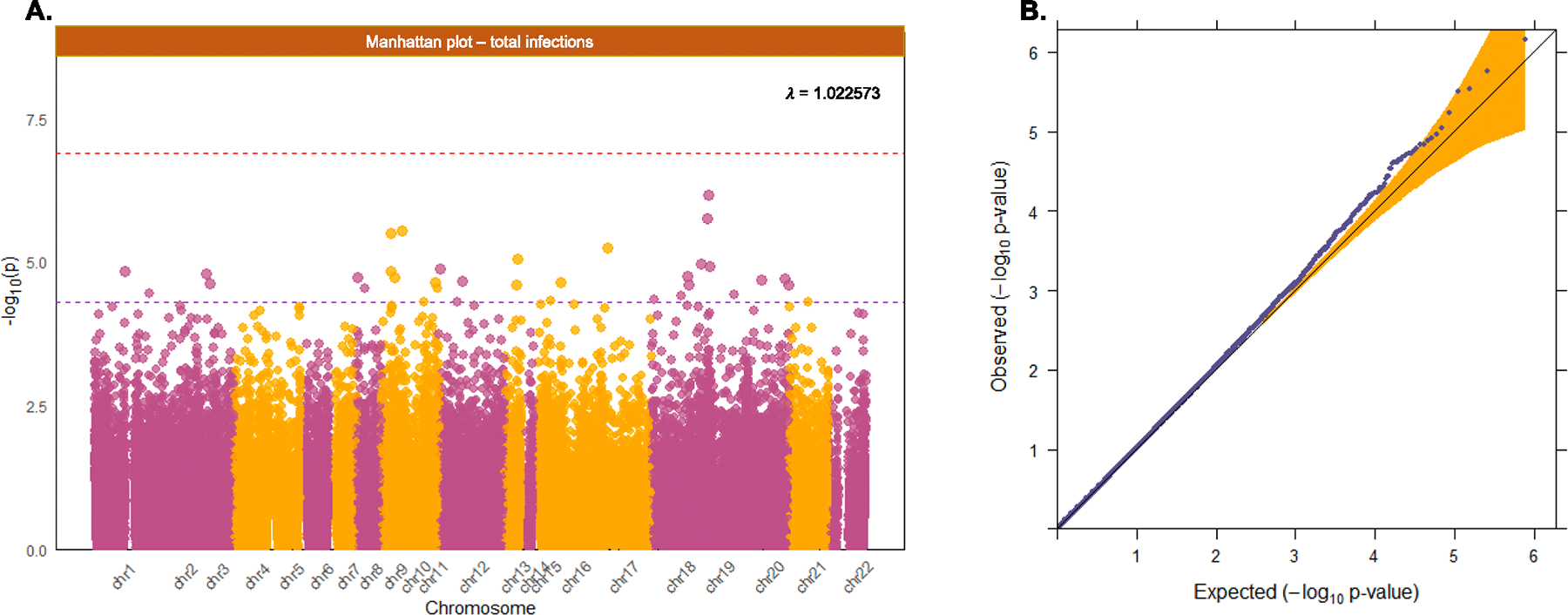
Manhattan & Q-Q plot: total infection (Generation R). Plot 3A shows the Manhattan plot in which the purple line indicates the suggestive significance threshold, and the red line indicates the Bonferroni adjusted significance threshold. Plot 3B shows the Q-Q plot.

Based on our site-level EWAS, pooling data from 2,338 mother–child dyads, we did not identify any CpG site significantly associated with prenatal exposure to infection after genome-wide correction for multiple testing (p < 2.4e10 − 7). The EWAS Manhattan plot can be found in Fig. 3A, and the QQ-plot in Fig. 3B, which showed no evidence of genomic inflation (λ = 1.023). The 33 top-ranking CpG sites associated with prenatal infection exposure at a suggestive significance threshold (p < 5e10–5) are listed in Table 2. Of note, these and following results were derived for Model 1, given that the results of models 1 and 2 were nearly the same (i.e., betas and standard errors were similar for at least the second or third decimals). In our sensitivity analyses, we found no association between the trimester-specific infection scores and DNAm levels at birth (Table S1, Figs. S1–S6). We further found no association between prenatal exposure to infections and DNAm at the 414,823 CpG sites in cord blood that are only on the EPIC array (λ = 0.977), after genome-wide correction. In the sensitivity analysis where we conducted the EWAS for total infections without adjusting for cell type proportions, consistent results were observed (see Table S2 and Fig. S7).

#### 3.1.0.1. Characterization of EWAS results

The 33 suggestive top-ranking CpG sites (i.e., p < 5e10–5) identified in our main EWAS analysis (total infection sum score) were carried forward for biological and functional characterization. Of these, 13 (39.4 %) have been linked to mQTLs, suggesting that they are at least partly under genetic control, whereas only 3 (9.1 %) were linked to gene expression (i.e., eQTMs), indicating limited associations with expression levels of the top hits in blood (Table S3). Supplementary text D and Table S3 describes the trimester-specific results. Look-up of genes that associate with the suggestive top-ranking CpG sites (i.e., p < 5e10–5) (Figs. S8–S11) showed that these genes were identified (suggestive or GWAS-specific threshold) in prior GWASes of infections such as SARS-CoV-2 and hepatitis. Pathway analyses did not identify significantly enriched biological processes, cellular components, or molecular functions associated with genes annotated to the top-ranking CpGs after FDR correction (q < 0.05). The top GO terms and KEGG pathways are included in Tables S4 and S5. The enrichment analysis for immunological gene sets showed that the gene set ‘GSE14769_UNSTIM_VS_360MIN_LPS_BMDM_DN’ showed significant enrichment (p_FDR_=0.041) for the results of the total prenatal infection score, including genes such as AMBRA1, TAPBP, VOPP1, CTU1, and PJA2 (Table S6). This gene set represents differentially expressed genes comparing unstimulated and 360 min post lipopolysaccharide-stimulated bone marrow-derived macrophages, focusing on downregulated genes. Look-up of suggestive top-ranking CpG sites in both the EWAS Catalog and EWAS Atlas indicated that most have been previously associated with inflammation-relevant traits and lifestyle factors, such as (auto-)immune conditions, asthma, cardiovascular conditions, smoking, and obesity at a suggestive significance threshold (Table S7).

#### 3.1.0.2. Regional-level epigenome-wide analysis

We observed no significant differentially methylated regions for the total infection sum score nor for the trimester-based infection sum scores after Bonferroni correction (q < 0.05).

#### 3.1.0.3. Replication of top-ranking probes in ALSPAC

None of the 33 top ranking EWAS probes identified in *Generation R* replicated in *ALSPAC* (q > 0.05) (Tables S8–S11). Before multiple testing correction, one CpG (cg01304814) showed nominal significant associations with prenatal infection exposure in *ALSPAC*, in the same direction across cohorts (β = −0.087, SE=0.034, p = 0.010). Moreover, sensitivity analyses showed that three CpGs (cg03987884, cg09130190, cg11170479) associated at a nominal significance level for trimester 2, and one CpG (cg07036524) for trimester 3 in *ALSPAC*, although these associations did not survive multiple testing correction.

The sensitivity analysis in *Generation R* using a more comparable infection score to *ALSPAC* (i.e., including only two out of ten infection domains) indicated that the use of this abbreviated score decreases the ability to identify infection-CpG associations (i.e., results in smaller effect sizes and larger standard errors) (*ρ* = 0.66 between full and abbreviated score). Only 6 of 33 of top ranking CpGs sites identified in our main analyses (using the full infection score), showed associations with the restricted infection score in Generation R using the suggestive significance for top ranking threshold (p < 5e10–5) and 16/33 of the top ranking CpG sites were significant when using the nominal significance threshold (p < 0.05) (Table S12), suggesting that the difference in infection scores may partly contribute to the lack of replicated CpGs in *ALSPAC*. Of note, the CpG site (cg01304814) that replicated in *ALSPAC* when using a nominal significance threshold, also remained significant in the *Generation R Study* when using the abbreviated infection score.

### 3.2. Can we develop a methylation profile score predicting exposure to prenatal infection in cord blood, and does this score relate to offspring health outcomes?

We selected features based on a suggestive significance threshold, as this model outperformed selection based on nominal significance. The infection sum score was equally distributed between the training and testing set in *Generation R* (Figure S12). Tables S13–S16 show the weights derived from the optimal alpha and lambda combination to further construct the MPS. In the *Generation R test set,* we found that the MPSes of infections of total pregnancy and at each trimester were significantly (p ≤ 0.001) associated with the respective infection sum scores (R^2^ for total infections = 0.049 [4.9 %]); however, the score did not associate with the infection sum scores in *ALSPAC* (R^2^ for total infections = 0.001 [1.0 %]) (Table 3, Figs. S13–S17). Moreover, the MPS did not associate with any of the child health outcomes in both cohorts (Tables S17–S20).

### 3.3. Is prenatal exposure to infections associated with epigenetic age acceleration at birth?

After multiple testing correction, we observed no association between prenatal exposure to infections and epigenetic gestational age acceleration estimated based on either the Bohlin or 450 K/EPIC clocks in *Generation R* (Table S21) and *ALSPAC* (Table S22).

## 4. Discussion

In this prospective population-based study, we examined whether prenatal exposure to self-reported clinically evident common infections (based on a cumulative score) associates with differential neonatal DNAm in cord blood, and whether this in turn relates to later offspring health outcomes. Discovery analyses were based on data from 2,367 children from the Generation R Study with trimester-specific maternal reports of infection exposure, and findings were tested for replication in 864 children from the independent ALSPAC cohort. Results from the epigenome-wide analyses did not identify differentially methylated sites or regions at birth associated with infections during pregnancy, either measured as a total or trimester-specific cumulative score. Consistent with this, an aggregate MPS capturing broader infection associated DNAm patterns did not exhibit strong explanatory power in predicting reported infection exposure or relevant offspring health outcomes, including psychiatric symptoms, BMI, and asthma in adolescence in both cohorts. Finally, we observed no association between prenatal infections and epigenetic aging, based on two gestational epigenetic clocks, in both cohorts. Overall, our findings suggest that cumulative exposure to common infections during pregnancy is unlikely to be a strong influence on offspring DNA methylation patterns in cord blood in the general population.

Prior research using preclinical models and high-risk samples has reported numerous associations between prenatal exposure to infections and offspring DNAm, highlighting its potential role as a biological mediator on downstream health outcomes (Weber-Stadlbauer, 2017 May 2; Richetto et al., 2017 Feb 1; Rasmi et al., 2023 Jun; Shiau et al., 2019 Jul 19; Shiau et al., 2021 Apr 1; P. Urday Gayen Nee’ Betal S, Sequeira Gomes R, Al-Kouatly HB, Solarin K, Chan JS, et al. SARS-CoV-2 Covid-19 Infection During Pregnancy and Differential DNA Methylation in Human Cord Blood Cells From Term Neonates Epigenetics Insights. 16, et al., 2023; Hill et al., 2023 Feb). In contrast, we did not identify any associations between prenatal infection exposure and offspring DNAm after multiple testing correction. Further, we did not observe any overlap between our findings (even at a more relaxed threshold of significance) and CpG sites or annotated genes identified in previous studies. Several factors may explain these apparent discrepancies. One relates to the type of infections investigated: while we focused on prenatal exposure to common infections, prior studies have investigated more severe exposures (i.e., continuous high-dose exposure to viral/bacterial pathogens in preclinical studies and severe infections such as HIV or Zika virus in human clinical studies). Whereas both HIV and Zika virus can directly pass the placenta and blood–brain barrier in the offspring (Radaelli et al., 2020 Apr; Martín-Bejarano et al., 2021 Dec 29), the common infections we studied cannot directly transfer to the placenta but instead can lead to placental inflammation; thus, indirectly affecting child development (Han et al., 2021 Sep). Furthermore, DNAm associated with Zika or HIV virus exposure may reflect the unique molecular mechanisms employed by these pathogens during an infection. Both Zika and HIV have the capacity to interact with host cellular machinery, potentially inducing changes in DNAm as part of the host response to infection. For instance, Zika virus has been shown to directly affect neural progenitor cells, leading to epigenetic modifications in the developing fetal brain (Kandilya et al., 2019 Aug; Anderson et al., 2021 Feb 13), whereas HIV, being a retrovirus, integrates its genetic material into the host genome, and this integration process can affect DNAm (Rasmi et al., 2023 Jun; Shiau et al., 2019 Jul 19; Shiau et al., 2021 Apr 1). Common infections may thus lack the distinctive pathogenic features that induce significant alterations in the offspring epigenome during fetal development. In our study, the use of a cumulative score for infections may have obscured any pathogen-specific associations with offspring DNAm. Additionally, by opting for a cumulative score rather than examining individual infections, our EWAS focused on exploring the shared pathway through which these infections collectively impact child neurodevelopment, rather than highlighting the unique effects of each infection individually. Moreover, because our infection score relied on self-reported data rather than directly measuring observed infection load, this approach may have contributed to the null findings in our EWAS, as self-reported data are susceptible to recall bias and may not capture all instances of infection accurately. This limitation could have obscured potential associations between infections and offspring outcomes, because the variability and severity of infections might not have been fully captured or quantified in our study. Future research employing more objective measures of infection, such as laboratory-confirmed diagnoses or biomarkers, could provide more precise insights into the relationship between infections and their effects on outcomes like DNA methylation. Moreover, further research is needed to clarify the effect of severity, chronicity, and infection type (among commonly-occurring infections) on the fetal epigenome – characteristics that we were underpowered to examine in our study.

Other methodological reasons that may explain discrepant findings include differences in (i) sample type, with our population study presenting a considerable increase in participants compared to prior work in smaller, selected samples, which may have decreased susceptibility to false positive associations; (ii) adjustment for confounders, such as maternal tobacco use or socioeconomic status, which have not typically been taken into account and may have influenced previously reported findings; and (iii) stringent multiple testing correction, which has unevenly been applied in the extant literature. Further, the use of a replication cohort in our study enabled us to evaluate the robustness and generalizability of our findings. Despite this, larger-scale multi-cohort investigations with available measures of biological immune markers (to confirm infection exposure) will be necessary to identify potentially more nuanced or infection type-specific associations with offspring DNAm. Such multi-cohort efforts, however, can be challenging due to differences in how, when and which type of infections are recorded between cohorts, complicating harmonization efforts. This is well exemplified in our study, where differences in the cumulative infection score between Generation R (10 infection domains assessed) and ALSPAC (2 domains assessed) were deemed too large for conducting a *meta*-analysis, and likely contributed to the poor replication of suggestive associations identified in Generation R (as supported by sensitivity analyses comparing associations using the full versus abbreviated infection score in Generation R). It is also possible that, rather than relating to DNAm in cord blood, prenatal exposure to infections associates with DNAm in different tissues (e.g., the brain, which is not accessible *in vivo*) or with different epigenetic mechanisms (e.g., microRNAs, histone modifications (Weber-Stadlbauer, 2017 May 2; Rasmi et al., 2023 Jun), which are also potentially important – but currently under-researched – mediators of (prenatal) environmental effects on offspring health.

Moreover, understanding the impact of prenatal infections during different trimesters of fetal development is crucial for elucidating their potential influence on neonatal health. In our study, we did not observe significant DNA methylation changes in neonates exposed to prenatal infections across any trimester. Similar to prenatal tobacco smoking (Cosin-Tomas et al., 2022 Jun 7; Monasso et al., 2020 Oct 8), it is possible that sustained exposure to infections throughout pregnancy, rather than during a specific period such as early pregnancy, may result in more adverse outcomes. In other words, since we do not identify any specific pattern, the associations may be driven by cumulative exposure across all trimesters rather than by any single trimester. While our analyses using the total score of prenatal infections also did not yield significant findings, it is not a direct measure of chronicity or repeated exposure across all trimesters. Future research should aim to integrate multi-trimester assessments of diverse prenatal exposures with comprehensive DNA methylation profiling to better comprehend their combined effects on neonatal health outcomes and to pinpoint potential sensitive periods.

While overall we find weak evidence for an association between infections during pregnancy and DNAm in cord blood based on our EWAS analyses, we note that DNAm at 33 CpG sites showed suggestive associations, several of which are mapped to genes involved in immune and (neuro)developmental processes. One of these, *MYT1L* (cpg site: cg25376660), is expressed in neuronal tissue and has been linked to multiple neurodevelopmental and psychiatric outcomes (intellectual disabilities, autism spectrum disorder, schizophrenia, and attention deficit hyperactivity disorder (Mansfield et al., 2020 Jun; Chen et al., 2022 Jul 22) as well as spontaneous preterm birth, IL-8 levels, type 2 diabetes, and systolic blood pressure (Sollis et al., 2023 Jan 6). Another gene linked to our suggestive sites, *STARD3* (cpg site: cg00264346), has previously been associated with cardiovascular disease, metabolic markers (e.g., HDL cholesterol and apolipoprotein A1), asthma, and inflammatory markers (e.g., leukocyte or neutrophil count) (Sollis et al., 2023 Jan 6). The CpG site that showed some evidence of replication in the ALSPAC cohort (i.e., nominal association in the same direction as Generation R) is annotated to the *PRKAR2A* gene (cpg site: cg01304814), which is a signaling molecule that activates cAMP and is involved in mediating anti-inflammatory cytokine production. This gene has been associated with cortical thickness, sulcal depth, depressive symptoms, intelligence, educational attainment, bone mineral density, BMI and cholesterol in prior GWASes (Sollis et al., 2023 Jan 6). Although associations did not hold after multiple testing correction on an EWAS-level, these may still point to interesting targets for future research.

Motivated by prior findings that MPSs for inflammatory markers such as CRP may capture more sustained inflammation compared to serum levels of these markers, and show utility as predictors of disease risk (Ligthart et al., 2016 Dec 12; Wielscher et al., 2022 May 3; Barker et al., 2018 Aug; Conole et al., 2023 Mar; Hillary et al., 2023; Green et al., 2021 Feb), we sought to develop an MPS of prenatal infections based on cord blood DNAm at birth. Although the score showed expected positive associations with measured infections in the Generation R testing sample (i.e., internal validation), it did not associate with measured infections in ALSPAC (i.e., external validation), and the MPS was not predictive of offspring health outcomes (psychiatric symptoms, asthma, and BMI) in early adolescence in either cohort. The contrast between well-performing MPSs for CRP versus our MPS of prenatal infection could reflect a number of factors, including differences in discovery sample size (MPSs of CRP are based on EWAS *meta*-analyses of ~ 8,000–22,000 participants) (Ligthart et al., 2016 Dec 12; Wielscher et al., 2022 May 3; Hillary et al., 2023), the number of significant epigenetic signals (with EWASs of CRP identifying numerous genome-wide significant CpG associations), overfitting (our whole discovery sample was used for the EWAS and then later split up to create the MPS), and the different design (MPSs for CRP are typically derived using weights from cross-sectional EWAS analyses where DNAm and CRP are measured at the same time point, compared to our study prospectively associating prenatal infection exposure with cord blood DNAm). Furthermore, inflammatory markers may not only reflect infection exposure, but can also be influenced by other pre-pregnancy or prenatal maternal factors such as obesity, chronic stress, or inflammatory disorders (Lee et al., 2012 Feb; Bourassa et al., 2021 Oct). Finally, MPSs of CRP are based on a single biomarker whereas we aimed to create an MPS of a dimensional score encompassing several types of common infections (as we hypothesized similar pathways through which these infections may impact child development). It is interesting that this cumulative infection score showed clear phenotypic associations with (mental) health problems between age 1.5y and 14y in a previous study within the Generation R Study, suggesting that the score itself works as a risk marker (Suleri et al., 2023), but that effects are unlikely to be mediated by cord blood DNAm (at least to the extent captured by our MPS).

As a last step, we examined whether prenatal infection exposure relates to epigenetic clock estimates, given a previous study reporting an association prenatal exposure to HIV and accelerated epigenetic aging in offspring (Shiau et al., 2021 Apr 1). No associations were observed in our study, across gestational epigenetic clocks or cohorts. A potential explanation may be the differential route through which an infection such as HIV (vertical transmission, i.e., directly passing the placenta and the offspring’s blood brain barrier) may have more direct effects in cord blood than common infections with pathogens, e.g., influenza virus, that cannot directly pass the placenta. Next to difference in severity of infection, another reason may be the difference in timing, as the prior study investigated the period between late childhood and adolescence as opposed to epigenetic gestational age at birth. Given that DNAm is temporally dynamic, and that accelerated epigenetic (gestational) ageing at birth may not mean the same thing as accelerated epigenetic ageing later in life (where it is generally considered a marker for ageing and disease risk), future longitudinal studies with repeatedly assessed DNAm will be needed to clarify whether and how prenatal infection exposure relates to epigenetic age at different developmental stages.

Our results should be interpreted in light of several limitations. First, data on prenatal infections were gathered once per trimester, and there were no concurrent blood measurements during the infection events to quantitatively validate the prenatal infections. The use of self-reported questionnaires introduces the possibility of reporter bias, as it was recalled retrospectively after each trimester. At the same time, the use of self-report questionnaires presents certain advantages. Unlike biological measurements that require a visit to the research center and may have a short half-life, these questionnaires could be conveniently filled out at home at any time. This flexibility reduces the likelihood of encountering healthy volunteer (selection) bias, where participants experiencing an infection might be less inclined to attend a research visit. Moreover, infections were measured at three time points, minimizing measurement error at one time point, which may occur with single-time point measurement reporting on the full pregnancy. Second, as 8 out of the 10 domains from the Generation R infection sum score were not available in the ALSPAC cohort, there were differences in the scoring method and number of infection types assessed between the two cohorts, limiting direct comparison and replication. To explore how these differences may have influenced our results, we generated an abbreviated infection score in Generation R including the same two domains as ALSPAC, and indeed found that the use of this abbreviated score decreased our ability to identify associations based on the suggestive sites identified in our EWAS. However, an MPS of infections using the abbreviated score in Generation R Study still performed relatively well in the test set (internal validation) but did not show associations with measured infections in ALSPAC, suggesting that differences in the scores only partly account for the lack of replication. Third, we were limited in our ability to study the role of severity (beyond including fever in our cumulative infection score), chronicity, and type of infection on the association between infections during pregnancy and DNAm levels at birth. It will be important to investigate these aspects of infections in future studies. Future studies should explore the impact of specific types of common infections during pregnancy on neonatal DNA methylation, for example, within the category of urinary tract infections (UTIs), the type of bacteria causing the UTI, the severity and spread of the infection may influence associations with offspring DNAm and downstream (neuro)developmental outcomes. Fourth, although we had a significantly larger sample size compared to prior studies, we may have been underpowered to detect more subtle effects between prenatal infection exposure and epigenetic patterns at birth. Fifth, in the future, it will be important to examine the potential role of additional factors, such as antibiotic and other medication use, in the association between prenatal infection and DNAm, as well as with other potentially relevant outcomes like eczema. Finally, given the pronounced correlation between white blood cell proportions and DNAm levels in cord blood, and the biological importance of these cells in the context of prenatal infection exposure (which can lead to elevated levels of white blood cells that may mediate the effects of infection exposure on offspring), we performed our EWAS both adjusted and unadjusted for white blood cell proportions. These analyses yielded highly comparable findings, which may suggest either that cell-type proportions are not influencing epigenetic associations with prenatal infection exposure, or that the currently available cell-type estimation panels for cord blood do not have sufficiently resolution to identify relevant cell subpopulations. Future studies should consider using more comprehensive cell-type panels, such as those provided by EpiDISH, to capture cell-type composition in cord blood at a more granular level and conduct cell-type specific EWASes. The development of an expanded cell-type reference panels for cord blood, such as those differentiating between white blood cell sub-populations, would enable a better characterization of the presence and specificity of infection-DNAm associations across a broader range of cell types.

## 5. Conclusion

In conclusion, in this large, multi-cohort effort, we found limited evidence for an association between exposure to self-reported clinical evident infections (based on a cumulative score encompassing different types of common infections) during pregnancy and DNAm in cord blood in the general population, at the level of individual DNAm sites, regions, broader methylation profiles and epigenetic clock estimates. In the future, larger multi-cohort *meta*-analyses will be needed to detect subtle associations with greater power, to enable the investigation of individual infection types that may have pathogen-specific effects and to validate reported infections with biological markers of infection exposure. Exploring different tissues (across development) and other epigenetic processes will also contribute to a better understanding of the potential association between prenatal infections and the fetal epigenome, and downstream effects on offspring health.

## Supplementary Material

Supplementary material

## Figures and Tables

**Table 1:** Baseline characteristics.

	Generation R 450 K sample (n = 1,367)	Generation R EPIC sample (n = 971)	ALSPAC 450 K sample (n = 864)

Maternal characteristics			
Age mother at enrollment (years) (mean, SD)	31.7 (4.2)	31.6 (4.2)	29.9 (4.4)
Maternal education (High) (n, %)	878 (64.2)	591 (60.9)	172 (20.9)
Continued smoking in pregnancy (n, %)	179 (13.1)	145 (14.9)	94 (10.9)
Total infection sum score (median, IQR)	3.0 (2.0 – 4.0)	3.0 (1.0 – 3.0)	0.0 (0.0 – 1.0)
Abbreviated infection sum score (median, IQR)	0.0 (0.0 – 1.0)	0.0 (0.0 – 1.0)	-
Child characteristics			
Gestational age at birth (weeks) (mean, SD)	40.2 (1.5)	40.1 (1.5)	39.5 (1.5)
Sex, female (n, %)	670 (49.0)	469 (48.3)	444 (51.4)

**Table 2:** Total infection sum score and DNA methylation at birth (suggestive hits) in the discovery dataset (Generation R cohort).

CpG	Beta	SE	P-value	Chromosome	Position	Relation to island	Nearest gene

cg17050810	0.001	2.900E-04	1.749E-06	chr6	26,108,174	S_Shelf	*HIST1H1T*
cg16060163	0.002	4.400E-04	2.745E-06	chr17	80,454,814	Island	-
cg27466042	0.002	3.300E-04	3.187E-06	chr17	36,896,370	OpenSea	*PCGF2*
cg04478698*	− 0.001	3.100E-04	6.369E-06	chr4	26,033,992	S_Shelf	-
cg12058372	0.003	5.900E-04	8.485E-06	chr20	48,252,667	OpenSea	*B4GALT5*
cg25376660	− 0.001	3.200E-04	1.316E-05	chr2	1,942,893	S_Shore	*MYT1L*
cg00702872*	− 0.002	4.500E-04	1.374E-05	chr6	3,524,580	OpenSea	-
cg09130190	0.002	3.700E-04	1.429E-05	chr11	32,115,439	S_Shelf	*RCN1*
cg00093220	0.001	2.600E-04	1.475E-05	chr1	113,258,217	S_Shore	*PPM1J*
cg00264346	0.001	1.900E-04	1.486E-05	chr17	37,793,307	Island	*STARD3*
cg26718621	0.001	1.400E-04	1.645E-05	chr5	133,747,703	Island	*CDKN2AIPNL*
cg03300596*	− 0.002	3.800E-04	1.744E-05	chr17	53,628,947	OpenSea	-
cg27426220	0.001	2.400E-04	1.848E-05	chr7	143,106,126	OpenSea	*EPHA1*
cg02296145*	− 0.001	1.600E-04	2.008E-05	chr16	1,840,711	Island	*IGFALS*
cg23962849	0.001	3.300E-04	2.03E-05	chr19	42,776,879	N_Shore	-
cg18756291	− 0.001	1.300E-04	2.064E-05	chr2	85,839,143	Island	*C2orf68*
cg01304814	− 0.001	1.600E-04	2.093E-05	chr3	48,885,189	Island	*PRKAR2A*
cg16371518	0.001	3.300E-04	2.099E-05	chr11	46,564,493	OpenSea	*AMBRA1*
cg19334350	− 0.001	0.001	2.42E-05	chr7	157,406,737	S_Shore	*PTPRN2*
cg10600568	0.001	2.600E-04	2.758E-05	chr19	51,611,605	Island	*CTU1*
cg25839724	0.003	0.001	2.76E-05	chr1	203,552,475	OpenSea	-
cg27005246	0.001	1.900E-04	2.887E-05	chr16	31,044,731	Island	*STX4*
cg05544413	0.003	0.001	2.907E-05	chr6	127,663,627	N_Shore	*ECHDC1*
cg16691158	− 0.002	0.001	2.926E-05	chr5	139,140,550	S_Shore	-
cg13904667*	− 0.002	4.500E-04	3.014E-05	chr20	44,933,759	N_Shelf	-
cg08337633	0.002	0.0001	3.054E-05	chr7	55,602,109	OpenSea	*VOPP1*
cg08795170	− 0.001	2.100E-04	3.738E-05	chr5	108,746,307	S_Shore	*PJA2*
cg26068141	3.404E-04	8.3E-05	4.424E-05	chr8	71,315,032	Island	*NCOA2*
cg13680337	0.003	0.001	4.547E-05	chr10	71,626,580	OpenSea	*COL13A1*
cg14383355*	0.006	0.002	4.556E-05	chr2	68,957,957	OpenSea	-
cg18738857	− 0.002	3.800E-04	4.59E-05	chr19	3,606,974	Island	*TBXA2R*
cg19445322	− 0.005	0.001	4.687E-05	chr3	13,246,196	Island	-
cg08725904	0.001	2.900E-04	4.773E-05	chr5	6,753,790	N_Shore	*POLS*

Of note, linear regression models were adjusted for child sex, maternal age at delivery, maternal education, maternal tobacco use, parity, batch effects (sample plate), and cell type proportions.

* Probe contains snp’s.

**Table 3:** Methylation profile scores for infections and infection sum scores.

ENR-based MPS	Standardized beta	SE	P-value	Incremental R^2^

*Generation R test set (n = 476)*				
MPS_total_infections_	0.222	0.046	1.730E-06**	0.049
MPS_trimester1_infections_	0.148	0.045	0.001**	0.023
MPS_trimester2_infections_	0.138	0.045	0.002**	0.020
MPS_trimester3_infections_	0.135	0.046	0.003**	0.018
MPS_total_infections_abbreviated_	0.253	0.051	9.900E-07**	0.062
MPS_trimester1_infections_abbreviated_	0.170	0.048	3.870E-04**	0.029
MPS_trimester2_infections_abbreviated_	0.105	0.049	0.033*	0.011
MPS_trimester3_infections_abbreviated_	0.099	0.047	0.036*	0.010
*ALSPAC data set (n = 864)*				
MPS_total_infections_	− 0.032	0.037	0.398	0.001
MPS_trimester1_infections_	0.015	0.036	0.678	2.250E-04
MPS_trimester2_infections_	− 0.045	0.039	0.249	0.002
MPS_trimester3_infections_	− 0.005	0.037	0.887	2.650E-05

Of note,_abbreviated indicates the abbreviated infection score in Generation R with the same two domains as in ALSPAC. Moreover, linear regression models were adjusted for child sex, maternal age at delivery, maternal education, maternal tobacco use, parity, batch effects (sample plate), and cell type proportions.

* P<0.05.

** P_FDR_<0.05.

## Data Availability

Requests should be directed to the management team of the Generation R Study (v.jaddoe@erasmusmc.nl). Individual-level data are not publicly available due to privacy and consent restrictions.

## References

[R1] Achenbach T, 1991. Manual for the child behavior checklist/4–18 and 1991 profile. Department of Psychiatry, University of Vermont, In Burlington, VT.

[R2] Achenbach T, Rescorla L, 2001. Manual for the ASEBA School-Age Forms & profiles. University of Vermont, Research Center for Children, Youth, & Families, Burlington.

[R3] al-Haddad BJS, Jacobsson B, Chabra S, Modzelewska D, Olson EM, Bernier R, Long-term Risk of Neuropsychiatric Disease After Exposure to Infection In Utero. JAMA Psychiatry. 2019 Jun 1;76(6):594. 30840048 10.1001/jamapsychiatry.2019.0029 PMC6551852

[R4] Al-Haddad BJS, Jacobsson B, Chabra S, Modzelewska D, Olson EM, Bernier R, , 2019 Jun 1. Long-term Risk of Neuropsychiatric Disease After Exposure to Infection In Utero. JAMA Psychiat. 76 (6), 594–602. 10.1001/jamapsychiatry.2019.0029 PMC6551852 30840048

[R5] Anderson D, Neri JICF, Souza CRM, Valverde JG, De Araújo JMG, Nascimento MDSB, , 2021 Feb 13. Zika Virus Changes Methylation of Genes Involved in Immune Response and Neural Development in Brazilian Babies Born With Congenital Microcephaly. J Infect Dis 223 (3), 435–440. 32614431 10.1093/infdis/jiaa383

[R6] Banik A, Kandilya D, Ramya S, Stünkel W, Chong YS, Dheen ST, 2017 May 24. Maternal Factors that Induce Epigenetic Changes Contribute to Neurological Disorders in Offspring. Genes 8 (6), 150. 28538662 10.3390/genes8060150 PMC5485514

[R7] Barker ED, Cecil CAM, Walton E, Houtepen LC, O’Connor TG, Danese A, , 2018 Aug. Inflammation-related epigenetic risk and child and adolescent mental health: A prospective study from pregnancy to middle adolescence. Dev. Psychopathol. 30 (3), 1145–1156. 30068408 10.1017/S0954579418000330 PMC7612578

[R8] Bohlin J, Håberg SE, Magnus P, Reese SE, Gjessing HK, Magnus MC, , 2016 Oct 7. Prediction of gestational age based on genome-wide differentially methylated regions. Genome Biol. 17 (1), 207. 27717397 10.1186/s13059-016-1063-4 PMC5054559

[R9] Bourassa KJ, Rasmussen LJH, Danese A, Eugen-Olsen J, Harrington H, Houts R, , 2021 Oct. Linking stressful life events and chronic inflammation using suPAR (soluble urokinase plasminogen activator receptor). Brain Behav. Immun. 97, 79–88. 34224821 10.1016/j.bbi.2021.06.018 PMC8453112

[R10] Boyd A, Golding J, Macleod J, Lawlor DA, Fraser A, Henderson J, , 2013 Feb. Cohort Profile: the ‘children of the 90s’–the index offspring of the Avon Longitudinal Study of Parents and Children. Int. J. Epidemiol. 42 (1), 111–127. 22507743 10.1093/ije/dys064 PMC3600618

[R11] Chen J, Yen A, Florian CP, Dougherty JD, 2022 Jul 22. MYT1L in the making: emerging insights on functions of a neurodevelopmental disorder gene. Transl. Psychiatry 12 (1), 292. 35869058 10.1038/s41398-022-02058-x PMC9307810

[R12] Chen Y., an, Lemire M, Choufani S, Butcher DT, Grafodatskaya D, Zanke BW,, , 2013 Feb. Discovery of cross-reactive probes and polymorphic CpGs in the Illumina Infinium HumanMethylation450 microarray. Epigenetics 8 (2), 203–209. 23314698 10.4161/epi.23470 PMC3592906

[R13] Cheng Q, Zhao B, Huang Z, Su Y, Chen B, Yang S, , 2018 Jun. Epigenome-wide study for the offspring exposed to maternal HBV infection during pregnancy, a pilot study. Gene 658, 76–85. 29526602 10.1016/j.gene.2018.03.025

[R14] Claycombe KJ, Brissette CA, Ghribi O, 2015 May. Epigenetics of Inflammation, Maternal Infection, and Nutrition1–3. J. Nutr. 145 (5), 1109S. 25833887 10.3945/jn.114.194639 PMC4410493

[R15] Collier CH, Risnes K, Norwitz ER, Bracken MB, Illuzzi JL, 2013 Dec. Maternal infection in pregnancy and risk of asthma in offspring. Matern. Child Health J. 17 (10), 1940–1950. 23338127 10.1007/s10995-013-1220-2

[R16] Conole ELS, Vaher K, Cabez MB, Sullivan G, Stevenson AJ, Hall J, , 2023 Mar. Immuno-epigenetic signature derived in saliva associates with the encephalopathy of prematurity and perinatal inflammatory disorders. Brain Behav. Immun. 21 (110), 322–338. 10.1016/j.bbi.2023.03.011 36948324

[R17] Cosin-Tomas M, Cilleros-Portet A, Aguilar-Lacasaña S, Fernandez-Jimenez N, Bustamante M, 2022 Jun 7. Prenatal Maternal Smoke, DNA Methylation, and Multi-omics of Tissues and Child Health. Curr Environ Health Rep. 9 (3), 502–512. 35670920 10.1007/s40572-022-00361-9 PMC9363403

[R18] Deghan A, , 2011. Meta-analysis of genome-wide association studies in >80 000 subjects identifies multiple loci for C-reactive protein levels. Circulation 123, 731–738. 21300955 10.1161/CIRCULATIONAHA.110.948570 PMC3147232

[R19] Doherty T, Dempster E, Hannon E, Mill J, Poulton R, Corcoran D, , 2022. A comparison of feature selection methodologies and learning algorithms in the development of a DNA methylation-based telomere length estimator [Internet]. Apr [cited 2023 Dec 12]. Available from: Bioinformatics http://biorxiv.org/lookup/doi/10.1101/2022.04.02.486242. 10.1186/s12859-023-05282-4 PMC10152624 37127563

[R20] Estes ML, McAllister AK, 2016 Aug 19. Maternal immune activation: Implications for neuropsychiatric disorders. Science 353 (6301), 772–777. 27540164 10.1126/science.aag3194 PMC5650490

[R21] Fraser A, Macdonald-Wallis C, Tilling K, Boyd A, Golding J, Davey Smith G, , 2013 Feb. Cohort Profile: the Avon Longitudinal Study of Parents and Children: ALSPAC mothers cohort. Int. J. Epidemiol. 42 (1), 97–110. 22507742 10.1093/ije/dys066 PMC3600619

[R22] Friedman J, Hastie T, Tibshirani R, 2010. Regularization Paths for Generalized Linear Models via Coordinate Descent. J. Stat. Softw. 33 (1), 1–22. 20808728 PMC2929880

[R23] Fung SG, Fakhraei R, Condran G, Regan AK, Dimanlig-Cruz S, Ricci C, , 2022 Oct. Neuropsychiatric outcomes in offspring after fetal exposure to maternal influenza infection during pregnancy: A systematic review. Reprod Toxicol Elmsford n. 113, 155–169. 10.1016/j.reprotox.2022.09.002 36100136

[R24] Gervin K, Salas LA, Bakulski KM, van Zelm MC, Koestler DC, Wiencke JK, , 2019 Aug 27. Systematic evaluation and validation of reference and library selection methods for deconvolution of cord blood DNA methylation data. Clin. Epigenetics 11 (1), 125. 31455416 10.1186/s13148-019-0717-y PMC6712867

[R25] Girchenko P, Lahti J, Czamara D, Knight AK, Jones MJ, Suarez A, , 2017. Associations between maternal risk factors of adverse pregnancy and birth outcomes and the offspring epigenetic clock of gestational age at birth. Clin Epigenetics. 9, 49. 28503212 10.1186/s13148-017-0349-z PMC5422977

[R26] Green C, Shen X, Stevenson AJ, Conole ELS, Harris MA, Barbu MC, , 2021 Feb. Structural brain correlates of serum and epigenetic markers of inflammation in major depressive disorder. Brain Behav. Immun. 92, 39–48. 33221487 10.1016/j.bbi.2020.11.024 PMC7910280

[R27] Haftorn KL, Lee Y, Denault WRP, Page CM, Nustad HE, Lyle R, , 2021 Apr 19. An EPIC predictor of gestational age and its application to newborns conceived by assisted reproductive technologies. Clin. Epigenetics 13 (1), 82. 33875015 10.1186/s13148-021-01055-z PMC8056641

[R28] Hall HA Speyer LG Murray AL Auyeung B Prenatal Maternal Infections and Children’s Neurodevelopment in the UK Millennium Cohort Study: A Focus on ASD and ADHD J Atten Disord. 19 2021 May 10870547211015422. 10.1177/10870547211015422 34009046

[R29] Han VX, Patel S, Jones HF, Nielsen TC, Mohammad SS, Hofer MJ, , 2021 Jan 21. Maternal acute and chronic inflammation in pregnancy is associated with common neurodevelopmental disorders: a systematic review. Transl. Psychiatry 11 (1), 71. 33479207 10.1038/s41398-021-01198-w PMC7820474

[R30] Han VX, Patel S, Jones HF, Dale RC, 2021 Sep. Maternal immune activation and neuroinflammation in human neurodevelopmental disorders. Nat. Rev. Neurol. 17 (9), 564–579. 34341569 10.1038/s41582-021-00530-8

[R31] Harris PA, Taylor R, Thielke R, Payne J, Gonzalez N, Conde JG, 2009 Apr. Research electronic data capture (REDCap)—A metadata-driven methodology and workflow process for providing translational research informatics support. J. Biomed. Inform. 42 (2), 377–381. 18929686 10.1016/j.jbi.2008.08.010 PMC2700030

[R32] Hill RA, Gibbons A, Han U, Suwakulsiri W, Taseska A, Hammet F, , 2023 Feb. Maternal SARS-CoV-2 exposure alters infant DNA methylation. Brain Behav Immun - Health. 27, 100572. 36570792 10.1016/j.bbih.2022.100572 PMC9758784

[R33] Hillary RF, Ng HK, McCartney DL, Elliott HR, Walker RM, Campbell A, , 2023. Blood-based epigenome-wide analyses of chronic low-grade inflammation across diverse population cohorts [Internet]. Nov [cited 2024 Feb 13]. Available from: Health Informatics http://medrxiv.org/lookup/doi/10.1101/2023.11.02.23298000. 10.1016/j.xgen.2024.100544 PMC11099341 38692281

[R34] Ivanova MY, Achenbach TM, Rescorla LA, Harder VS, Ang RP, Bilenberg N, , 2010 Dec. Preschool Psychopathology Reported by Parents in 23 Societies: Testing the Seven-Syndrome Model of the Child Behavior Checklist for Ages 1.5–5. J. Am. Acad. Child Adolesc. Psychiatry 49 (12), 1215–1224. 21093771 10.1016/j.jaac.2010.08.019 PMC4247330

[R35] Janssens S, Schotsaert M, Karnik R, Balasubramaniam V, Dejosez M, Meissner A, , 2018. Zika Virus Alters DNA Methylation of Neural Genes in an Organoid Model of the Developing Human Brain. mSystems. 3 (1), e00219–e317. 10.1128/mSystems.00219-17 PMC5801341 29435496

[R36] Joseph VR, 2022 Aug. Optimal ratio for data splitting. Stat Anal Data Min ASA Data Sci J. 15 (4), 531–538.

[R37] Kandilya D, Maskomani S, Shyamasundar S, Tambyah PA, Shiao Yng C, Lee RCH, , 2019 Aug. Zika virus alters DNA methylation status of genes involved in Hippo signaling pathway in human neural progenitor cells. Epigenomics 11 (10), 1143–1161. 31234652 10.2217/epi-2018-0180

[R38] Kersten P, Czuba K, McPherson K, Dudley M, Elder H, Tauroa R, , 2016 Jan. A systematic review of evidence for the psychometric properties of the Strengths and Difficulties Questionnaire. Int. J. Behav. Dev. 40 (1), 64–75.

[R39] Khandaker GM, Zimbron J, Lewis G, Jones PB, 2013 Feb. Prenatal maternal infection, neurodevelopment and adult schizophrenia: a systematic review of population-based studies. Psychol. Med. 43 (2), 239–257. 22717193 10.1017/S0033291712000736 PMC3479084

[R40] Kooijman MN, Kruithof CJ, van Duijn CM, Duijts L, Franco OH, van IJzendoorn MH,, , 2016 Dec. The Generation R Study: design and cohort update 2017. Eur. J. Epidemiol. 31 (12), 1243–1264. 28070760 10.1007/s10654-016-0224-9 PMC5233749

[R41] Kruithof CJ, Kooijman MN, van Duijn CM, Franco OH, de Jongste JC, Klaver CCW, , 2014 Dec. The Generation R Study: Biobank update 2015. Eur. J. Epidemiol. 29 (12), 911–927. 25527369 10.1007/s10654-014-9980-6

[R42] Kumar M, Saadaoui M, Al KS, 2022 Jun. Infections and Pregnancy: Effects on Maternal and Child Health. Front. Cell. Infect. Microbiol. 8 (12), 873253. 10.3389/fcimb.2022.873253 PMC9217740 35755838

[R43] Lee J, Taneja V, Vassallo R, 2012 Feb. Cigarette smoking and inflammation: cellular and molecular mechanisms. J. Dent. Res. 91 (2), 142–149. 21876032 10.1177/0022034511421200 PMC3261116

[R44] Ligthart S, Marzi C, Aslibekyan S, Mendelson MM, Conneely KN, Tanaka T, , 2016 Dec 12. DNA methylation signatures of chronic low-grade inflammation are associated with complex diseases. Genome Biol. 17 (1), 255. 27955697 10.1186/s13059-016-1119-5 PMC5151130

[R45] Luo M, Walton E, Neumann A, Thio CHL, Felix JF, Van IJzendoorn MH,, , 2024 Jan. DNA methylation at birth and lateral ventricular volume in childhood: a neuroimaging epigenetics study. J Child Psychol. Psychiatry 65 (1), 77–90. 37469193 10.1111/jcpp.13866 PMC10953396

[R46] Major-Smith D, Heron J, Fraser A, Lawlor DA, Golding J, Northstone K, 2022. The Avon Longitudinal Study of Parents and Children (ALSPAC): a 2022 update on the enrolled sample of mothers and the associated baseline data. Wellcome Open Res. 7, 283. 37664415 10.12688/wellcomeopenres.18564.2 PMC10472060

[R47] Mansfield P, Constantino JN, Baldridge D, 2020 Jun. MYT1L: A systematic review of genetic variation encompassing schizophrenia and autism. Am J Med Genet Part B Neuropsychiatr Genet off Publ Int Soc Psychiatr Genet. 183 (4), 227–233. 10.1002/ajmg.b.32781 PMC7605444 32267091

[R48] Martín-Bejarano M, Ruiz-Saez B, Martinez-de-Aragón A, Melero H, Zamora B, Malpica NA, , 2021 Dec 29. A Systematic Review of Magnetic Resonance Imaging Studies in Perinatally HIV-Infected Individuals. AIDS Rev. 23 (4), 167–185. 33735910 10.24875/AIDSRev.20000088

[R49] McCartney DL, Walker RM, Morris SW, McIntosh AM, Porteous DJ, Evans KL, 2016 Sep. Identification of polymorphic and off-target probe binding sites on the Illumina Infinium MethylationEPIC BeadChip. Genomics Data. 9, 22–24. 27330998 10.1016/j.gdata.2016.05.012 PMC4909830

[R50] Min JL, Hemani G, Davey Smith G, Relton C, Suderman M, 2018 Dec 1. Meffil: efficient normalization and analysis of very large DNA methylation datasets. Bioinforma Oxf Engl. 34 (23), 3983–3989. 10.1093/bioinformatics/bty476 PMC6247925 29931280

[R51] Monasso GS, Jaddoe VWV, De Jongste JC, Duijts L, Felix JF, 2020 Oct 8. Timing- and Dose-Specific Associations of Prenatal Smoke Exposure With Newborn DNA Methylation. Nicotine Tob. Res. 22 (10), 1917–1922. 32330269 10.1093/ntr/ntaa069 PMC7542646

[R52] Monasso GS, Küpers LK, Jaddoe VWV, Heil SG, Felix JF, 2021 Apr 29. Associations of circulating folate, vitamin B12 and homocysteine concentrations in early pregnancy and cord blood with epigenetic gestational age: the Generation R Study. Clin. Epigenetics 13 (1), 95. 33926538 10.1186/s13148-021-01065-x PMC8082638

[R53] Niarchou M, Zammit S, Lewis G, 2015 Jul. The Avon Longitudinal Study of Parents and Children (ALSPAC) birth cohort as a resource for studying psychopathology in childhood and adolescence: a summary of findings for depression and psychosis. Soc. Psychiatry Psychiatr. Epidemiol. 50 (7), 1017–1027. 26002411 10.1007/s00127-015-1072-8

[R54] Peters TJ, Buckley MJ, Statham AL, Pidsley R, Samaras K, Lord V, R,, , 2015. De novo identification of differentially methylated regions in the human genome. Epigenetics Chromatin 8, 6. 25972926 10.1186/1756-8935-8-6 PMC4429355

[R55] Phipson B, Maksimovic J, Oshlack A, 2016 Jan 15. missMethyl: an R package for analyzing data from Illumina’s HumanMethylation450 platform. Bioinforma Oxf Engl. 32 (2), 286–288. 10.1093/bioinformatics/btv560 26424855

[R56] Pradhan J, Mallick S, Mishra N, Tiwari A, Negi VD, 2023 Oct. Pregnancy, infection, and epigenetic regulation: A complex scenario. Biochim Biophys Acta BBA - Mol Basis Dis. 1869 (7), 166768. 10.1016/j.bbadis.2023.166768 37269984

[R57] Radaelli G, Lahorgue Nunes M, Bernardi Soder R, de Oliveira JM, Thays Konat Bruzzo F, Kalil Neto F, , 2020 Apr. Review of neuroimaging findings in congenital Zika virus syndrome and its relation to the time of infection. Neuroradiol J. 33 (2), 152–157. 31896285 10.1177/1971400919896264 PMC7140307

[R58] Rakyan VK, Down TA, Balding DJ, Beck S, 2011 Jul 12. Epigenome-wide association studies for common human diseases. Nat. Rev. Genet. 12 (8), 529–541. 21747404 10.1038/nrg3000 PMC3508712

[R59] Rasmi Y, Shokati A, Hassan A, Aziz SGG, Bastani S, Jalali L, , 2023 Jun. The role of DNA methylation in progression of neurological disorders and neurodegenerative diseases as well as the prospect of using DNA methylation inhibitors as therapeutic agents for such disorders. IBRO Neurosci Rep. 14, 28–37. 36590248 10.1016/j.ibneur.2022.12.002 PMC9794904

[R60] Richetto J, Massart R, Weber-Stadlbauer U, Szyf M, Riva MA, Meyer U, 2017 Feb 1. Genome-wide DNA Methylation Changes in a Mouse Model of Infection-Mediated Neurodevelopmental Disorders. Biol. Psychiatry 81 (3), 265–276. 27769567 10.1016/j.biopsych.2016.08.010

[R61] Saffari A, Silver MJ, Zavattari P, Moi L, Columbano A, Meaburn EL, , 2018 Feb. Estimation of a significance threshold for epigenome-wide association studies. Genet. Epidemiol. 42 (1), 20–33. 29034560 10.1002/gepi.22086 PMC5813244

[R62] Shiau S, Strehlau R, Wang S, Violari A, Do C, Patel F, , 2019 Jul 19. Distinct epigenetic profiles in children with perinatally-acquired HIV on antiretroviral therapy. Sci. Rep. 9 (1), 10495. 31324826 10.1038/s41598-019-46930-1 PMC6642153

[R63] Shiau S, Brummel SS, Kennedy EM, Hermetz K, Spector SA, Williams PL, , 2021 Apr 1. Longitudinal changes in epigenetic age in youth with perinatally acquired HIV and youth who are perinatally HIV-exposed uninfected. AIDS 35 (5), 811–819. 33587437 10.1097/QAD.0000000000002805 PMC7969428

[R64] Shimizu Y, Sakata-Haga H, Saikawa Y, Hatta T, 2023 Feb 25. Influence of Immune System Abnormalities Caused by Maternal Immune Activation in the Postnatal Period. Cells. 12 (5), 741. 36899877 10.3390/cells12050741 PMC10001371

[R65] Sollis E, Mosaku A, Abid A, Buniello A, Cerezo M, Gil L, , 2023 Jan 6. The NHGRI-EBI GWAS Catalog: knowledgebase and deposition resource. Nucleic Acids Res. 51 (D1), D977–D985. 36350656 10.1093/nar/gkac1010 PMC9825413

[R66] Stevenson AJ, McCartney DL, Hillary RF, Campbell A, Morris SW, Bermingham ML, , 2020 Jul 27. Characterisation of an inflammation-related epigenetic score and its association with cognitive ability. Clin. Epigenetics 12 (1), 113. 32718350 10.1186/s13148-020-00903-8 PMC7385981

[R67] Suderman M, Staley JR, French R, Arathimos R, Simpkin A, Tilling K, 2018. dmrff: identifying differentially methylated regions efficiently with power and control [Internet]. Dec [cited 2023 Dec 12]. Available from: Bioinformatics http://biorxiv.org/lookup/doi/10.1101/508556.

[R68] Suleri A, Rommel A, Neumann A, Luo M, Hillegers M, De Witte L, Exposure to prenatal infection and the development of internalizing and externalizing problems in children: a longitudinal population-based study. J Child Psychol Psychiatry. 2023 Dec 30;jcpp.13923. 10.1111/jcpp.13923 PMC7616076 38158849

[R69] Suleri A, White T, Blok E, Cecil CAM, Reiss I, Jaddoe VWV, , 2023 Jun. The Association Between Prenatal Infection and Adolescent Behavior: Investigating Multiple Prenatal, Perinatal, and Childhood Second Hits. J. Am. Acad. Child Adolesc. Psychiatry S0890856723003258. 10.1016/j.jaac.2023.06.009 PMC10981534 37400063

[R70] Talma H, Schönbeck Y, Bakker B, HiraSing R, van Buuren S, 2010. Handleiding bij het meten en wegen van kinderen en het invullen van groeidiagrammen. TNO Kwaliteit van Leven, Leiden.

[R71] Urday P Gayen Nee’ Betal S, Sequeira Gomes R, Al-Kouatly HB, Solarin K, Chan JS, SARS-CoV-2 Covid-19 Infection During Pregnancy and Differential DNA Methylation in Human Cord Blood Cells From Term Neonates Epigenetics Insights. 16 2023 Jan 25168657231184665. 10.1177/25168657231184665 PMC10328022 37425024

[R72] Viechtbauer W, Cheung MWL, 2010 Apr. Outlier and influence diagnostics for meta-analysis. Res. Synth. Methods 1 (2), 112–125. 26061377 10.1002/jrsm.11

[R73] Weber-Stadlbauer U, 2017 May 2. Epigenetic and transgenerational mechanisms in infection-mediated neurodevelopmental disorders. Transl. Psychiatry 7 (5), e1113–e. 28463237 10.1038/tp.2017.78 PMC5534947

[R74] Wielscher M, Mandaviya PR, Kuehnel B, Joehanes R, Mustafa R, Robinson O, , 2022 May 3. DNA methylation signature of chronic low-grade inflammation and its role in cardio-respiratory diseases. Nat. Commun. 13 (1), 2408. 35504910 10.1038/s41467-022-29792-6 PMC9065016

[R75] Woods RM, Lorusso JM, Potter HG, Neill JC, Glazier JD, Hager R, 2021 Oct. Maternal immune activation in rodent models: A systematic review of neurodevelopmental changes in gene expression and epigenetic modulation in the offspring brain. Neurosci. Biobehav. Rev. 129, 389–421. 34280428 10.1016/j.neubiorev.2021.07.015

[R76] Yeung EH, Guan W, Zeng X, Salas LA, Mumford SL, de Prado BP, , 2020 Apr 30. Cord blood DNA methylation reflects cord blood C-reactive protein levels but not maternal levels: a longitudinal study and meta-analysis. Clin Epigenetics. 12 (1), 60. 32354366 10.1186/s13148-020-00852-2 PMC7193358

[R77] Zou H, Hastie T, 2005 Apr 1. Regularization and Variable Selection Via the Elastic Net. J. r. Stat. Soc. Ser. B Stat Methodol. 67 (2), 301–320.

