# Supplementary material for: Prenatal exposure to common infections and newborn DNA methylation: A prospective, population-based study
Source: Brain Behav Immun. Author manuscript; Available in PMC 2025 Jan 31. (PMC11784989; doi:10.1016/j.bbi.2024.07.046)
Supplement: Supplementary material [file NIHMS2048130-supplement-Supplementary_material.docx]

# **Prenatal exposure to common infections and newborn DNA methylation:**

# **A prospective, population-based study**

Anna Suleri^1,2^, Kristina Salontaji^1,2#^, Mannan Luo^1,2#^, Alexander Neumann^1,2^, Rosa H. Mulder^1,2^, Henning Tiemeier^3,4^, Janine F. Felix^2,5^, Riccardo E. Marioni^6^, Veerle Bergink^7,8^, Charlotte A.M. Cecil^1,4,9*^

^1^Department of Child and Adolescent Psychiatry/Psychology, Erasmus MC University Medical Center, The Netherlands.

^2^The Generation R Study Group, Erasmus MC University Medical Center, Rotterdam, the Netherlands.

^3^Department of Epidemiology, Erasmus MC University Medical Center, Rotterdam, The Netherlands.

^4^Department of Social and Behavioral Sciences, Harvard T.H. Chan School of Public Health, Boston, MA, USA.

^5^Department of Pediatrics, Erasmus MC University Medical Center Rotterdam, Rotterdam, The Netherlands.

^6^Centre for Genomic and Experimental Medicine, Institute of Genetics and Cancer, University of Edinburgh, Edinburgh, EH4 2XU, UK.

^7^Department of Psychiatry, Icahn School of Medicine at Mount Sinai, New York, USA.

^8^Department of Psychiatry, Erasmus MC University Medical Center, Rotterdam, The Netherlands.

^9^Department of Biomedical Data Sciences, Molecular Epidemiology, Leiden University Medical Center, Leiden, The Netherlands.

#Equal contribution

*Corresponding author: [c.cecil@erasmusmc.nl](mailto:c.cecil@erasmusmc.nl)

Number of supplementary figures: 17

Number of supplementary tables: 22

## **SUPPLEMENTARY INFORMATION**

## ***Supplementary text A***

Generation R

The Generation R Study is an ongoing population-based cohort that investigates the health of mothers and children from fetal stage onwards (1,2). Pregnant women with due dates from April 2002 to January 2006 living in Rotterdam, the Netherlands, were recruited for the study, resulting in a sample of 9,778 mothers and 9,901 children. Hereafter, the children and mothers have been in ongoing follow-up with in-person assessments every 4 years. More information about the Generation R Study can be found elsewhere (2). Written informed consent was obtained for all participants. The Generation R Study is conducted in accordance with the World Medical Association Declaration of Helsinki and was approved by the Medical Ethical Committee of Erasmus MC University Medical Center Rotterdam.

ALSPAC

ALSPAC represents a population-based cohort study situated in the Southwest of England, United Kingdom. The study invited pregnant women who were residents of Avon, UK and had expected delivery dates between April 1, 1991, and December 31, 1992, to participate. The initial enrollment consisted of 14,541 pregnancies (for which at least one questionnaire had been returned or a "Children in Focus" clinic had been attended by July 19, 1999). As the oldest children reached approximately 7 years of age, efforts were made to supplement the initial sample by including eligible cases that had not originally participated in the study. Consequently, the cumulative sample size after the age of seven reached 15,454 pregnancies, corresponding to 15,589 fetuses. Extensive and comprehensive information has been systematically gathered on these women, their partners, and their offspring at regular intervals, extending up to the present time. It is noteworthy that comprehensive details about the available data are accessible via a fully searchable data dictionary and variable search tool on the study's website: <http://www.bristol.ac.uk/alspac/researchers/our-data/>. In alignment with ethical principles, written informed consent has been duly obtained from all participants enrolled in the ALSPAC study. The study has been granted ethical approval by the ALSPAC Ethics and Law Committee as well as the Local Research Ethics Committees. Consent for the use of biological samples has been acquired in compliance with the Human Tissue Act (2004). Furthermore, participants have provided informed consent for the utilization of data collected through questionnaires and clinics, following the guidelines and recommendations of the ALSPAC Ethics and Law Committee during the respective time.

## ***Supplementary text B***

Generation R

DNA extracted (using the salting-out method) from blood samples taken at birth (cord blood). 500 ng DNA per sample underwent bisulfite conversion using the EZ-96 DNA Methylation kit (Shallow) (Zymo Research Corporation, Irvine, USA). Samples were plated onto 96-well plates in no specific order. For assessing DNA methylation in cord blood, we employed the Illumina Infinium® HumanMethylation450 and EPICv1 BeadChip technology. The preparation and normalization of DNA methylation data were executed following the CPACOR workflow (3) utilizing the R software package (The R Core Team, 2013). In detail, the idat files were read using the minfi package (4). Probes with a detection p-value above background (based on the sum of methylated and unmethylated intensity values) greater than or equal to 1E-16 were designated as missing for each array. Following this step, the intensity values were stratified based on autosomal and non-autosomal probes and subsequently quantile normalized for each of the six probe type categories: type II red/green, type I methylated red/green, and type I unmethylated red/green. Beta values were computed as the ratio of the methylated intensity value to the sum of methylated, unmethylated, and an offset of 100 intensities. Arrays exhibiting technical issues, such as problems with bisulfite conversion, hybridization, or extension, were excluded from subsequent analyses. Arrays where the sex determination based on the chromosome X and Y probe intensities did not match the sex of the proband were also removed. Furthermore, only arrays with a sample call rate exceeding 95% and 96% for the 450k and EPICv1 arrays, respectively were taken forward for further processing.

ALSPAC

The techniques employed for measuring methylation in the ALSPAC study have been previously outlined by Relton et al (5). In brief, cord blood was collected following established procedures. DNA methylation arrays and preliminary data processing were conducted at the University of Bristol under the framework of the ARIES project. DNA extraction adhered to standard protocols, and bisulfite conversion was performed using the Zymo EZ DNA MethylationTM kit from Zymo (Irvine, CA). The assessment of DNA methylation was carried out utilizing the Infinium HM450 BeadChip array by Illumina Inc. (San Diego, CA), in accordance with established protocols. The arrays were subjected to scanning using an Illumina iScan instrument. An initial evaluation of data quality was conducted utilizing GenomeStudio (version 2011.1). To minimize the potential for batch-related confounding effects, a semi-random allocation approach was adopted to distribute ARIES samples across slides. Specific sampling criteria were implemented to ensure the representation of all time points on each array. Normalization of the data was performed using the meffil R package (6), employing the functional normalization method.

## ***Supplementary text C – extended method section***

**Statistical analysis**

Elastic net regression is a method often used in epigenetic data to create MPSs, specifically when dealing with a high number of features (7), as it enables to adjust for their potential collinearity (7). The elastic net penalty blends characteristics of both LASSO (Least Absolute Shrinkage Operator) and ridge regression. While the LASSO method yields a sparse solution by selecting a minimal set of non-zero coefficients from the feature set (including one feature from a set of correlated features), ridge regression shrinks the coefficients for correlated features toward each other, accommodating a larger number of features.

**Power analysis**

We performed a power analysis with G*Power to calculate the required power for detecting the effect size of our top ranking CpG at a significant threshold. For an alpha of 0.00000024 (EWAS significance threshold after multiple testing), with 80% power, 10 predictors, and a beta effect size of 0.002, we would need a sample size of 22,318.

## ***Supplementary text D – extended results section***

### **3.1 Is prenatal infection exposure associated with probe- and region-level DNAm in offspring at birth?**

*Characterization of EWAS results for the trimester specific results*

55% (11/20) CpG sites were linked to mQTLs for the trimester 1 infection sum score, 33.3% (8/24) CpG sites were linked to mQTLs for the trimester 2 infection sum score, and 66.7% (18/27) CpG sites were linked to mQTLs for the trimester 3 infection sum score. 5% (1/20) of the CpGs were eQTMs for the trimester 1 infection sum score, 8.3% (2/24) of the CpGs were eQTMs for the trimester 2 infection sum score and 0% of the CpGs were eQTMs for the trimester 3 infection sum score. Look-up of suggestive CpG sites in both the EWAS Catalog and EWAS Atlas indicated that most have been previously associated with inflammation-relevant traits and lifestyle factors, such as (auto-)immune conditions, asthma, cardiovascular conditions, smoking, and obesity.

## **Supplementary figures**

**Figure S1**. Manhattan plot: trimester 1 infections (Generation R). The purple line indicates the suggestive significance threshold, and the red line indicates the Bonferroni adjusted significance threshold.


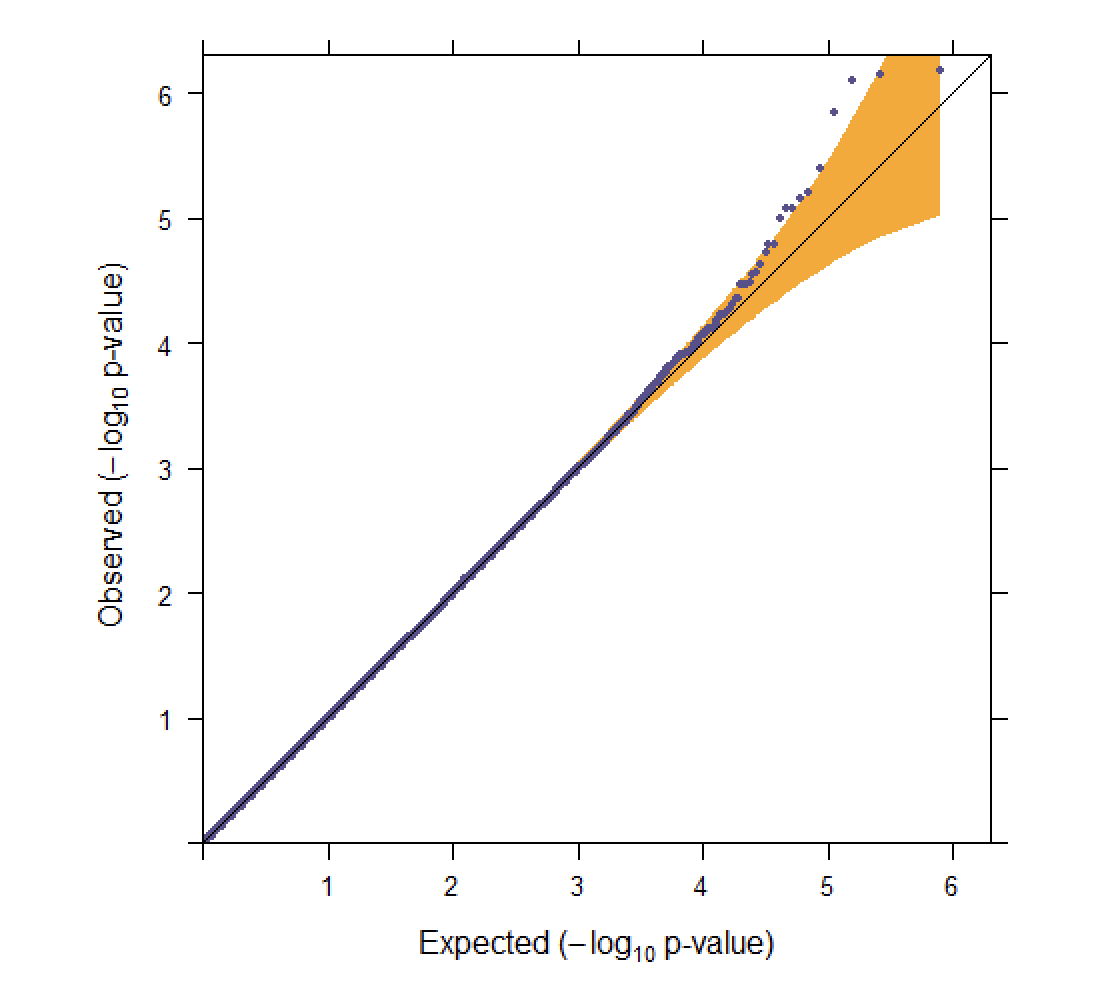


**Figure S2**. Q-Q plot: trimester 1 infections (Generation R).

**Figure S3**. Manhattan plot: trimester 2 infections (Generation R). The purple line indicates the suggestive significance threshold, and the red line indicates the Bonferroni adjusted significance threshold.


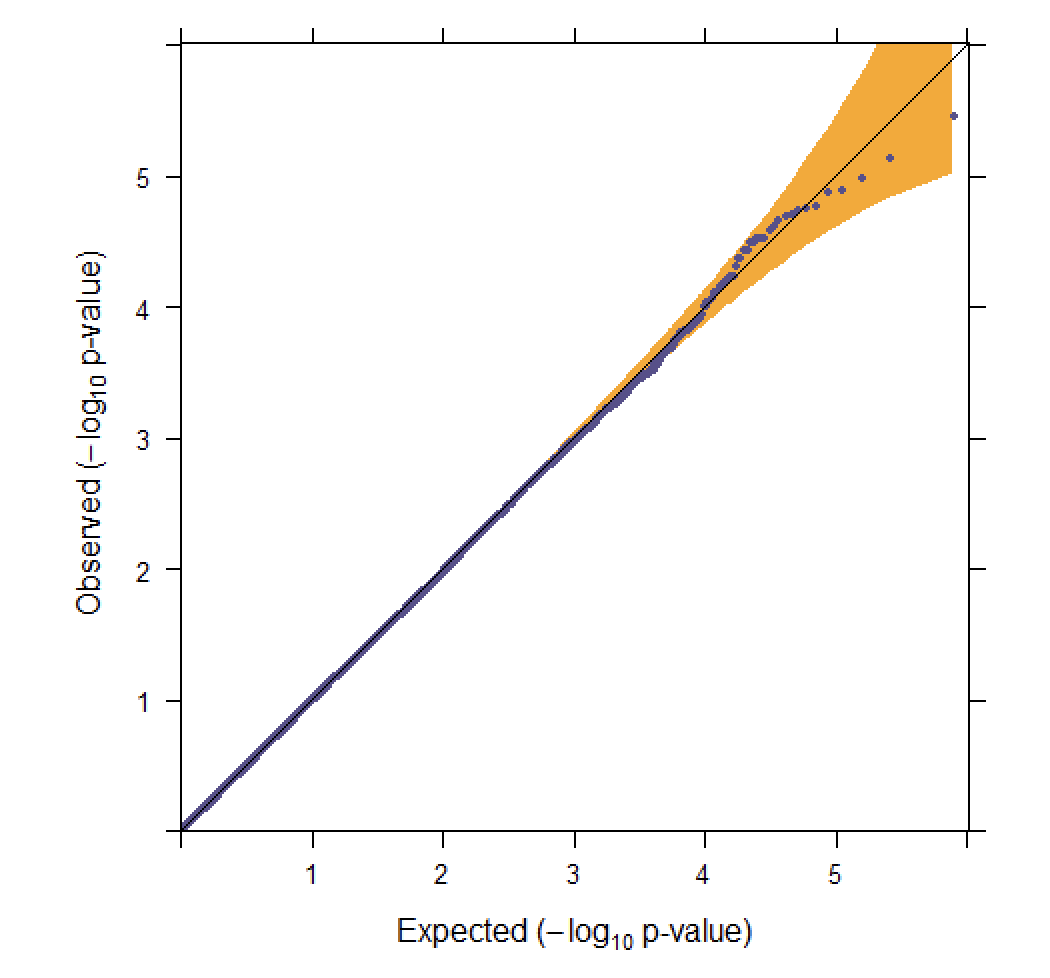


**Figure S4**. Q-Q plot: trimester 2 infections (Generation R).

**Figure S5**. Manhattan plot: trimester 3 infections (Generation R). The purple line indicates the suggestive significance threshold, and the red line indicates the Bonferroni adjusted significance threshold.


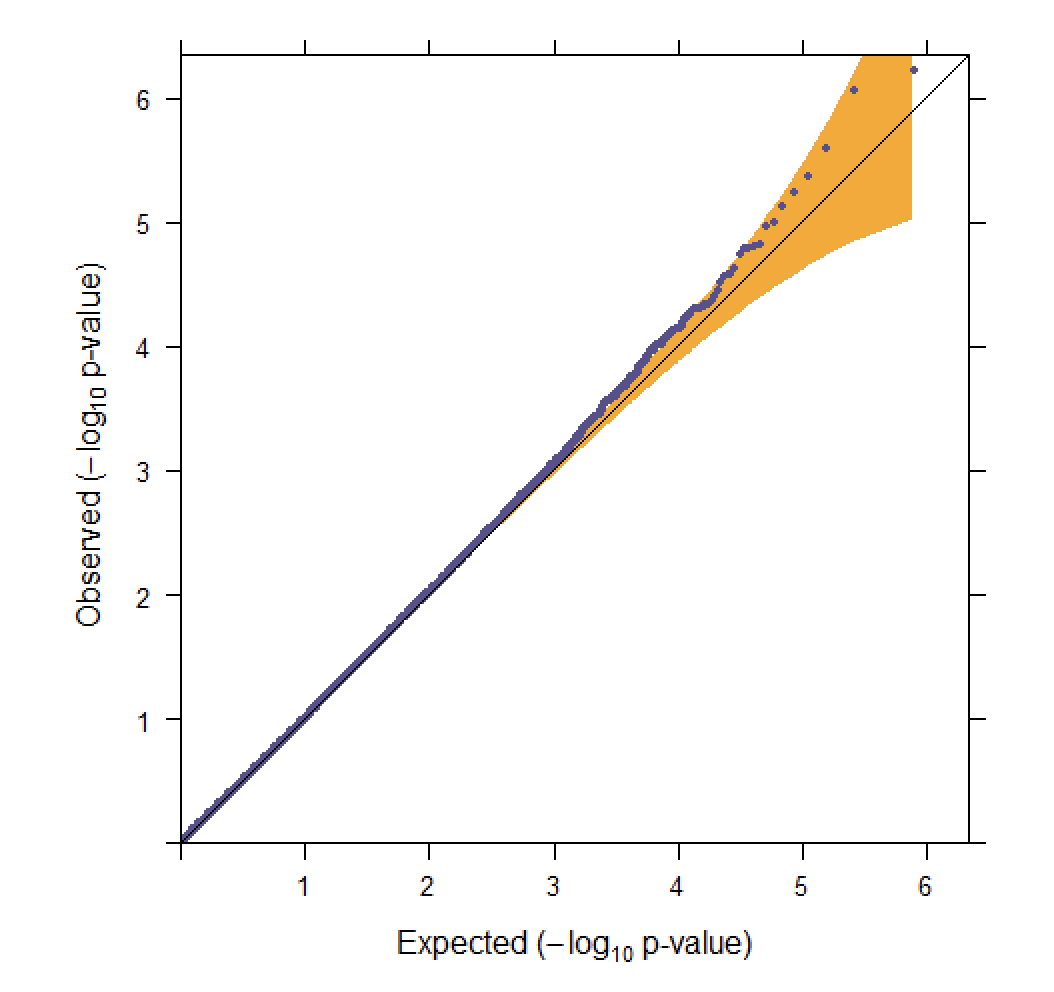


**Figure S6**. Q-Q plot: trimester 3 infections (Generation R).

**Figure S7**. Q-Q plots for total prenatal infection EWAS without adjusting for cell type proportions in the discovery cohort.


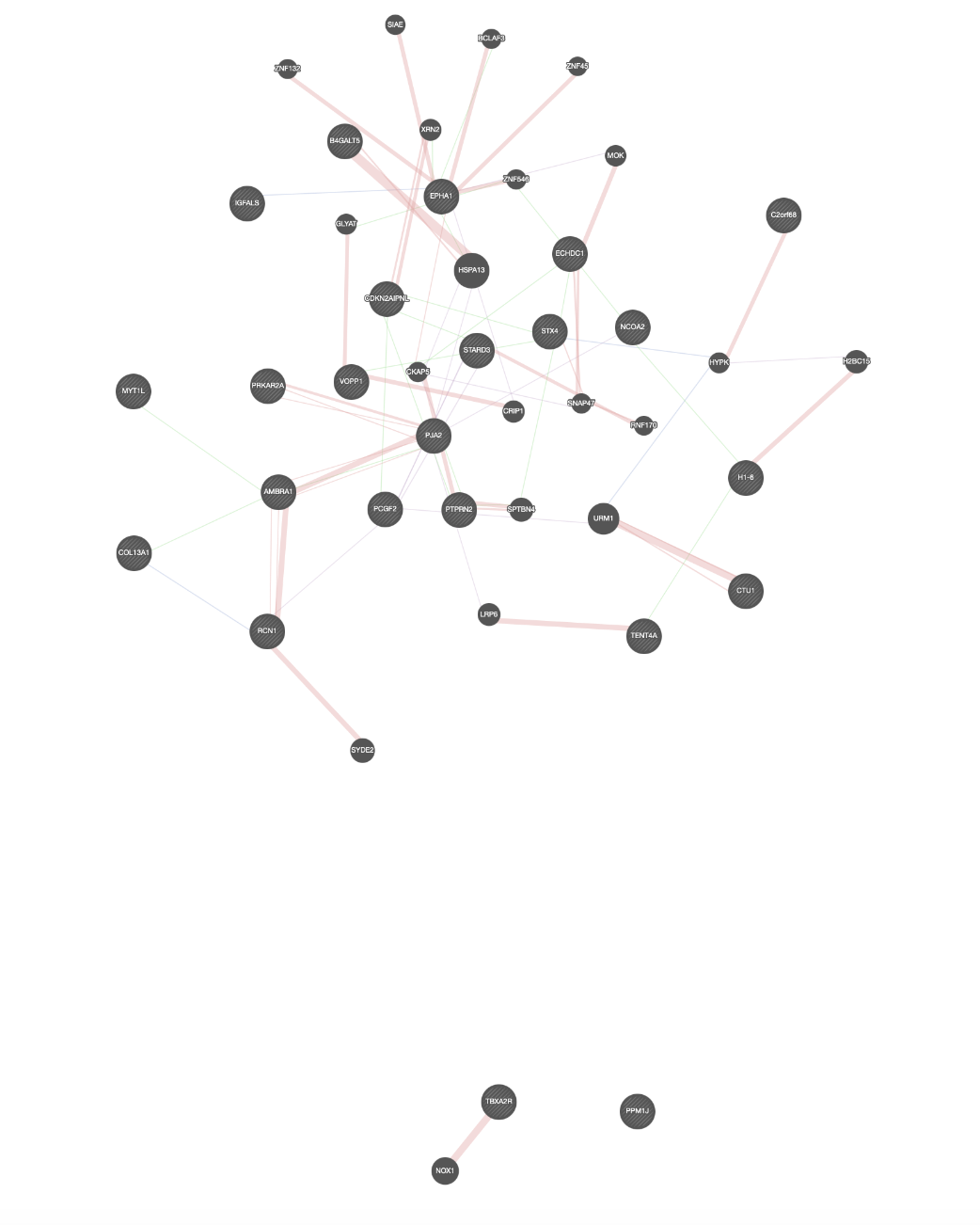


**Figure S8**. Genemania results for EWAS nearest genes for the total infection sum score.


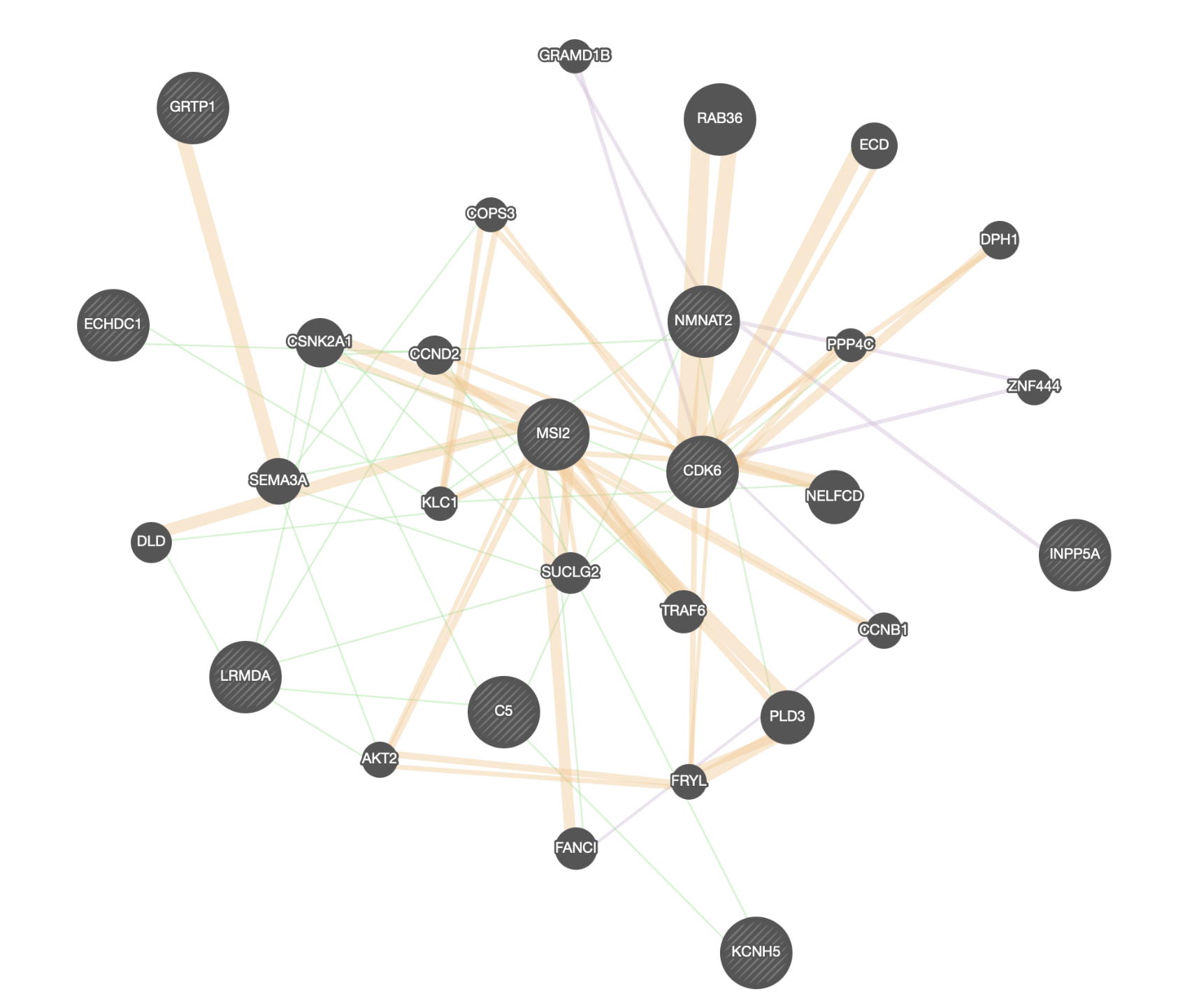


**Figure S9**. Genemania results for EWAS nearest genes for the trimester 1 infection sum score.


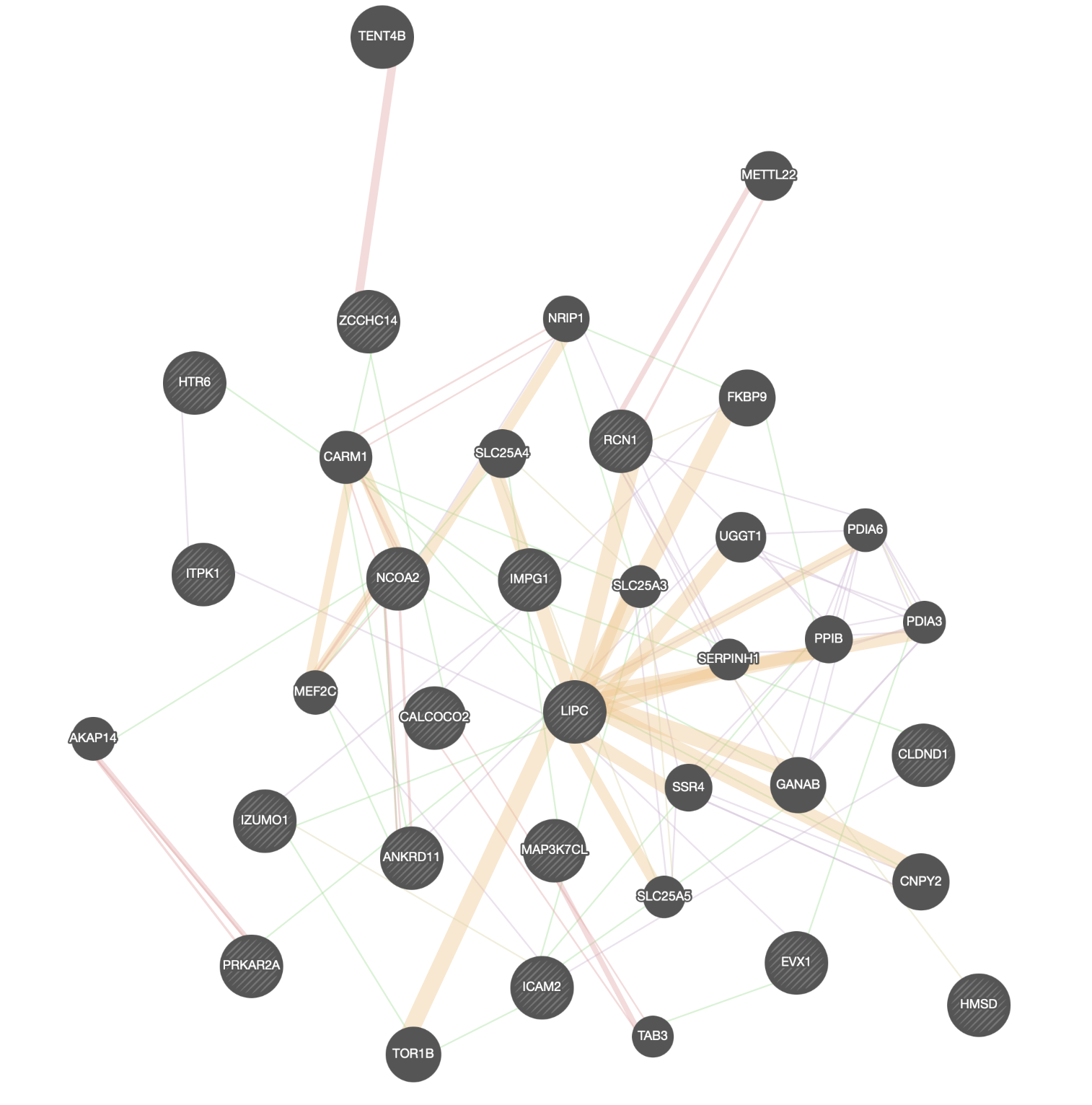


**Figure S10**. Genemania results for EWAS nearest genes for the trimester 2 infection sum score.


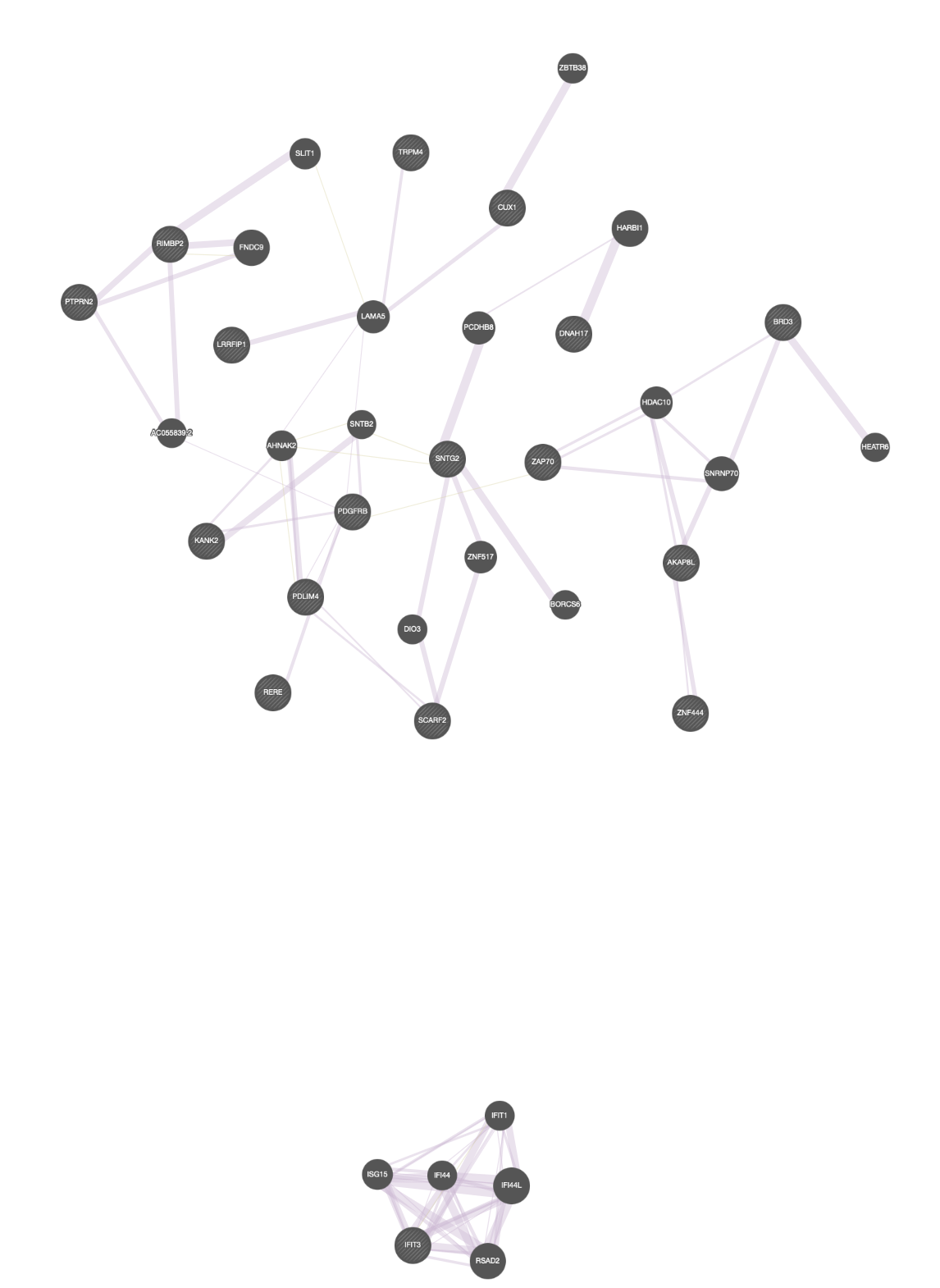


**Figure S11**. Genemania results for EWAS nearest genes for the trimester 3 infection sum score.


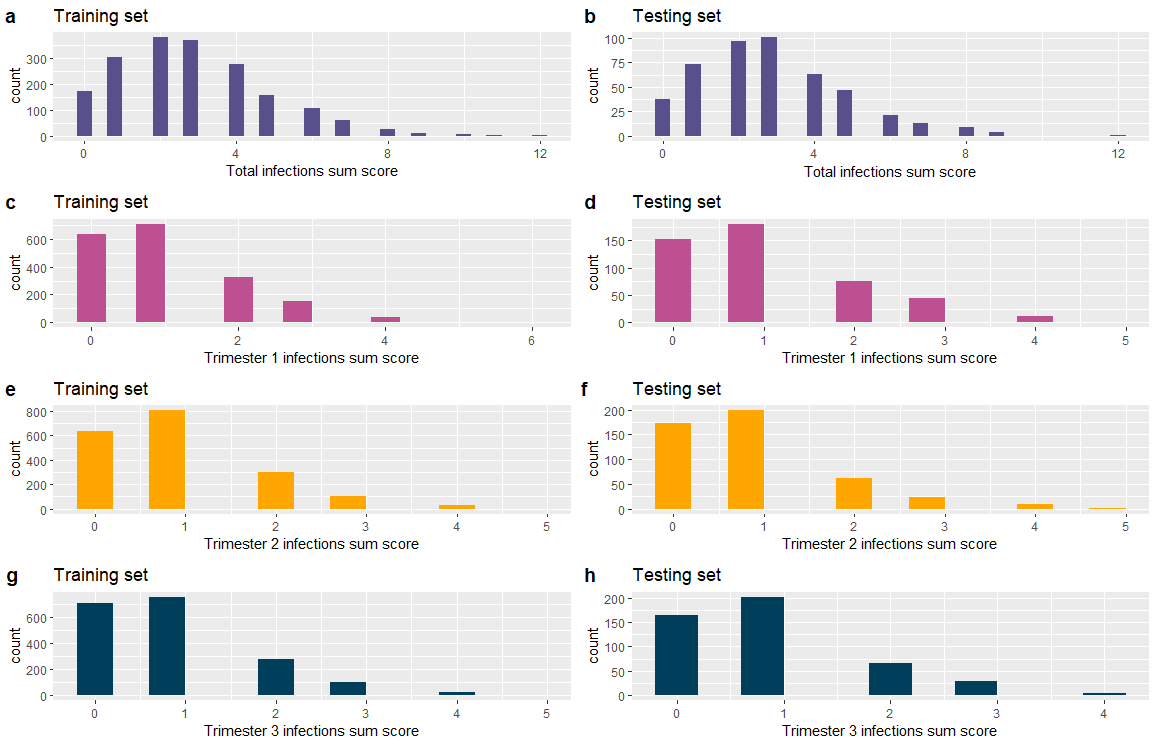


**Figure S12**. Distribution of infection sum scores in the Generation R train and test set.

**Figure S13**. Distribution of all methylation profile scores in the Generation R test set.

**Figure S14**. Distribution of all methylation profile scores in the ALSPAC data set.

**Figure S15**. Diagnostics for MPS – total infection in the Generation R test set and ALSPAC data set. Of note, both the MPS score and prenatal infection sum score are standardized.


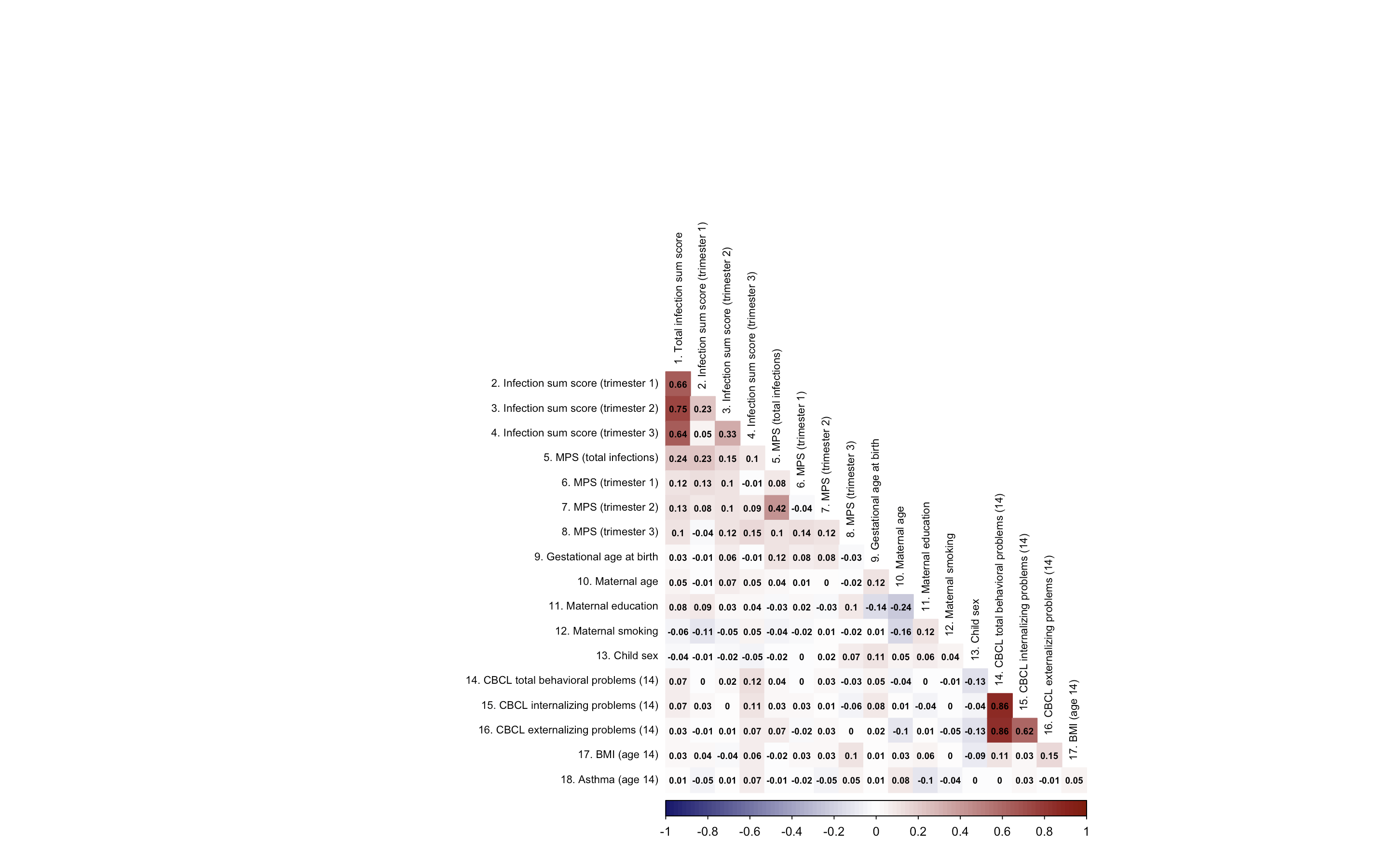


**Figure S16**. Correlation plot Generation R test set.


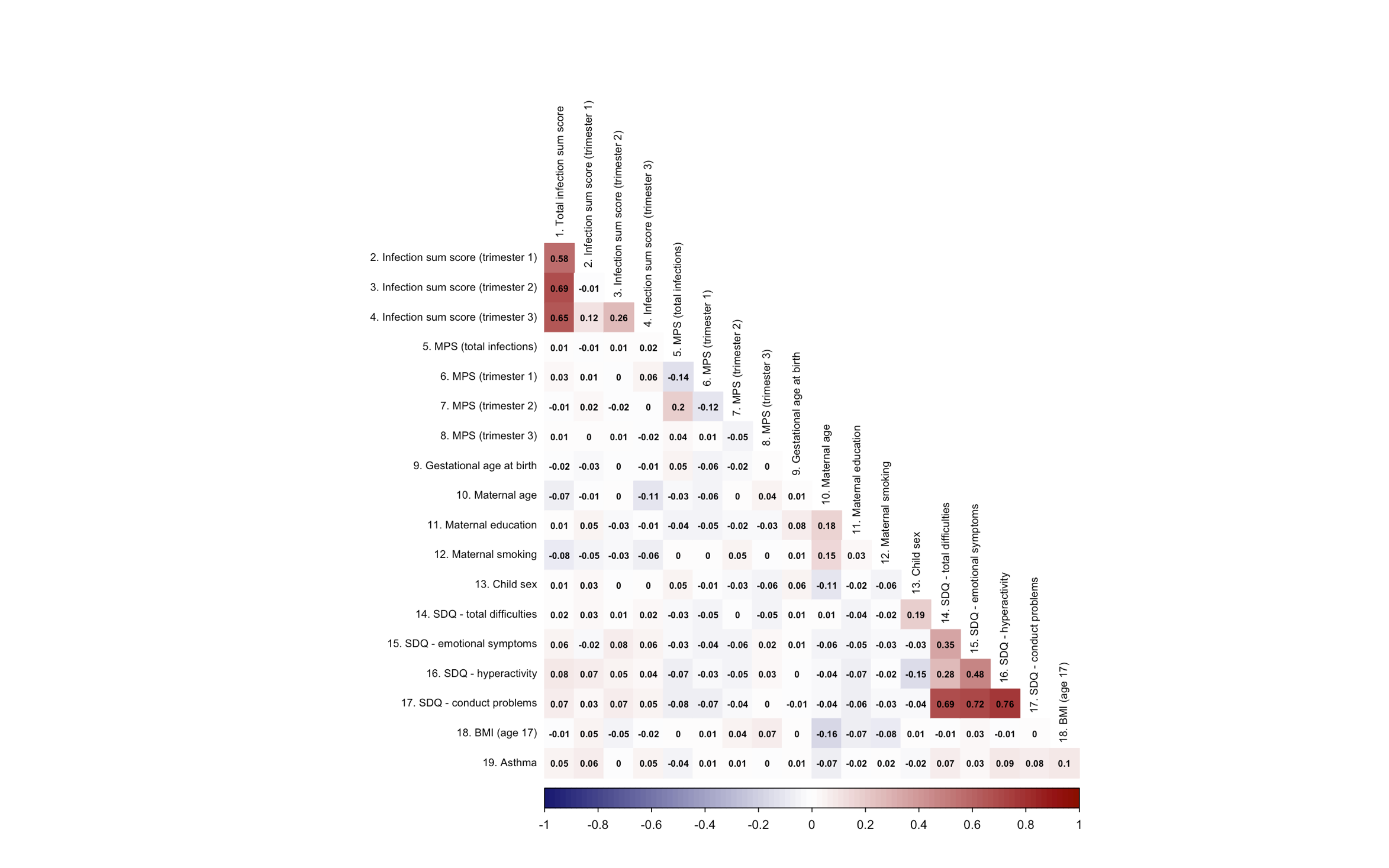


**Figure S17**. Correlation plot ALSPAC data set.

## **Supplementary tables**

**Table S1**. Trimester-based infection sum scores and DNA methylation at birth

| CpG | Beta | SE | P-value | Chr | Pos | Relation to island | Nearest gene |
| --- | --- | --- | --- | --- | --- | --- | --- |
| *Trimester 1 infection sum score* | | | | | | | |
| cg12963656 | 0.003 | 0.001 | 6.869E-07 | chr3 | 101659687 | OpenSea | *LOC152225* |
| cg22430861 | 0.003 | 0.001 | 8.045E-07 | chr17 | 55584079 | OpenSea | *MSI2* |
| cg05544413 | 0.004 | 0.0001 | 8.818E-07 | chr6 | 127663627 | N_Shore | *ECHDC1* |
| cg16698470 | -0.003 | 0.001 | 1.429E-06 | chr11 | 38413823 | OpenSea | *-* |
| cg01253508 | -0.002 | 3.692E-04 | 4.025E-06 | chr5 | 173738923 | Island | *-* |
| cg19532454* | -0.001 | 3.303E-04 | 6.328E-06 | chr3 | 46876354 | S_Shore | *PRSS42* |
| cg23993890 | -0.001 | 3.319E-04 | 7.023E-06 | chr1 | 183221358 | OpenSea | *NMNAT2* |
| cg12995004* | -0.002 | 3.695E-04 | 8.3691E-06 | chr5 | 17395952 | OpenSea | *-* |
| cg02836764 | -0.001 | 3.048E-04 | 8.553E-06 | chr10 | 134499364 | OpenSea | *INPP5A* |
| cg02987635 | -0.002 | 3.642E-04 | 1.005E-05 | chr10 | 77794658 | OpenSea | *C10orf11* |
| cg23962849 | 0.001 | 3.225E-04 | 1.685E-05 | chr19 | 42776879 | N_Shore | *-* |
| cg04725669 | -0.001 | 2.437E-04 | 1.958E-05 | chr7 | 92465270 | Island | *CDK6* |
| cg01843018 | -0.002 | 5.458E-04 | 2.442E-05 | chr9 | 123813144 | OpenSea | *C5* |
| cg11886260 | -0.002 | 4.941E-04 | 2.723E-05 | chr14 | 63451319 | OpenSea | *KCNH5* |
| cg06037095* | -0.003 | 6.697E-04 | 2.811E-05 | chr3 | 166444106 | OpenSea | *-* |
| cg11730722 | 0.001 | 2.901E-04 | 3.292E-05 | chr10 | 102985514 | Island | *-* |
| cg14518261 | 0.002 | 4.313E-04 | 3.336E-05 | chr13 | 113997250 | OpenSea | *GRTP1* |
| cg11982176 | 0.002 | 4.060E-04 | 3.467E-05 | chr13 | 114054286 | N_Shore | *-* |
| cg00702872* | -0.002 | 4.493E-04 | 3.563E-05 | chr6 | 3524580 | OpenSea | *-* |
| cg23585420 | -0.003 | 6.293E-04 | 4.672E-05 | chr14 | 104668772 | OpenSea | *-* |
| *Trimester 2 infection sum score* | | | | | | | |
| cg02854164 | -0.00245 | 0.0001 | 3.450E-06 | chr16 | 87474977 | OpenSea | *ZCCHC14* |
| cg09130190 | 0.001633 | 3.670E-04 | 8.570E-06 | chr11 | 32115439 | S_Shelf | *RCN1* |
| cg03987884 | 0.002775 | 0.001 | 8.690E-06 | chr18 | 61616862 | OpenSea | *HMSD* |
| cg16691158 | -0.00219 | 4.920E-04 | 8.720E-06 | chr5 | 139140550 | S_Shore | *-* |
| cg27258283 | 0.001479 | 3.370E-04 | 1.170E-05 | chr16 | 89337103 | S_Shelf | *ANKRD11* |
| cg16060163 | 0.001904 | 4.410E-04 | 1.540E-05 | chr17 | 80454814 | Island | *-* |
| cg11254196* | 0.001992 | 4.660E-04 | 1.890E-05 | chr7 | 48109763 | OpenSea | *-* |
| cg26289665 | -0.00152 | 3.550E-04 | 1.920E-05 | chr21 | 30547239 | OpenSea | *C21orf7* |
| cg26068141 | 0.000353 | 8.290E-05 | 2.090E-05 | chr8 | 71315032 | Island | *NCOA2* |
| cg12284075* | -0.00143 | 3.360E-04 | 2.090E-05 | chr15 | 74541993 | S_Shelf | *CCDC33* |
| cg23667178 | -0.00083 | 1.970E-04 | 2.400E-05 | chr8 | 1151560 | S_Shore | *-* |
| cg20968717 | -0.00136 | 3.240E-04 | 2.710E-05 | chr15 | 58758468 | OpenSea | *LIPC* |
| cg16732175 | 0.001453 | 3.460E-04 | 2.740E-05 | chr19 | 49250180 | N_Shelf | *IZUMO1* |
| cg01304814 | -0.00068 | 1.620E-04 | 2.870E-05 | chr3 | 48885189 | Island | *PRKAR2A* |
| cg16047144 | -0.00079 | 1.900E-04 | 2.930E-05 | chr17 | 62097953 | OpenSea | *ICAM2* |
| cg06033660* | -0.00261 | 0.001 | 2.950E-05 | chr1 | 19992985 | S_Shore | *HTR6* |
| cg15979035 | 0.002163 | 0.001 | 3.040E-05 | chr14 | 93698870 | OpenSea | *-* |
| cg27100227 | 0.002434 | 0.001 | 3.250E-05 | chr7 | 27281687 | N_Shore | *EVX1* |
| cg05379891 | 0.001093 | 2.650E-04 | 3.750E-05 | chr3 | 98243147 | S_Shore | *CLDND1* |
| cg04643552 | -0.00128 | 3.120E-04 | 3.950E-05 | chr15 | 48191214 | OpenSea | *-* |
| cg27530239 | 0.002869 | 0.001 | 3.970E-05 | chr17 | 46909074 | S_Shore | *CALCOCO2* |
| cg11170479* | 0.001387 | 3.400E-04 | 4.540E-05 | chr14 | 93517493 | OpenSea | *ITPK1* |
| cg16622651 | -0.00203 | 0.001 | 4.950E-05 | chr6 | 76724431 | OpenSea | *IMPG1* |
| cg21218820 | -0.00188 | 4.630E-04 | 4.960E-05 | chr14 | 97091520 | OpenSea | *-* |
| *Trimester 3 infection sum score* | | | | | | | |
| cg02355411 | -0.00216 | 4.310E-04 | 5.43518E-07 | chr19 | 49700427 | S_Shore | *TRPM4* |
| cg05772104 | -0.00473 | 0.001 | 1.049E-06 | chr2 | 98340425 | Island | *ZAP70* |
| cg23496331* | 0.001796 | 3.830E-04 | 2.670E-06 | chr5 | 149512405 | Island | *PDGFRB* |
| cg20756891 | 0.001979 | 4.290E-04 | 4.016E-06 | chr5 | 170743951 | Island | *-* |
| cg27425784 | -0.00449 | 0.001 | 6.824E-06 | chr2 | 98340511 | Island | *ZAP70* |
| cg24919344 | -0.00186 | 4.160E-04 | 7.525E-06 | chr5 | 24208918 | OpenSea | *-* |
| cg24539060 | -0.00082 | 1.840E-04 | 9.249E-06 | chr17 | 76570762 | OpenSea | *DNAH17* |
| cg15016481 | -0.00144 | 3.250E-04 | 9.544E-06 | chr2 | 238649819 | OpenSea | *LRRFIP1* |
| cg26602975 | -0.00138 | 3.190E-04 | 1.410E-05 | chr11 | 113181884 | N_Shelf | *-* |
| cg23993005 | 0.004597 | 0.001 | 1.501E-05 | chr10 | 91091209 | OpenSea | *IFIT3* |
| cg20385216 | -0.00274 | 0.001 | 1.556E-05 | chr5 | 131607149 | Island | *PDLIM4* |
| cg15702838 | 0.000715 | 1.670E-04 | 1.811E-05 | chr19 | 11289211 | Island | *KANK2* |
| cg26797894* | -0.00188 | 4.440E-04 | 2.219E-05 | chr2 | 211043474 | OpenSea | *-* |
| cg05845765 | 0.002882 | 0.001 | 2.464E-05 | chr7 | 101880148 | N_Shelf | *CUX1* |
| cg01364935 | -0.0021 | 0.001 | 2.821E-05 | chr16 | 73185687 | OpenSea | *-* |
| cg19334350 | -0.00558 | 0.001 | 3.066E-05 | chr7 | 157406737 | S_Shore | *PTPRN2* |
| cg25703213 | 0.002085 | 0.001 | 3.222E-05 | chr12 | 130909656 | S_Shore | *RIMBP2* |
| cg08684551 | -0.00063 | 1.530E-04 | 3.878E-05 | chr19 | 56652453 | Island | *ZNF444* |
| cg05129802 | -0.00135 | 3.300E-04 | 4.057E-05 | chr16 | 85517587 | N_Shore | *-* |
| cg13315744 | 0.001941 | 4.730E-04 | 4.087E-05 | chr2 | 1287787 | S_Shore | *SNTG2* |
| cg22816534 | -0.00036 | 8.890E-05 | 4.481E-05 | chr1 | 8877774 | Island | *RERE* |
| cg25569582 | 0.000785 | 1.9200E-04 | 4.518E-05 | chr9 | 136933818 | OpenSea | *BRD3* |
| cg23299919* | -0.00854 | 0.002 | 4.637E-05 | chr7 | 157406096 | Island | *PTPRN2* |
| cg19895164 | 0.000948 | 2.330E-04 | 4.720E-05 | chr22 | 20791024 | Island | *SCARF2* |
| cg07036524 | -0.00301 | 0.001 | 4.866E-05 | chr18 | 77557781 | Island | *-* |
| cg24773324 | 0.000649 | 1.600E-04 | 4.999E-05 | chr19 | 15529844 | Island | *AKAP8L* |
| *CpG contains snp’s | | | | | | | |

**Table S2**. Total prenatal infection EWAS without adjusting for cell type proportions (model 1)

| CpG | Beta | SE | P-value | Chromosome | Position | Relation to island | Nearest gene |
| --- | --- | --- | --- | --- | --- | --- | --- |
| cg16060163 | 0.002 | 4.520E-04 | 6.520E-06 | chr17 | 80454814 | Island | - |
| cg25376660 | -0.001 | 3.320E-04 | 1.100E-05 | chr2 | 1942893 | S_Shore | *MYT1L* |
| cg27005246 | 0.001 | 2.030E-04 | 1.270E-05 | chr16 | 31044731 | Island | *STX4* |
| cg00264346 | 0.001 | 1.950E-04 | 1.500E-05 | chr17 | 37793307 | Island | *STARD3* |
| cg27466042 | 0.001 | 3.430E-04 | 1.600E-05 | chr17 | 36896370 | OpenSea | *PCGF2* |
| cg00093220 | 0.001 | 2.610E-04 | 1.610E-05 | chr1 | 113258217 | S_Shore | *PPM1J* |
| cg26718621 | 0.001 | 1.390E-04 | 1.640E-05 | chr5 | 133747703 | Island | *CDKN2AIPNL* |
| cg18756291 | -0.001 | 1.310E-04 | 2.240E-05 | chr2 | 85839143 | Island | *C2orf68* |
| cg02296145 | -0.001 | 1.570E-04 | 2.440E-05 | chr16 | 1840711 | Island | *IGFALS* |
| cg01304814 | -0.001 | 1.640E-04 | 2.540E-05 | chr3 | 48885189 | Island | *PRKAR2A* |
| cg27426220 | 0.001 | 2.420E-04 | 2.570E-05 | chr7 | 143106126 | OpenSea | *EPHA1* |
| cg04478698 | -0.001 | 3.250E-04 | 2.800E-05 | chr4 | 26033992 | S_Shelf | - |
| cg10600568 | 0.001 | 2.790E-04 | 3.570E-05 | chr19 | 51611605 | Island | *CTU1* |
| cg19334350 | -0.006 | 0,001 | 4.590E-05 | chr7 | 157406737 | S_Shore | *PTPRN2* |
| cg17050810 | 0.001 | 3.060E-04 | 4.660E-05 | chr6 | 26108174 | S_Shelf | *HIST1H1T* |
| cg26068141 | 3.390E-04 | 8,320E-05 | 4.730E-05 | chr8 | 71315032 | Island | *NCOA2* |
| cg25839724 | 0.003 | 0,001 | 4.820E-05 | chr1 | 203552475 | OpenSea | - |

**Table S3**. Functional analyses of suggestive hits

| CpG | mQTL | Cis/trans | eQTM | CpG gene | TC gene |
| --- | --- | --- | --- | --- | --- |
| *Total infection sum score* | | | | | |
| cg17050810 | - | - | no | - | - |
| cg16060163 | - | - | no | - | - |
| cg27466042 | - | - | no | - | - |
| cg04478698 | Yes, 50 hits | 1 | no | - | - |
| cg12058372 | Yes, 206 hits | 1 | no | - | - |
| cg25376660 | - | - | no | - | - |
| cg00702872 | Yes, 458 hits | 1 | yes | - | *SLC22A23* |
| cg09130190 | - | - | no | - | - |
| cg00093220 | - | - | no | - | - |
| cg00264346 | - | - | no | - | - |
| cg26718621 | - | - | no | - | - |
| cg03300596 | - | - | no | - | - |
| cg27426220 | - | - | no | - | - |
| cg02296145 | - | - | no | - | - |
| cg23962849 | - | - | no | - | - |
| cg18756291 | - | - | no | - | - |
| cg01304814 | Yes, 1249 hits | 1 and 0 (30 hits show 0, rest shows 1) | yes | *PRKAR2A* | *QRICH1* |
| cg16371518 | - | - | no | - | - |
| cg19334350 | Yes, 30 hits | 1 | no | - | - |
| cg10600568 | - | - | no | - | - |
| cg25839724 | Yes, 236 hits | 1 | no | - | - |
| cg27005246 | - | - | no | - | - |
| cg05544413 | Yes, 704 hits | 1 | no | - | - |
| cg16691158 | Yes, 34 hits | 1 | no | - | - |
| cg13904667 | - | - | no | - | - |
| cg08337633 | Yes, 677 hits | 1 | yes | *VOPP1* | *VOPP1* |
| cg08795170 | - | - | no | - | - |
| cg26068141 | - | - | no | - | - |
| cg13680337 | Yes, 127 hits | 1 | no | - | - |
| cg14383355 | - | - | no | - | - |
| cg18738857 | Yes, 81 hits | 1 | no | - | - |
| cg19445322 | Yes, 134 hits | 1 | no | - | - |
| cg08725904 | Yes, 59 hits | 1 | no | - | - |
| *Trimester 1 infection sum score* | | | | | |
| cg12963656 | Yes, 750 hits | 1 | no | - | - |
| cg22430861 | Yes, 71 hits | 1 | no | - | - |
| cg05544413 | Yes, 704 hits | 1 | no | - | - |
| cg16698470 | - | - | no | - | - |
| cg01253508 | Yes, 10 hits | 1 | no | - | - |
| cg19532454 | Yes, 26 hits | 0 | no | - | - |
| cg23993890 | - | - | no | - | - |
| cg12995004 | Yes, 26 hits | 0 | no | - | - |
| cg02836764 | - | - | no | - | - |
| cg02987635 | - | - | no | - | - |
| cg23962849 | - | - | no | - | - |
| cg04725669 | - | - | no | - | - |
| cg01843018 | - | - | no | - | - |
| cg11886260 | - | - | no | - | - |
| cg06037095 | Yes, 2 hits | 1 | no | - | - |
| cg11730722 | - | - | no | - | - |
| cg14518261 | Yes, 28 hits | 1 | no | - | - |
| cg11982176 | Yes, 134 hits | 1 | no | - | - |
| cg00702872 | Yes, 458 hits | 1 | yes | - | *SLC22A23* |
| cg23585420 | Yes, 254 hits | 1 | no | - | - |
| *Trimester 2* *infection sum score* | | | | | |
| cg02854164 | - | - | no | - | - |
| cg09130190 | - | - | no | - | - |
| cg03987884 | Yes, 529 hits | 1 | yes | *HMSD* | *SERPINB8* |
| cg16691158 | Yes, 34 hits | 1 | no | - | - |
| cg27258283 | Yes, 1 hit | 1 | no | - | - |
| cg16060163 | - | - | no | - | - |
| cg11254196 | - | - | no | - | - |
| cg26289665 | - | - | no | - | - |
| cg26068141 | - | - | no | - | - |
| cg12284075 | - | - | no | - | - |
| cg23667178 | - | - | no | - | - |
| cg20968717 | Yes, 64 hits | 1 | no | - | - |
| cg16732175 | - | - | no | - | - |
| cg01304814 | Yes, 1249 hits | 0 and 1 (of which 30 hits are 0) | yes | *PRKAR2A* | *QRICH1* |
| cg16047144 | - | - | no | - | - |
| cg06033660 | Yes, 3 hits | 1 | no | - | - |
| cg15979035 | Yes, 517 hits | 1 | no | - | - |
| cg27100227 | - | - | no | - | - |
| cg05379891 | - | - | no | - | - |
| cg04643552 | - | - | no | - | - |
| cg27530239 | Yes, 84 hits | 1 | no | - | - |
| cg11170479 | - | - | no | - | - |
| cg16622651 | - | - | no | - | - |
| cg21218820 | Yes, 162 hits | 1 | no | - | - |
| *Trimester 3 infection sum score* | | | | | |
| cg02355411 | Yes, 5 hits | 1 | no | - | - |
| cg05772104 | Yes, 1057 hits | 1 | no | - | - |
| cg23496331 | Yes, 1 hit | 1 | no | - | - |
| cg20756891 | - | - | no | - | - |
| cg27425784 | Yes, 1002 hits | 1 | no | - | - |
| cg24919344 | Yes, 1239 hits | 1 | no | - | - |
| cg24539060 | Yes, 1 hit | 1 | no | - | - |
| cg15016481 | Yes, 139 hits | 1 | no | - | - |
| cg26602975 | - | - | no | - | - |
| cg23993005 | - | - | no | - | - |
| cg20385216 | Yes, 1170 hits | 0 and 1 (of which 384 are 0) | no | - | - |
| cg15702838 | - | - | no | - | - |
| cg26797894 | - | - | no | - | - |
| cg05845765 | Yes, 454 hits | 0 | no | - | - |
| cg01364935 | Yes, 122 hits | 1 | no | - | - |
| cg19334350 | Yes, 30 hits | 1 | no | - | - |
| cg25703213 | Yes, 23 hits | 1 | no | - | - |
| cg08684551 | - | - | no | - | - |
| cg05129802 | Yes, 39 hits | 0 | no | - | - |
| cg13315744 | Yes, 30 hits | 1 | no | - | - |
| cg22816534 | - | - | no | - | - |
| cg25569582 | Yes, 1 hit | 1 | no | - | - |
| cg23299919 | Yes, 2 hits | 0 | no | - | - |
| cg19895164 | - | - | no | - | - |
| cg07036524 | Yes, 27 hits | 1 | no | - | - |
| cg24773324 | - | - | no | - | - |
| cg25569582 | Yes, 5 hits | 1 | no | - | - |
| 1 = cis, 0 = trans | | | | | |

**Table S4**. GO analysis results of suggestive hits (top 5 pathways per category with at least 1 gene)

| Ontology | Term | N | P.DE | FDR |
| --- | --- | --- | --- | --- |
| *Total infection sum score* | | | | |
| MF | protein kinase A catalytic subunit binding | 13 | 3.100E-04 | 1 |
| MF | protein kinase A binding | 47 | 0.003 | 1 |
| MF | UDP-galactose:glucosylceramide beta-1.4-galactosyltransferase activity | 3 | 0.003 | 1 |
| MF | sphingomyelin phosphodiesterase activator activity | 2 | 0.004 | 1 |
| CC | phagocytic vesicle | 130 | 0.001 | 1 |
| CC | storage vacuole | 2 | 0.003 | 1 |
| CC | Tapasin-ERp57 complex | 2 | 0.004 | 1 |
| BP | chromosome condensation | 43 | 0.001 | 1 |
| BP | positive regulation of eosinophil degranulation | 2 | 0.003 | 1 |
| BP | positive regulation of eosinophil activation | 2 | 0.003 | 1 |
| BP | regulation of eosinophil degranulation | 3 | 0.004 | 1 |
| *Trimester 1 infection sum score* | | | | |
| MF | protein ADP-ribosyltransferase-substrate adaptor activity | 3 | 0.001 | 1 |
| MF | FBXO family protein binding | 2 | 0.002 | 1 |
| MF | nicotinamide-nucleotide adenylyltransferase activity | 3 | 0.002 | 1 |
| MF | nicotinate-nucleotide adenylyltransferase activity | 4 | 0.004 | 1 |
| MF | inositol-1.3.4.5-tetrakisphosphate 5-phosphatase activity | 5 | 0.005 | 1 |
| CC | membrane attack complex | 7 | 0.001 | 1 |
| BP | regulation of G2/M transition of mitotic cell cycle | 97 | 0.002 | 1 |
| BP | regulation of cell cycle G2/M phase transition | 107 | 0.002 | 1 |
| BP | negative regulation of macrophage chemotaxis | 7 | 0.003 | 1 |
| BP | G2/M transition of mitotic cell cycle | 132 | 0.003 | 1 |
| BP | cell cycle G2/M phase transition | 146 | 0.004 | 1 |
| *Trimester 2 infection sum score* | | | | |
| MF | inositol tetrakisphosphate 6-kinase activity | 2 | 0.003 | 1 |
| MF | inositol tetrakisphosphate kinase activity | 5 | 0.007 | 1 |
| MF | inositol trisphosphate kinase activity | 5 | 0.007 | 1 |
| MF | histamine receptor activity | 6 | 0.007 | 1 |
| MF | 1-acyl-2-lysophosphatidylserine acylhydrolase activity | 10 | 0.008 | 1 |
| CC | protein complex involved in cell-cell adhesion | 5 | 0.005 | 1 |
| CC | cAMP-dependent protein kinase complex | 5 | 0.005 | 1 |
| CC | interphotoreceptor matrix | 6 | 0.006 | 1 |
| BP | inositol phosphorylation | 2 | 0.003 | 1 |
| BP | cellular response to Thyroglobulin triiodothyronine | 3 | 0.004 | 1 |
| BP | chylomicron remnant clearance | 6 | 0.004 | 1 |
| BP | triglyceride-rich lipoprotein particle clearance | 6 | 0.004 | 1 |
| BP | spinal cord interneuron axon guidance | 3 | 0.006 | 1 |
| *Trimester 3 infection sum score* | | | | |
| MF | lysine-acetylated histone binding | 22 | 4.340E-04 | 1 |
| MF | acetylation-dependent protein binding | 22 | 4.340E-04 | 1 |
| MF | platelet-derived growth factor beta-receptor activity | 2 | 0.001 | 1 |
| MF | platelet activating factor receptor activity | 3 | 0.001 | 1 |
| CC | early endosome lumen | 3 | 0.004 | 1 |
| BP | lung growth | 7 | 0.003 | 1 |
| BP | insulin secretion involved in cellular response to glucose stimulus | 57 | 0.004 | 1 |
| BP | positive regulation of hepatic stellate cell activation | 5 | 0.004 | 1 |
| BP | negative regulation of vitamin D receptor signaling pathway | 2 | 0.005 | 1 |
| BP | beta selection | 3 | 0.005 | 1 |
| MF = molecular function, BP = biological processes, CC = cellular components | | | | |

**Table S5**. KEGG analysis results of suggestive hits

| Description | N | P.DE | FDR |
| --- | --- | --- | --- |
| *Total infection sum score* | | | |
| Sulfur relay system | 8 | 0.007 | 1 |
| SNARE interactions in vesicular transport | 32 | 0.042 | 1 |
| Propanoate metabolism | 32 | 0.045 | 1 |
| *Trimester 1 infection sum score* | | | |
| Nicotinate and nicotinamide metabolism | 35 | 0.016 | 1 |
| Propanoate metabolism | 32 | 0.017 | 1 |
| Staphylococcus aureus infection | 87 | 0.031 | 1 |
| Pertussis | 75 | 0.031 | 1 |
| Complement and coagulation cascades | 82 | 0.033 | 1 |
| Metabolic pathways | 1443 | 0.034 | 1 |
| Systemic lupus erythematosus | 113 | 0.041 | 1 |
| mRNA surveillance pathway | 84 | 0.047 | 1 |
| Inositol phosphate metabolism | 69 | 0.047 | 1 |
| p53 signaling pathway | 74 | 0.049 | 1 |
| *Trimester 2 infection sum score* | | | |
| Cholesterol metabolism | 50 | 0.046 | 1 |
| *Trimester 3 infection sum score* | | | |
| Primary immunodeficiency | 32 | 0.038 | 1 |

**Table S6**. Results enrichment analysis with C7 immunological signature gene sets.

| Term | N | P.DE | FDR | Significant genes in set | |
| --- | --- | --- | --- | --- | --- |
| *Total infections* | | | | |  |
| GSE14769_UNSTIM_VS_360MIN_LPS_BMDM_DN | 197 | 8.552e-06* | 0.041** | AMBRA1,TAPBP,VOPP1,CTU1,PJA2 | |
| GSE4590_PRE_BCELL_VS_LARGE_PRE_BCELL_UP | 154 | 0.001* | 0.706 | RCN1,PCGF2,VOPP1 | |
| GSE18791_CTRL_VS_NEWCASTLE_VIRUS_DC_4H_DN | 181 | 0.002* | 0.706 | TENT4A,H1-6,B4GALT5 | |
| GSE19888_CTRL_VS_TCELL_MEMBRANES_ACT_MAST_CELL_PRETREAT_A3R_INH_DN | 193 | 0.002* | 0.706 | TENT4A,TAPBP,B4GALT5 | |
| GSE15330_LYMPHOID_MULTIPOTENT_VS_MEGAKARYOCYTE_ERYTHROID_PROGENITOR_IKAROS_KO_DN | 171 | 0.002* | 0.706 | PPM1J,PRKAR2A,PCGF2 | |
| GSE2935_UV_INACTIVATED_VS_LIVE_SENDAI_VIRUS_INF_MACROPHAGE_DN | 174 | 0.002* | 0.706 | PRKAR2A,PCGF2,CDKN2AIPNL | |
| GSE11961_MARGINAL_ZONE_BCELL_VS_PLASMA_CELL_DAY7_DN | 192 | 0.002* | 0.706 | RCN1,VOPP1,CTU1 | |
| GSE411_WT_VS_SOCS3_KO_MACROPHAGE_IL6_STIM_100MIN_DN | 194 | 0.002* | 0.706 | STARD3,C2orf68,CTU1 | |
| GSE3565_CTRL_VS_LPS_INJECTED_SPLENOCYTES_UP | 171 | 0.002* | 0.706 | PRKAR2A,PCGF2,B4GALT5 | |
| GSE3565_CTRL_VS_LPS_INJECTED_DUSP1_KO_SPLENOCYTES_UP | 176 | 0.002* | 0.706 | PRKAR2A,PCGF2,B4GALT5 | |
| GSE8921_UNSTIM_VS_TLR1_2_STIM_MONOCYTE_6H_DN | 194 | 0.003* | 0.706 | PPM1J,TAPBP,B4GALT5 | |
| GSE27786_BCELL_VS_ERYTHROBLAST_UP | 195 | 0.003* | 0.706 | NCOA2,C2orf68,TAPBP | |
| GSE26669_CD4_VS_CD8_TCELL_IN_MLR_DN | 194 | 0.003* | 0.706 | PRKAR2A,TBXA2R,CTU1 | |
| GSE43863_TH1_VS_LY6C_LOW_CXCR5NEG_EFFECTOR_CD4_TCELL_DN | 194 | 0.003* | 0.706 | STARD3,STX4,TAPBP | |
| GSE369_IFNG_KO_VS_WT_LIVER_UP | 187 | 0.003* | 0.706 | NCOA2,C2orf68,TBXA2R | |
| GSE40443_INDUCED_VS_TOTAL_TREG_DN | 193 | 0.003* | 0.706 | PRKAR2A,RCN1,B4GALT5 | |
| GSE11961_FOLLICULAR_BCELL_VS_MEMORY_BCELL_DAY7_UP | 193 | 0.003* | 0.706 | STARD3,COL13A1,TAPBP | |
| GSE20727_H2O2_VS_ROS_INHIBITOR_TREATED_DC_UP | 188 | 0.003* | 0.706 | NCOA2,C2orf68,VOPP1 | |
| GSE30962_PRIMARY_VS_SECONDARY_ACUTE_LCMV_INF_CD8_TCELL_DN | 191 | 0.003* | 0.706 | EPHA1,RCN1,VOPP1 | |
| GSE2770_IL12_AND_TGFB_VS_IL4_TREATED_ACT_CD4_TCELL_48H_UP | 191 | 0.003* | 0.706 | AMBRA1,TAPBP,B4GALT5 | |
| *Trimester 1 infections* | | | | |  |
| GSE2706_UNSTIM_VS_8H_LPS_DC_UP | 194 | 1.674E-04* | 0.816 | MSI2,INPP5A,ECHDC1 | |
| GSE43863_NAIVE_VS_LY6C_LOW_CXCR5NEG_CD4_EFF_TCELL_D6_LCMV_UP | 168 | 0.003* | 1 | C5,LRMDA | |
| GSE2706_2H_VS_8H_R848_AND_LPS_STIM_DC_UP | 185 | 0.004* | 1 | MSI2,ECHDC1 | |
| GSE30971_WBP7_HET_VS_KO_MACROPHAGE_4H_LPS_STIM_DN | 186 | 0.004* | 1 | ECHDC1,C5 | |
| GSE5099_DAY3_VS_DAY7_MCSF_TREATED_MACROPHAGE_DN | 159 | 0.004* | 1 | CDK6,MSI2 | |
| GSE22886_UNSTIM_VS_IL2_STIM_NKCELL_DN | 195 | 0.005* | 1 | CDK6,ECHDC1 | |
| GSE5542_IFNG_VS_IFNA_AND_IFNG_TREATED_EPITHELIAL_CELLS_6H_UP | 186 | 0.005* | 1 | CDK6,NMNAT2 | |
| GSE36888_UNTREATED_VS_IL2_TREATED_TCELL_17H_UP | 188 | 0.005* | 1 | ECHDC1,C5 | |
| GSE5099_CLASSICAL_M1_VS_ALTERNATIVE_M2_MACROPHAGE_DN | 156 | 0.005* | 1 | CDK6,MSI2 | |
| GSE8685_IL15_ACT_IL2_STARVED_VS_IL21_ACT_IL2_STARVED_CD4_TCELL_UP | 164 | 0.005* | 1 | MSI2,GRTP1 | |
| GSE39022_LN_VS_SPLEEN_DC_UP | 190 | 0.006* | 1 | CDK6,NMNAT2 | |
| GSE14000_UNSTIM_VS_16H_LPS_DC_UP | 187 | 0.006* | 1 | INPP5A,LRMDA | |
| GSE37301_RAG2_KO_VS_RAG2_AND_ETS1_KO_NK_CELL_UP | 189 | 0.006* | 1 | INPP5A,C5 | |
| GSE25087_TREG_VS_TCONV_FETUS_UP | 188 | 0.006* | 1 | CDK6,C5 | |
| GSE18281_CORTEX_VS_MEDULLA_THYMUS_DN | 170 | 0.006* | 1 | INPP5A,C5 | |
| GSE20366_TREG_VS_NAIVE_CD4_TCELL_DEC205_CONVERSION_UP | 194 | 0.006* | 1 | CDK6,MSI2 | |
| GSE2770_IL12_AND_TGFB_ACT_VS_ACT_CD4_TCELL_2H_DN | 182 | 0.006* | 1 | CDK6,LRMDA | |
| GSE12392_IFNAR_KO_VS_IFNB_KO_CD8_NEG_SPLEEN_DC_DN | 191 | 0.006* | 1 | INPP5A,C5 | |
| GSE5589_LPS_VS_LPS_AND_IL10_STIM_IL10_KO_MACROPHAGE_180MIN_DN | 189 | 0.007* | 1 | CDK6,KCNH5 | |
| GSE2128_C57BL6_VS_NOD_CD4CD8_DP_THYMOCYTE_DN | 195 | 0.007* | 1 | MSI2,ECHDC1 | |
| *Trimester 2 infections* | | | | |  |
| GSE13493_DP_VS_CD8POS_THYMOCYTE_DN | 192 | 3.945e-05* | 0.192 | ITPK1,LIPC,PRKAR2A,RCN1 | |
| GSE39152_BRAIN_VS_SPLEEN_CD103_NEG_MEMORY_CD8_TCELL_DN | 193 | 0.001* | 0.934 | ANKRD11,CLDND1,RCN1 | |
| GSE22601_CD4_SINGLE_POSITIVE_VS_CD8_SINGLE_POSITIVE_THYMOCYTE_DN | 196 | 0.001* | 0.934 | ANKRD11,PRKAR2A,RCN1 | |
| GSE12845_NAIVE_VS_DARKZONE_GC_TONSIL_BCELL_UP | 186 | 0.001* | 0.934 | NCOA2,ANKRD11,ITPK1 | |
| GSE19888_ADENOSINE_A3R_INH_PRETREAT_AND_ACT_BY_A3R_VS_A3R_INH_AND_TCELL_MEMBRANES_ACT_MAST_CELL_DN | 188 | 0.001* | 0.934 | NCOA2,ANKRD11,LIPC | |
| GSE40273_GATA1_KO_VS_WT_TREG_DN | 193 | 0.001* | 0.934 | ANKRD11,ITPK1,LIPC | |
| GSE33292_DN3_THYMOCYTE_VS_TCF1_KO_TCELL_LYMPHOMA_DN | 143 | 0.010* | 1 | ITPK1,RCN1 | |
| GSE2706_R848_VS_R848_AND_LPS_2H_STIM_DC_UP | 168 | 0.011* | 1 | NCOA2,CCDC33 | |
| GSE40274_EOS_VS_FOXP3_AND_EOS_TRANSDUCED_ACTIVATED_CD4_TCELL_UP | 150 | 0.011* | 1 | NCOA2,CLDND1 | |
| GSE41867_DAY15_EFFECTOR_VS_DAY30_MEMORY_CD8_TCELL_LCMV_ARMSTRONG_DN | 175 | 0.011* | 1 | ZCCHC14,IMPG1 | |
| GSE4590_SMALL_VS_VPREB_POS_LARGE_PRE_BCELL_UP | 161 | 0.012* | 1 | NCOA2,PRKAR2A | |
| GSE13411_NAIVE_BCELL_VS_PLASMA_CELL_DN | 176 | 0.013* | 1 | ANKRD11,RCN1 | |
| GSE46606_IRF4_KO_VS_WT_CD40L_IL2_IL5_3DAY_STIMULATED_BCELL_DN | 180 | 0.013* | 1 | ZCCHC14,MAP3K7CL | |
| GSE369_PRE_VS_POST_IL6_INJECTION_SOCS3_KO_LIVER_UP | 187 | 0.013* | 1 | ICAM2,CLDND1 | |
| GSE6269_HEALTHY_VS_STAPH_AUREUS_INF_PBMC_DN | 159 | 0.013* | 1 | ZCCHC14,PRKAR2A | |
| GSE1432_CTRL_VS_IFNG_24H_MICROGLIA_DN | 195 | 0.013* | 1 | CALCOCO2,RCN1 | |
| GSE10325_CD4_TCELL_VS_LUPUS_CD4_TCELL_DN | 189 | 0.014* | 1 | LIPC,MAP3K7CL | |
| GSE22601_IMMATURE_CD4_SINGLE_POSITIVE_VS_CD4_SINGLE_POSITIVE_THYMOCYTE_UP | 186 | 0.014* | 1 | PRKAR2A,CLDND1 | |
| GSE26488_CTRL_VS_PEPTIDE_INJECTION_HDAC7_DELTAP_TG_OT2_THYMOCYTE_UP | 192 | 0.014* | 1 | LIPC,RCN1 | |
| GSE3039_NKT_CELL_VS_ALPHAALPHA_CD8_TCELL_DN | 192 | 0.014* | 1 | ICAM2,CLDND1 | |
| *Trimester 3 infections* | | | | |  |
| GSE15930_STIM_VS_STIM_AND_TRICHOSTATINA_72H_CD8_T_CELL_DN | 189 | 0.001 | 1 | IFIT3,PDGFRB,ZNF444 | |
| GSE34156_UNTREATED_VS_6H_TLR1_TLR2_LIGAND_TREATED_MONOCYTE_DN | 175 | 0.001 | 1 | AKAP8L,BRD3,PDLIM4 | |
| GSE15930_STIM_VS_STIM_AND_IL12_24H_CD8_T_CELL_DN | 186 | 0.001 | 1 | IFIT3,PDGFRB,ZNF444 | |
| GSE17721_CTRL_VS_GARDIQUIMOD_8H_BMDC_DN | 191 | 0.002 | 1 | SNTG2,BRD3,PDLIM4 | |
| GSE10325_CD4_TCELL_VS_LUPUS_CD4_TCELL_UP | 186 | 0.002 | 1 | CUX1,RERE,DNAH17 | |
| GOLDRATH_NAIVE_VS_EFF_CD8_TCELL_UP | 194 | 0.002 | 1 | AKAP8L,RERE,BRD3 | |
| GSE23568_ID3_TRANSDUCED_VS_ID3_KO_CD8_TCELL_UP | 198 | 0.003 | 1 | PDGFRB,ZAP70,PDLIM4 | |
| GSE7460_FOXP3_MUT_VS_WT_ACT_WITH_TGFB_TCONV_DN | 192 | 0.004 | 1 | AKAP8L,RERE,PDLIM4 | |
| GSE14415_NATURAL_TREG_VS_FOXP3_KO_NATURAL_TREG_UP | 149 | 0.007 | 1 | IFIT3,TRPM4 | |
| GSE43863_NAIVE_VS_MEMORY_TH1_CD4_TCELL_D150_LCMV_DN | 147 | 0.008 | 1 | KANK2,SCARF2 | |
| GSE7218_IGM_VS_IGG_SIGNAL_THGOUGH_ANTIGEN_BCELL_DN | 172 | 0.008 | 1 | IFIT3,ZAP70 | |
| GSE21546_ELK1_KO_VS_SAP1A_KO_AND_ELK1_KO_DP_THYMOCYTES_UP | 185 | 0.009 | 1 | IFIT3,LRRFIP1 | |
| GSE2770_UNTREATED_VS_TGFB_AND_IL4_TREATED_ACT_CD4_TCELL_48H_UP | 139 | 0.009 | 1 | ZAP70,SCARF2 | |
| GSE6269_FLU_VS_STREP_PNEUMO_INF_PBMC_UP | 166 | 0.011 | 1 | IFIT3,BRD2 | |
| GSE17721_0.5H_VS_8H_CPG_BMDC_DN | 191 | 0.012 | 1 | PDGFRB,BRD2 | |
| GSE19401_UNSTIM_VS_PAM2CSK4_STIM_FOLLICULAR_DC_DN | 190 | 0.012 | 1 | BRD2,LRRFIP1 | |
| GSE40655_FOXO1_KO_VS_WT_NTREG_DN | 187 | 0.012 | 1 | CUX1,TRPM4 | |
| GSE14415_NATURAL_TREG_VS_FOXP3_KO_NATURAL_TREG_DN | 155 | 0.013 | 1 | AKAP8L,RERE | |
| GSE6269_FLU_VS_E_COLI_INF_PBMC_UP | 152 | 0.013 | 1 | BRD2,LRRFIP1 | |
| GSE15930_STIM_VS_STIM_AND_TRICHOSTATINA_24H_CD8_T_CELL_DN | 194 | 0.013 | 1 | IFIT3,RERE | |
| *P<0.05  **P_FDR_<0.05 |  |  |  |  | |

**Table S7**. Functional look-up analysis of suggestive hits based on previously mentioned traits

| CpG | Trait EWAS catalog | Trait EWAS atlas |
| --- | --- | --- |
| *Total infection sum score* | | |
| cg17050810 | Tissue, age, malathion | Air pollution, carboplatin treatment |
| cg16060163 | Age, rheumatoid arthritis | - |
| cg27466042 | Age, serum high-density lipoprotein cholesterol | - |
| cg04478698 | - | - |
| cg12058372 | Tissue, age, incident COPD, eosinophilia, sex, gestational age, smoking | Aging, asthma, kabuki syndrome, smoking, atopy, allergic sensitization, breast cancer prognosis |
| cg25376660 | Age, rheumatoid arthritis | - |
| cg00702872 | Tissue, age, gestational age, prevalent COPD (self-reported) | Down syndrome |
| cg09130190 | Tissue, age | Placental microbes |
| cg00093220 | Age | - |
| cg00264346 | Tissue, age | - |
| cg26718621 | Tissue, mortality | Mortality, muscle hypertrophy |
| cg03300596 | Age | - |
| cg27426220 | Tissue, Age | - |
| cg02296145 | Age | - |
| cg23962849 | Tissue, age, gestational age | Breast cancer prognosis |
| cg18756291 | Age | - |
| cg01304814 | Tissue | Myopia |
| cg16371518 | Tissue, age, incident COPD, incident type 2 diabetes, smoking, nitrogen dioixide exposure, prevalent type 2 diabetes (self-report), incident liver cirrhosis, alcohol consumption per day | Air pollution |
| cg19334350 | Tissue, sex, infant sex, monozygotic twinning | Down syndrome, sex, infant sex |
| cg10600568 | Age, gestational age | Gestational age |
| cg25839724 | Tissue, age, fetal vs. adult liver, gestational age | Down syndrome, gestational age |
| cg27005246 | Age | - |
| cg05544413 | Tissue, age, incident COPD, sex, alzheimers disease braak stage | SETD1B related syndrome |
| cg16691158 | Tissue, age, incident COPD, sex, clear cell renal carcinoma, incident type 2 diabetes, maternal BMI, nitrogen dioxide exposure, alcohol consumption, lung cancer, OMG (Oligodendrocyte myelin glycoprotein) protein levels | Down syndrome, oral squamous cell carcinoma, lung cancer risk |
| cg13904667 | Tissue, age, incident COPD, incident type 2 diabetes, gestational age, smoking, birth weight, maternal overweight/obesity, incident lung cancer, infant sex, incident liver cirrhosis, LTA protein levels | - |
| cg08337633 | Tissue, clear cell renal carcinoma, gestational age, age 4 vs age 0, primary sjogrens syndrome, pancreatic ductal adenomacarcinoma, age, ageing, prevalent colorectal cancer (self-report) | Papillary thyroid carcinoma, psoriasis, atopy |
| cg08795170 | Age | - |
| cg26068141 | Tissue, age, rheumatoid arthritis | - |
| cg13680337 | Tissue, age, sex, fetal vs adult liver, gestational age, smoking, age*sex, infant sex, liver fat | Ageing, sex, preterm birth, psoriasis, smoking, liver fraction at 10 years, infant sex |
| cg14383355 | Tissue, age, fetal vs adult liver, NEGR1 protein, TGFBR3 protein levels | - |
| cg18738857 | Tissue, age NEO1 protein levels, NCAM1 protein levels | Dilated cardiomyopathy |
| cg19445322 | Tissue, age, sex, monozygotic twinning | - |
| cg08725904 | Tissue, age | Thoracic ossification of the ligamentum flavum |
| *Trimester 1 infection sum score* | | |
| cg12963656 | Tissue, age, incident COPD, clear cell renal carcinoma, fetal vs adult liver, gestational age, smoking, pancreatic ductal adenocarcinoma, smoking, ageing, prevalent chronic pain (self-report), GZMK protein levels, GZMA protein levels | Down syndrome, maternal alcohol consumption, myalgic encephalomyelitis/chronic fatigue syndrome, smoking, systemic lupus erythematosus, gulf war illness |
| cg22430861 | Tissue, age, fetal vs adult liver, gestational age, eosinophilia | Down syndrome |
| cg05544413 | Tissue, age, incident COPD, sex, Alzheimer’s disease braak stage | SETD1B-related syndrome |
| cg16698470 | Tissue, rheumatoid arthritis | Infertility |
| cg01253508 | Tissue, age, incident COPD, alcohol consumption per day | Smoking |
| cg19532454 | age, estimated glomerular filtration rate (eGFR) | - |
| cg23993890 | Tissue, age | - |
| cg12995004 | Tissue, age, HIV infection | Colorectal cancer, adenoma |
| cg02836764 | age, rheumatoid arthritis | Estrogen receptor beta status in colorectal cancer |
| cg02987635 | Tissue, age, gestational age, GZMK protein levels, ALPP protein levels | Atherosclerosis |
| cg23962849 | Tissue, age, gestational age | Breast cancer prognosis |
| cg04725669 | Age, HMGCS2 protein levels | - |
| cg01843018 | Tissue, age, fetal vs adult liver | Maternal PM10 exposure in 4th gestational week |
| cg11886260 | Tissue, age, sex, ADHD | - |
| cg06037095 | Tissue, age, sex | Asthma |
| cg11730722 | Tissue, age | Alcohol consumption |
| cg14518261 | Age, HIV infection | - |
| cg11982176 | Tissue, age | Obesity |
| cg00702872 | Tissue, age, gestational age, prevalent COPD (self-report) | Down syndrome |
| cg23585420 | Tissue, age, gestational age, age 4 vs age 0, Graves' disease | Hepatocellular carcinoma, preterm birth |
| *Trimester 2* *infection sum score* | | |
| cg02854164 | Age, rheumatoid arthritis, total cholesterol to lipids ratio in chylomicrons and XXL-VLDL | - |
| cg09130190 | Tissue, age | Placental microbes |
| cg03987884 | Tissue, sex, gestational age | Smoking |
| cg16691158 | Tissue, age, incident COPD, sex, clear cell renal carcinoma, incident type 2 diabetes, maternal BMI, nitrogen dioixide exposure, alcohol consumption, lung cancer, OMG protein levels | Down syndrome, oral squamous cell carcinoma |
| cg27258283 | Age | Prenatal arsenic exposure |
| cg16060163 | Age, rheumatoid arthritis | - |
| cg11254196 | Age, sex, primary Sjogren’s syndrome, schizophrenia | - |
| cg26289665 | Tissue, age, fetal vs adult liver, gestational age | Inflammatory bowel disease, oral squamous cell carcinoma |
| cg26068141 | Tissue, age, rheumatoid arthritis | - |
| cg12284075 | Age | - |
| cg23667178 | Age, prevalent COPD (self-report), prevalent Parkinson's disease (self-report), prevalent lung cancer (self-report) | - |
| cg20968717 | Tissue, age, smoking, Papuan ancestry | - |
| cg16732175 | Tissue, age | - |
| cg01304814 | Tissue | Myopia |
| cg16047144 | Tissue, fetal vs adult liver, cognitive skills -- verbal score | - |
| cg06033660 | Age | - |
| cg15979035 | Age | - |
| cg27100227 | Age, pancreatic ductal adenomacarcinoma/adjacent nontransformed pancreata, maternal pre-pregnancy BMI | Hepatocellular carcinoma |
| cg05379891 | Age | - |
| cg04643552 | Tissue, age | - |
| cg27530239 | Tissue, age, fetal vs adult liver, eosinophilia, gestational age, birthweight | - |
| cg11170479 | Tissue, age | Inflammatory bowel disease |
| cg16622651 | Age, fetal vs adult liver | - |
| cg21218820 | Tissue, gestational age, sex, maternal BMI, nitrogen dioxide exposure | - |
| *Trimester 3 infection sum score* | | |
| cg02355411 | Tissue, PDGFRA (platelet-derived growth factor receptor alpha) protein levels, incident inflammatory bowel disease | Papillary thyroid carcinoma |
| cg05772104 | Tissue, sex, gestational age, age 4 vs age 0, monozygotic twinning | Down syndrome |
| cg23496331 | Tissue, age, eosinophilia | - |
| cg20756891 | Age | B acute lymphoblastic leukemia, colorectal cancer |
| cg27425784 | Tissue, sex, gestational age, age 4 vs age 0, monozygotic twinning | - |
| cg24919344 | Tissue, age, Papuan ancestry | Prenatal PFOA exposure |
| cg24539060 | Tissue, age, sex | - |
| cg15016481 | Tissue, age, clear cell renal carcinoma, age 4 vs age 0, maternal BMI, nitrogen dioxide exposure | - |
| cg26602975 | Tissue, rheumatoid arthritis | - |
| cg23993005 | Age, HIV infection | - |
| cg20385216 | Tissue, TMEM108 protein levels | - |
| cg15702838 | Tissue, age, clear cell renal carcinoma | - |
| cg26797894 | Sex | Air pollution (PM2.5) |
| cg05845765 | Tissue, age, sex, gestational age, inflammatory bowel disease, alcohol consumption per day | Ancestry |
| cg01364935 | Tissue, age, incident COPD, ageing | Myalgic encephalomyelitis/chronic fatigue syndrome, oral squamous cell carcinoma |
| cg19334350 | Tissue, sex, infant sex, monozygotic twinning, sex | Down syndrome, sex, infant sex |
| cg25703213 | Tissue, age | - |
| cg08684551 | Age, fetal intolerance of labor | - |
| cg05129802 | Tissue, age | - |
| cg13315744 | Tissue, age | Asthma, prenatal arsenic exposure |
| cg22816534 | Age | - |
| cg25569582 | Tissue, age, BOC protein levels | - |
| cg23299919 | Age, sex, infant sex, monozygotic twinning | Sex, kabuki syndrome, multiple sclerosis, colorectal laterally spreading tumor, pre- and post- lenalidomide treatment in patients with myelodysplastic syndrome with isolated deletion (5q), infant sex |
| cg19895164 | Tissue, age | - |
| cg07036524 | Tissue, pancreatic ductal adenocarcinoma, CD209 protein levels, | Hepatocellular carcinoma |
| cg24773324 | Age | - |
| cg25569582 | Tissue, PDGFRA (platelet-derived growth factor receptor alpha) protein levels, incident inflammatory bowel disease | Papillary thyroid carcinoma |

**Table S8**. Total infection sum score and replication of suggestive hits in ALSPAC

| CpG | Beta | SE | P-value |
| --- | --- | --- | --- |
| cg00093220 | 2.580E-04 | 2.848E-04 | 0.365 |
| cg00264346 | -1.250E-04 | 9.907E-05 | 0.207 |
| cg00702872 | 0.002 | 0.002 | 0.348 |
| cg01304814 | -2.350E-04 | 9.187E-05 | 0.010* |
| cg02296145 | -1.600E-04 | 4.640E-04 | 0.730 |
| cg03300596 | -4.49-E-04 | 0.001 | 0.636 |
| cg04478698 | 7.720E-05 | 0.001 | 0.934 |
| cg05544413 | -0.003 | 0.003 | 0.203 |
| cg08337633 | -0.001 | 0.002 | 0.516 |
| cg08725904 | -1.870E-04 | 0.001 | 0.890 |
| cg08795170 | 0.001 | 4.570E-04 | 0.170 |
| cg09130190 | -0.001 | 0.001 | 0.314 |
| cg10600568 | 3.340E-04 | 4.151E-04 | 0.421 |
| cg12058372 | 4.620E-04 | 0.002 | 0.837 |
| cg13680337 | -0.001 | 0.002 | 0.792 |
| cg13904667 | -9.410E-05 | 0.002 | 0.962 |
| cg14383355 | 0.004 | 0.004 | 0.297 |
| cg16060163 | -1.130E-04 | 3.811E-04 | 0.766 |
| cg16371518 | -0.001 | 0.002 | 0.626 |
| cg16691158 | 0.002 | 0.002 | 0.221 |
| cg17050810 | 0.001 | 0.002 | 0.468 |
| cg18738857 | 3.480E-04 | 0.001 | 0.632 |
| cg18756291 | -2.360E-05 | 9.051E-05 | 0.794 |
| cg19334350 | -0.004 | 0.003 | 0.113 |
| cg19445322 | 0.003 | 0.003 | 0.396 |
| cg23962849 | -0.002 | 0.001 | 0.255 |
| cg25376660 | -3.240E-04 | 0.001 | 0.737 |
| cg25839724 | -0.001 | 0.003 | 0.790 |
| cg26068141 | -1.150E-04 | 1.026E-04 | 0.263 |
| cg26718621 | -5.420E-05 | 1.264E-04 | 0.668 |
| cg27005246 | -3.390E-05 | 2.239E-04 | 0.879 |
| cg27426220 | -1.000E-05 | 2.159E-04 | 0.962 |
| cg27466042 | -0.001 | 0.001 | 0.272 |
| *P < 0.05  **P_FDR_ < 0.05 | | | |

**Table S9**. Trimester 1 infection sum score and replication of suggestive hits in ALSPAC

| CpG | Beta | SE | P-value |
| --- | --- | --- | --- |
| cg00702872 | -0.002 | 0.002 | 0.356 |
| cg01253508 | -2.547E-04 | 0.001 | 0.712 |
| cg01843018 | -0.001 | 0.002 | 0.636 |
| cg02836764 | -0.002 | 0.001 | 0.164 |
| cg02987635 | 0.003 | 0.003 | 0.317 |
| cg04725669 | -6.785E-05 | 1.067E-04 | 0.525 |
| cg05544413 | -0.002 | 0.003 | 0.464 |
| cg06037095 | 0.001 | 0.002 | 0.745 |
| cg11730722 | -0.001 | 4.236E-04 | 0.115 |
| cg11886260 | 0.001 | 0.002 | 0.650 |
| cg11982176 | -0.001 | 0.002 | 0.739 |
| cg12963656 | 0.001 | 0.002 | 0.592 |
| cg12995004 | 0.001 | 0.001 | 0.576 |
| cg14518261 | 0.002 | 0.002 | 0.362 |
| cg16698470 | 0.003 | 0.002 | 0.134 |
| cg19532454 | 0.002 | 0.001 | 0.071 |
| cg22430861 | -0.003 | 0.002 | 0.083 |
| cg23585420 | -0.001 | 0.003 | 0.858 |
| cg23962849 | -0.003 | 0.001 | 0.058 |
| cg23993890 | 8.482E-05 | 0.001 | 0.948 |
| *P < 0.05  **P_FDR_ < 0.05 | | | |

**Table S10**. Trimester 2 infection sum score and replication of suggestive hits in ALSPAC

| CpG | Beta | SE | P-value |
| --- | --- | --- | --- |
| cg01304814 | -1.624E-04 | 9.038E-05 | 0.072 |
| cg02854164 | -0.002 | 0.002 | 0.163 |
| cg03987884 | 0.005 | 0.002 | 0.028* |
| cg04643552 | 0.001 | 0.001 | 0.177 |
| cg05379891 | 1.00E-04 | 0.001 | 0.865 |
| cg06033660 | 0.002 | 0.002 | 0.246 |
| cg09130190 | -0.003 | 0.001 | 0.031* |
| cg11170479 | -0.003 | 0.001 | 0.031* |
| cg11254196 | -4.917E-04 | 0.002 | 0.841 |
| cg12284075 | -1.044E-05 | 0.001 | 0.989 |
| cg15979035 | 0.001 | 0.002 | 0.415 |
| cg16047144 | -3.581E-04 | 2.371E-04 | 0.131 |
| cg16060163 | -1.773E-04 | 3.739E-04 | 0.635 |
| cg16622651 | 0.001 | 0.002 | 0.626 |
| cg16691158 | 0.002 | 0.002 | 0.358 |
| cg16732175 | -0.001 | 0.001 | 0.357 |
| cg20968717 | 4.51E-04 | 0.001 | 0.658 |
| cg21218820 | -4.068E-05 | 3.519E-04 | 0.979 |
| cg23667178 | 4.818E-04 | 1.008E-04 | 0.171 |
| cg26068141 | -1.399E-04 | 0.001 | 0.165 |
| cg26289665 | 0.002 | 0.001 | 0.122 |
| cg27100227 | 0.001 | 0.001 | 0.489 |
| cg27258283 | 0.002 | 0.001 | 0.162 |
| cg27530239 | 1.03E-04 | 0.002 | 0.945 |
| *P < 0.05  **P_FDR_ < 0.05 | | | |

**Table S11**. Trimester 3 infection sum score and replication of suggestive hits in ALSPAC

| CpG | Beta | SE | P-value |
| --- | --- | --- | --- |
| cg01364935 | 0.002 | 0.002 | 0.351 |
| cg02355411 | 0.001 | 0.001 | 0.500 |
| cg05129802 | -0.001 | 0.001 | 0.343 |
| cg05772104 | 0.002 | 0.004 | 0.582 |
| cg05845765 | 0.001 | 0.002 | 0.602 |
| cg07036524 | 0.003 | 0.001 | 0.026* |
| cg08684551 | 8.005E-05 | 1.784E-04 | 0.653 |
| cg13315744 | 0.001 | 0.002 | 0.472 |
| cg15016481 | 0.003 | 0.001 | 0.076 |
| cg15702838 | 7.314E-06 | 4.551E-04 | 0.987 |
| cg19334350 | -0.001 | 0.003 | 0.759 |
| cg19895164 | 9.307E-05 | 3.799E-04 | 0.806 |
| cg20385216 | -4.123E-04 | 0.001 | 0.736 |
| cg20756891 | -2.769E-04 | 0.001 | 0.814 |
| cg22816534 | -3.050E-05 | 4.164E-05 | 0.464 |
| cg23299919 | 0.001 | 0.005 | 0.818 |
| cg23496331 | -0.002 | 0.002 | 0.392 |
| cg23993005 | -0.002 | 0.002 | 0.411 |
| cg24539060 | -1.791E-05 | 2.013E-04 | 0.929 |
| cg24773324 | -0.001 | 0.001 | 0.406 |
| cg24919344 | -0.001 | 0.001 | 0.123 |
| cg25569582 | -2.066E-04 | 2.756E-04 | 0.453 |
| cg25703213 | 0.001 | 0.001 | 0.364 |
| cg26602975 | 0.001 | 0.001 | 0.271 |
| cg26797894 | 0.002 | 0.001 | 0.210 |
| cg27425784 | -0.005 | 0.003 | 0.059 |
| *P < 0.05  **P_FDR_ < 0.05 | | | |

**Table S12**. Post hoc analysis: abbreviated infection sum score and suggestive hits (Generation R)

| CpG | Beta | SE | P-value |
| --- | --- | --- | --- |
| cg00093220 | 0.001 | 2.925E-04 | 0.016* |
| cg00264346 | -3.031E-05 | 2.226E-04 | 0.910 |
| cg00702872 | -0.001 | 0.001 | 0.077 |
| cg01304814 | -3.475E-04 | 2.165E-04 | 0.037* |
| cg02296145 | -4.470E-05 | 1.784E-04 | 0.607 |
| cg03300596 | -0.001 | 4.561E-04 | 0.007** |
| cg04478698 | -4.061E-04 | 3.712E-04 | 0.273 |
| cg05544413 | 0.002 | 0.001 | 0.024* |
| cg08337633 | 0.001 | 0.001 | 0.117 |
| cg08725904 | 3.871E-04 | 3.330E-04 | 0.209 |
| cg08795170 | -3.281E-04 | 2.540E-04 | 0.188 |
| cg09130190 | 0.001 | 4.412E-04 | 0.001** |
| cg10600568 | -3.874E-04 | 3.127E-04 | 0.133 |
| cg12058372 | 0.003 | 0.001 | 0.002** |
| cg13680337 | 0.002 | 0.001 | 0.035* |
| cg13904667 | -0.002 | 0.001 | 0.0001** |
| cg14383355 | 0.004 | 0.002 | 0.026* |
| cg16060163 | 0.001 | 0.001 | 0.181 |
| cg16371518 | 0.001 | 4.361E-04 | 0.015* |
| cg16691158 | -0.001 | 0.001 | 0.145 |
| cg17050810 | 0.001 | 3.548E-04 | 0.034* |
| cg18738857 | -0.001 | 0.001 | 0.145 |
| cg18756291 | -3.874E-04 | 1.489E-04 | 0.004** |
| cg19334350 | -0.003 | 0.002 | 0.089 |
| cg19445322 | -0.003 | 0.001 | 0.030* |
| cg23962849 | 5.543E-04 | 4.163E-04 | 0.149 |
| cg25376660 | -2.529E-04 | 3.633E-04 | 0.479 |
| cg25839724 | 0.002 | 0.001 | 0.050* |
| cg26068141 | 2.057E-04 | 9.449E-05 | 0.213 |
| cg26718621 | 4.984E-04 | 1.596E-04 | 0.001** |
| cg27005246 | 1.315E-04 | 2.282E-04 | 0.540 |
| cg27426220 | 2.353E-04 | 2.849E-04 | 0.457 |
| cg27466042 | 6.423E-04 | 3.875E-04 | 0.104 |
| *P < 0.05  **P_FDR_ < 0.05 | | | |

**Table S13**. ENR-based weights for MPS-total infections

| CpG | Beta |
| --- | --- |
| cg00093220 | 0.364 |
| cg25839724 | 0.240 |
| cg14383355 | 0.130 |
| cg25376660 | -0.011 |
| cg04478698 | 0.002 |
| cg08725904 | 0.909 |
| cg08795170 | -1.059 |
| cg16691158 | -0.013 |
| cg17050810 | 0.518 |
| cg08337633 | 0.014 |
| cg19334350 | 0.010 |
| cg27426220 | 0.201 |
| cg13680337 | 0.172 |
| cg09130190 | 0.430 |
| cg16371518 | 0.134 |
| cg03300596 | 0.068 |
| cg16060163 | 0.007 |
| cg27466042 | 0.152 |
| cg10600568 | 0.116 |
| cg13904667 | -0.357 |
| cg01304814 | -0.128 |
| cg19445322 | -0.061 |
| cg26718621 | 0.050 |
| cg05544413 | 0.022 |
| cg26068141 | 0.058 |
| cg02296145 | -0.737 |
| cg00264346 | 1.699 |
| cg18738857 | -0.034 |
| cg23962849 | 0.122 |
| cg12058372 | 0.001 |

**Table S14**. ENR-based weights for MPS-trimester 1 infections

| CpG | Beta |
| --- | --- |
| cg23993890 | -0.094 |
| cg06037095 | -0.085 |
| cg12963656 | 0.088 |
| cg19532454 | -0.038 |
| cg01253508 | -0.262 |
| cg12995004 | 0.098 |
| cg00702872 | 0.023 |
| cg04725669 | -0.044 |
| cg01843018 | -0.077 |
| cg02836764 | -0.050 |
| cg02987635 | 0.061 |
| cg11730722 | 0.073 |
| cg16698470 | -0.056 |
| cg11982176 | 0.204 |
| cg11886260 | -0.063 |
| cg23585420 | 0.005 |
| cg22430861 | 0.064 |
| cg05544413 | 0.079 |
| cg14518261 | 0.066 |
| cg23962849 | 0.008 |

**Table S15**. ENR-based weights for MPS-trimester 2 infections

| CpG | Beta |
| --- | --- |
| cg05379891 | -0.025 |
| cg16691158 | 0.004 |
| cg16622651 | -0.059 |
| cg11254196 | 0.027 |
| cg27100227 | 0.051 |
| cg09130190 | 0.044 |
| cg11170479 | 0.046 |
| cg15979035 | 0.034 |
| cg04643552 | -0.053 |
| cg12284075 | -0.028 |
| cg20968717 | -0.004 |
| cg02854164 | 0.003 |
| cg27258283 | 0.003 |
| cg16047144 | -0.047 |
| cg16060163 | 0.0208 |
| cg27530239 | 0.060 |
| cg03987884 | 0.082 |
| cg16732175 | 0.003 |
| cg26289665 | -0.020 |
| cg01304814 | -0.121 |
| cg23667178 | -0.004 |
| cg26068141 | 0.057 |
| cg21218820 | -0.014 |
| cg06033660 | -0.032 |

**Table S16**. ENR-based weights for MPS-trimester 3 infections

| CpG | Beta |
| --- | --- |
| cg05772104 | -0.015 |
| cg13315744 | 0.471 |
| cg15016481 | -0.067 |
| cg26797894 | -0.045 |
| cg20385216 | -0.460 |
| cg20756891 | -0.020 |
| cg23496331 | 0.710 |
| cg24919344 | -0.742 |
| cg05845765 | 0.059 |
| cg19334350 | -0.074 |
| cg25569582 | 0.074 |
| cg23993005 | 0.032 |
| cg26602975 | -0.136 |
| cg01364935 | -0.124 |
| cg05129802 | -0.030 |
| cg07036524 | -0.379 |
| cg02355411 | -0.168 |
| cg19895164 | 0.256 |
| cg27425784 | -0.124 |
| cg25703213 | 0.445 |
| cg08684551 | 0.357 |
| cg15702838 | 4.130E-04 |
| cg22816534 | -0.030 |
| cg23299919 | 0.018 |
| cg24539060 | -0.279 |
| cg24773324 | 0.113 |

**Table S17**. Methylation profile score for infections and child outcomes (total infections)

| Outcome | Standardized beta | SE | P-value |
| --- | --- | --- | --- |
| *Generation R test set (n=476)* | | | |
| CBCL internalizing problems score (age 14) | 0.225 | 0.303 | 0.225 |
| CBCL externalizing problems score (age 14) | 0.303 | 0.269 | 0.316 |
| CBCL total behavioral problems score (age 14) | 0.660 | 0.893 | 0.457 |
| BMI (age 14) | 0.081 | 0.062 | 0.191 |
| Asthma (age 14) | -0.092 | 0.195 | 0.636 |
| *ALSPAC data set (n=864*) | | | |
| SDQ – total difficulties (age 15) | -0.018 | 0.040 | 0.642 |
| SDQ – emotional problems (age 15) | -0.004 | 0.046 | 0.936 |
| SDQ – hyperactivity problems (age 15) | -0.053 | 0.045 | 0.241 |
| SDQ – conduct problems (age 15) | -0.058 | 0.046 | 0.208 |
| BMI (age 17) | -0.018 | 0.040 | 0.642 |
| Asthma (age 13) | -0.022 | 0.094 | 0.812 |
| *P < 0.05  **P_FDR_ < 0.05 | | | |

**Table S18**. Methylation profile score for infections and child outcomes (trimester 1 infections)

| Outcome | Standardized beta | SE | P-value |
| --- | --- | --- | --- |
| *Generation R test set (n=476)* | | | |
| CBCL internalizing problems score (age 14) | 0.117 | 0.303 | 0.698 |
| CBCL externalizing problems score (age 14) | -0.116 | 0.269 | 0.666 |
| CBCL total behavioral problems score (age 14) | -0.248 | 0.893 | 0.781 |
| BMI (age 14) | -0.008 | 0.060 | 0.895 |
| Asthma (age 14) | -0.066 | 0.183 | 0.721 |
| *ALSPAC data set (n=864*) | | | |
| SDQ – total difficulties (age 15) | -0.062 | 0.046 | 0.170 |
| SDQ – emotional problems (age 15) | -0.081 | 0.047 | 0.087 |
| SDQ – hyperactivity problems (age 15) | -0.100 | 0.046 | 0.029* |
| SDQ – conduct problems (age 15) | -0.119 | 0.046 | 0.011* |
| BMI (age 17) | 0.042 | 0.043 | 0.334 |
| Asthma (age 13) | 0.072 | 0.104 | 0.488 |
| *P < 0.05  **P_FDR_ < 0.05 | | | |

**Table S19**. Methylation profile score for infections and child outcomes (trimester 2 infections)

| Outcome | Standardized beta | SE | P-value |
| --- | --- | --- | --- |
| *Generation R test set (n=476)* | | | |
| CBCL internalizing problems score (age 14) | 0.274 | 0.314 | 0.382 |
| CBCL externalizing problems score (age 14) | 0.288 | 0.278 | 0.302 |
| CBCL total behavioral problems score (age 14) | 1.229 | 0.922 | 0.183 |
| BMI (age 14) | 0.031 | 0.061 | 0.610 |
| Asthma (age 14) | -0.271 | 0.200 | 0.175 |
| *ALSPAC data set (n=864*) | | | |
| SDQ – total difficulties (age 15) | -0.015 | 0.044 | 0.739 |
| SDQ – emotional problems (age 15) | -0.033 | 0.045 | 0.047* |
| SDQ – hyperactivity problems (age 15) | -0.043 | 0.044 | 0.329 |
| SDQ – conduct problems (age 15) | -0.039 | 0.045 | 0.376 |
| BMI (age 17) | 0.012 | 0.037 | 0.738 |
| Asthma (age 13) | 0.025 | 0.088 | 0.778 |
| *P < 0.05  **P_FDR_ < 0.05 | | | |

**Table S20**. Methylation profile score for infections and child outcomes (trimester 3 infections)

| Outcome | Standardized beta | SE | P-value |
| --- | --- | --- | --- |
| *Generation R test set (n=476)* | | | |
| CBCL internalizing problems score (age 14) | -0.042 | 0.312 | 0.892 |
| CBCL externalizing problems score (age 14) | 0.130 | 0.277 | 0.636 |
| CBCL total behavioral problems score (age 14) | 0.268 | 0.918 | 0.770 |
| BMI (age 14) | 0.123 | 0.061 | 0.044* |
| Asthma (age 14) | -0.014 | 0.196 | 0.944 |
| *ALSPAC data set (n=864*) | | | |
| SDQ – total difficulties (age 15) | -0.025 | 0.045 | 0.580 |
| SDQ – emotional problems (age 15) | 0.033 | 0.047 | 0.478 |
| SDQ – hyperactivity problems (age 15) | 0.031 | 0.046 | 0.496 |
| SDQ – conduct problems (age 15) | 0.013 | 0.046 | 0.774 |
| BMI (age 17) | 0.043 | 0.038 | 0.256 |
| Asthma (age 13) | 0.033 | 0.092 | 0.722 |
| *P < 0.05  **P_FDR_ < 0.05 | | | |

**Table S21**. Prenatal exposure to infections and residual gestational age acceleration – Generation R

| Timing of infection | Model | Standardized beta | SE | P-value |
| --- | --- | --- | --- | --- |
| *Bohlin clock* | | | | |
| Total infection sum score |  |  |  |  |
|  | Model 1 | 0.020 | 0.020 | 0.234 |
|  | Model 2 | 0.030 | 0.015 | 0.064 |
| Trimester 1 infection sum score |  |  |  |  |
|  | Model 1 | 0.010 | 0.020 | 0.496 |
|  | Model 2 | 0.010 | 0.015 | 0.463 |
| Trimester 2 infection sum score |  |  |  |  |
|  | Model 1 | 0.000 | 0.021 | 0.850 |
|  | Model 2 | 0.010 | 0.016 | 0.497 |
| Trimester 3 infection sum score |  |  |  |  |
|  | Model 1 | 0.020 | 0.019 | 0.405 |
|  | Model 2 | 0.030 | 0.015 | 0.052 |
| *450K/EPIC overlap clock* | | | | |
| Total infection sum score |  |  |  |  |
|  | Model 1 | 0.000 | 0.021 | 0.933 |
|  | Model 2 | 0.000 | 0.017 | 0.868 |
| Trimester 1 infection sum score |  |  |  |  |
|  | Model 1 | -0.010 | 0.020 | 0.714 |
|  | Model 2 | -0.010 | 0.016 | 0.695 |
| Trimester 2 infection sum score |  |  |  |  |
|  | Model 1 | -0.030 | 0.021 | 0.156 |
|  | Model 2 | -0.020 | 0.016 | 0.172 |
| Trimester 3 infection sum score |  |  |  |  |
|  | Model 1 | -0.010 | 0.020 | 0.797 |
|  | Model 2 | 0.010 | 0.016 | 0.560 |
| *P < 0.05  **P_FDR_ < 0.05 | | | | |

**Table S22**. Prenatal exposure to infections and residual gestational age acceleration – ALSPAC

| Timing of infection | Model | Standardized beta | SE | P-value |
| --- | --- | --- | --- | --- |
| *Bohlin clock* | | | | |
| Total infection sum score |  |  |  |  |
|  | Model 1 | 0.005 | 0.035 | 0.885 |
|  | Model 2 | 0.005 | 0.035 | 0.886 |
| Trimester 1 infection sum score |  |  |  |  |
|  | Model 1 | -0.048 | 0.035 | 0.170 |
|  | Model 2 | -0.048 | 0.035 | 0.172 |
| Trimester 2 infection sum score |  |  |  |  |
|  | Model 1 | 0.057 | 0.037 | 0.120 |
|  | Model 2 | 0.057 | 0.037 | 0.122 |
| Trimester 3 infection sum score |  |  |  |  |
|  | Model 1 | 0.016 | 0.035 | 0.643 |
|  | Model 2 | 0.017 | 0.036 | 0.637 |
| *450K/EPIC overlap clock* | | | | |
| Total infection sum score |  |  |  |  |
|  | Model 1 | -0.090 | 0.036 | 0.014* |
|  | Model 2 | -0.090 | 0.036 | 0.014* |
| Trimester 1 infection sum score |  |  |  |  |
|  | Model 1 | -0.077 | 0.036 | 0.033* |
|  | Model 2 | -0.077 | 0.036 | 0.033* |
| Trimester 2 infection sum score |  |  |  |  |
|  | Model 1 | -0.001 | 0.036 | 0.974 |
|  | Model 2 | -0.001 | 0.036 | 0.980 |
| Trimester 3 infection sum score |  |  |  |  |
|  | Model 1 | -0.063 | 0.035 | 0.074 |
|  | Model 2 | -0.064 | 0.035 | 0.071 |
| *P < 0.05  **P_FDR_ < 0.05 | | | | |

**REFERENCES**

1. Kruithof CJ, Kooijman MN, van Duijn CM, Franco OH, de Jongste JC, Klaver CCW, et al. The Generation R Study: Biobank update 2015. Eur J Epidemiol. 2014 Dec;29(12):911–27.

2. Kooijman MN, Kruithof CJ, van Duijn CM, Duijts L, Franco OH, van IJzendoorn MH, et al. The Generation R Study: design and cohort update 2017. Eur J Epidemiol. 2016 Dec;31(12):1243–64.

3. Lehne B, Drong AW, Loh M, Zhang W, Scott WR, Tan ST, et al. A coherent approach for analysis of the Illumina HumanMethylation450 BeadChip improves data quality and performance in epigenome-wide association studies. Genome Biol. 2015 Feb 15;16(1):37.

4. Aryee MJ, Jaffe AE, Corrada-Bravo H, Ladd-Acosta C, Feinberg AP, Hansen KD, et al. Minfi: a flexible and comprehensive Bioconductor package for the analysis of Infinium DNA methylation microarrays. Bioinforma Oxf Engl. 2014 May 15;30(10):1363–9.

5. Relton CL, Gaunt T, McArdle W, Ho K, Duggirala A, Shihab H, et al. Data Resource Profile: Accessible Resource for Integrated Epigenomic Studies (ARIES). Int J Epidemiol. 2015 Aug;44(4):1181–90.

6. Min JL, Hemani G, Davey Smith G, Relton C, Suderman M. Meffil: efficient normalization and analysis of very large DNA methylation datasets. Bioinforma Oxf Engl. 2018 Dec 1;34(23):3983–9.

7. Stevenson AJ, McCartney DL, Hillary RF, Campbell A, Morris SW, Bermingham ML, et al. Characterisation of an inflammation-related epigenetic score and its association with cognitive ability. Clin Epigenetics. 2020 Jul 27;12(1):113.
